# Balancing risks and rewards of alternate strategies in the seaward extent, duration and timing of fjord use in contemporary anadromy of brown trout (*Salmo trutta*)

**DOI:** 10.1186/s12862-023-02179-x

**Published:** 2024-02-29

**Authors:** K. L. Hawley, H. A. Urke, T. Kristensen, T. O. Haugen

**Affiliations:** 1 Faculty of Environmental Sciences and Natural Resource Management, The Norwegian University of Life Sciences, Høgskoleveien 12, 1433 Ås, Norway; 2 AquaLife R&D, Havnegata 9, 7010 Trondheim, Norway; 3 Faculty of Biosciences and Aquaculture, Nord University, Universitetsalléen, 8049 Bodø, Norway

**Keywords:** Acoustic telemetry, Anadromy, Anthropogenic environmental change, Conditional Arnason-Schwarz, Fitness, Mark-recapture, Migration, Survival

## Abstract

**Background:**

Anadromy comprises a successful life-cycle adaptation for salmonids, with marine migration providing improved feeding opportunities and thus improved growth. These rewards are balanced against costs from increased energy expenditure and mortality risk. Anthropogenic-induced environmental changes that reduce benefits and/or increase costs of migration e.g., aquaculture and hydropower, may therefore result in adaptations disfavouring anadromy. We tagged brown trout (*Salmo trutta*) smolts (*N* = 175) and veteran migrants (*N* = 342), from five adjacent riverine populations located in Sognefjorden, the longest Norwegian fjord-system supporting anadromous brown trout populations (209 km). Over four years, 138 acoustic telemetry receivers were deployed to track migrations of tagged individuals from freshwater and throughout Sognefjorden. Detected movements were used to fit migration models and multi-state mark-recapture models of survival and movement for each life-stage. Seaward migration distance was modelled to examine the fitness consequences from alternate migration strategies, with these models used to simulate the extent of fjord-use by individuals and accompanying growth, fecundity and survival consequences. We compared these findings with mark-recapture data collected prior to aquaculture and hydropower development.

**Results:**

The telemetry data revealed that the outermost-fjord region was utilised by all populations albeit by few individuals. However, historical recaptures were located at a greater distance from the river mouth (87.7 ± 70.3 km), when compared to maximum migration distances of present-day counterparts (58.6 ± 54.9 km). River of origin influenced observed migratory behaviour and differential survival was estimated for each population and life-stage. The simulations based on telemetry-data models revealed a 30% and 23% difference in survival among populations for smolts and veteran migrants, respectively. At the individual-level, a long-distance migration strategy was rewarded with enhanced fecundity. However, the main contribution to population-level fecundity was overwhelmingly derived from middle-distance migrants, due to higher mortality rates and limited numbers of long-distant migrants.

**Conclusions:**

We conclude that present-day anadromy is precarious, but potential risk varies considerably between life-stages and populations, even within a single fjord system. Our findings suggest that selection for extended migration is under pressure, we therefore stress the importance of monitoring and management actions to secure genetic variation pertinent to preserve fitness gains of anadromy.

**Supplementary Information:**

The online version contains supplementary material available at 10.1186/s12862-023-02179-x.

## Introduction

Migration provides the opportunity for exploitation of heterogeneities between spatially separated environments [1–3]. Anadromy comprises a successful life-cycle adaptation for salmonids migrating between adjacent low-risk, nutrient-poor habitats (streams, rivers and lakes) and high-risk, nutrient-rich habitats (marine waters) [4, 5]. Natural selection for, or against expression of anadromy has occurred since the last ice-age, for some 10,000 years [6], yet in relative terms, recent human activities have altered the planet’s environment to an extent worth coining a new geological epoch: the Anthropocene [7]. Anadromous salmonids are greatly impacted by these anthropogenic environmental changes, due to their dependence on several different habitats and connectivity between them [8]. Since the degree of change varies considerably both in type and extent among river systems and marine habitats, selection factors acting in favour of anadromy and those that counteract them may vary accordingly [9]. Alterations of coastal and marine environments, coupled with modifications in freshwater, may alter the costs and benefits of preferential habitat selection. Thus, anthropogenic-induced change may modify fitness landscapes, altering expression of life-history strategies, which could ultimately result in adaptations disfavouring anadromy [10, 11].

The brown trout (*Salmo trutta*) has been described as a 'plastic' species that can exist in numerous forms in response to its evolutionary history and the spatial and temporal conditions in its local environment [5, 12]. Juvenile brown trout hatch in freshwater tributaries in the spring and spend several years in their natal stream before either migrating to lakes (potamodromous) or the sea (anadromous), depending upon availability of suitable habitat [13, 14]. However, in many cases, a proportion of the population will remain in freshwater fluvial habitat and assume residency. Thus, migrants and residents share natal nursery habitat but migrants undertake non-breeding transitions into productive feeding habitats, where migrants attain greater body size [15]. Brown trout are iteroparous, meaning that they can spawn several times after sexual maturity [16, 17], with anadromous brown trout that survive spawning commonly referred to as kelts. The spawning status of females is an important predictor of recruitment, as larger repeat spawners produce not only a greater quantity of eggs but also larger eggs often spawned in more favourable habitat, and therefore early growth and viability of offspring increases with maternal age [18–20]. However the potential gains of increased growth and in turn reproductive success are offset against potential risks accompanying anadromy, where the benefits must outweigh the costs for the development and preservation of anadromy [1]. In anadromous brown trout costs may include increased predation risk, exposure to novel pathogens as well as the additional energy expenditures for movement and smolting and ultimately a greater risk of mortality [21]. However, the spatial–temporal variation in the nature and scope of anadromy in brown trout remains poorly understood, and has been described as a continuum from freshwater residency to complete anadromy [15, 22]. Amongst anadromous individuals the distance and duration of the marine migration varies extensively, both among and within populations. Typically, brown trout at sea remain close to the surface and reside in near-shore coastal and estuarine waters for just a few weeks over the summer months [23–25], and amongst those that undertake a short sea-sojourn, some may return as immature juveniles in order to overwinter in freshwater, without returning to spawn [15, 17]. In contrast however, examples of brown trout migrating extensive distances from their natal rivers have also been documented [26, 27], as well as year-round sea migrations being commonly observed [28–30]. Migration between freshwater nursery grounds and marine feeding areas is considered a critical event in salmonid life histories [31], with in-river environmental cues (water discharge and/or temperature) commonly linked to the timing of downstream migration, as these cues may provide a signal of favourable conditions at sea [31, 32]. Within the marine environment, habitat use and behaviour is reported as highly variable. Physical fjord features [22, 33], fish size [34–36], sex [37, 38] and physiological condition [39–42] are listed as some of the most influential factors on migratory behaviour.

In recent decades, anthropogenic environmental change has negatively impacted anadromous brown trout populations in Europe [21, 43]. Overfishing, habitat degradation (e.g., hydropower development, riverbed regulations), climate change and water pollution are listed amongst the main disturbances affecting brown trout populations [21]. Increasing salmonid aquaculture activity has compounded densities of the parasitic salmon-lice (*Lepeophtheirus salmonis*) in many fjords [6, 44, 45], with anadromous brown trout prone to infection by this parasite since they mainly utilise marine areas in close vicinity to aquaculture facilities and during periods of high salmon-lice densities [44]. Therefore, anadromous brown trout experience increased marine mortality and reduced individual growth in fjords with extensive salmon farming activity [46–48]. In anadromous brown trout populations, juvenile mortality in the freshwater phase is often density dependent and may therefore have a population regulating effect that will impact the number of smolts descending from the river [16]. In contrast, mortality in the marine environment is density independent [17]. Hence, it is not believed that there are compensatory mechanisms for additional mortality in the marine phase (but see [49]). As anadromous brown trout are predominantly female, additional marine mortality has an augmented potential to negatively affect population recruitment [6, 38, 50].

**Fig. 1:**
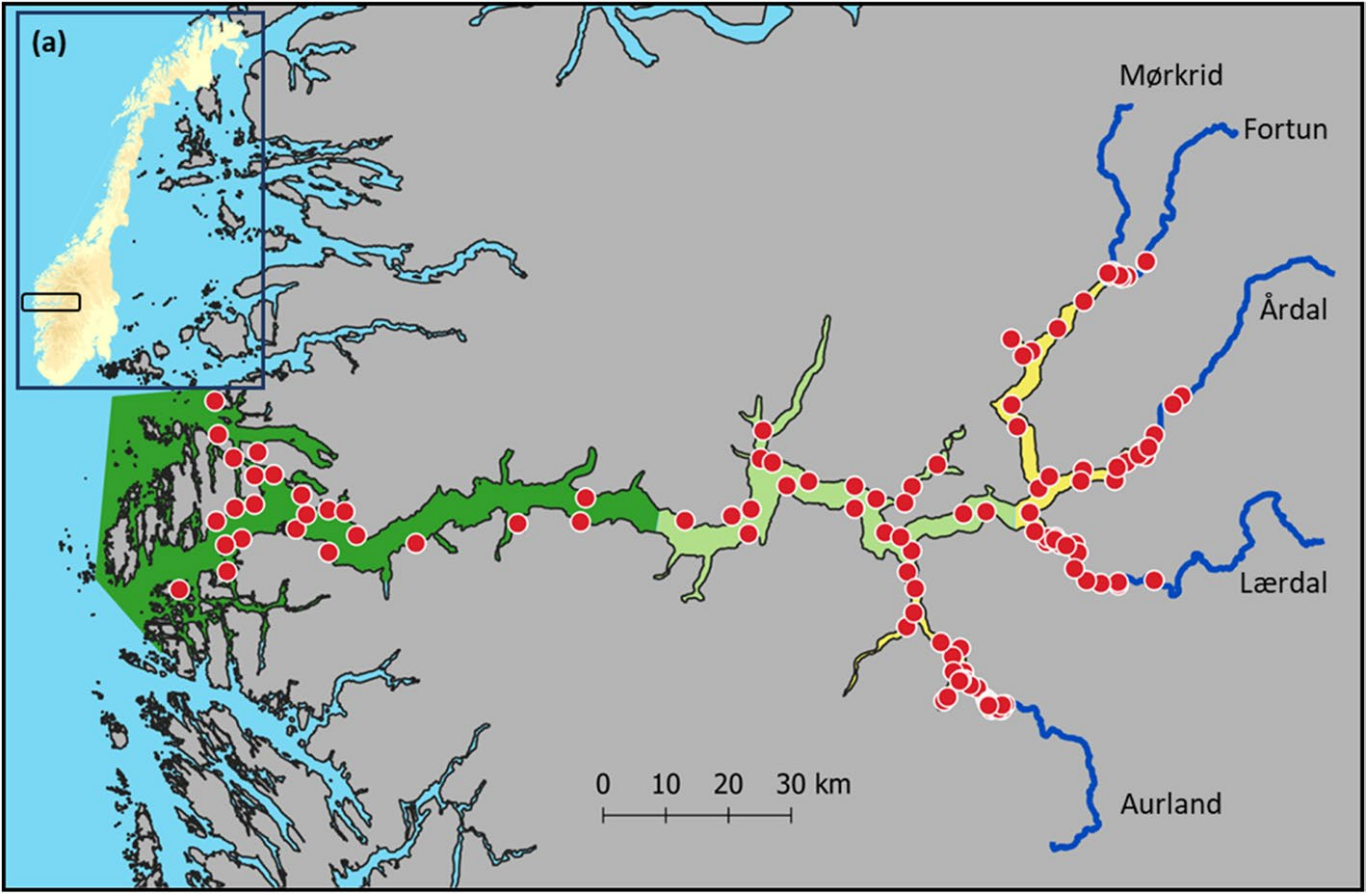
Locations of the five river arms (Aurland, Lærdal, Årdal, Fortun and Mørkrid) and 138 passive receivers (red dot = VR2W) distributed throughout the study area; Sognefjorden, Norway between September 2012 – October 2015. Fjord regions are coloured according to zone; yellow = inner-fjord, light green = mid-fjord, dark green = outer-fjord. **a** Insert illustrating the location of Sognefjorden on the west coast of Norway. The map was produced by the authors

The current study was conducted in Norway’s longest fjord, Sognefjorden (209 km, Fig. 1). The scale and semi-enclosed nature of Sognefjorden creates a unique prospect in which to distinguish alternate strategies of anadromy based upon the expression of seaward migration extent. Historically, anadromous brown trout in Sognefjorden, sought feeding areas in the outer reaches of the fjord system and along the open coast-line [51], this corresponds to the area where most aquaculture facilities are now located. In the county Vestland, in which Sognefjorden is located, sea-based salmonid aquaculture production equated to ca. 3.6 million fish during 2015 (numbers reported by the Directorate of Fisheries). In contrast, the inner half of Sognefjorden has never been intensively utilised for aquaculture, and since 2007 has been granted protection as a national salmon fjord with subsequent abandonment of sea-based aquaculture. Consequently, the outer fjord region receives substantially increased amounts of infectious salmon-lice larvae from open aquaculture systems [45]. Concurrently considerable hydropower development since the 1960s has altered the volume, timing, and temperature of freshwater input to the inner parts of the fjord system, which in turn has altered the salinity profile and productivity of the inner-fjord region [52]. Despite the intensity of anthropogenic alterations in the region, relatively little monitoring has been undertaken to assess the current ecological status of the fjord, nor assessment of the potential environmental impacts of these alterations. We utilized acoustic telemetry, a method which provides spatial–temporal information of marked individuals *in-situ* (e.g., [53]), to follow anadromous brown trout during smolt and veteran migrant life-stages, from freshwater nursery and spawning grounds to saltwater feeding areas. We applied a multi-state mark-recapture model to simultaneously estimate temporal- survival, recapture and transition probabilities between pre-determined habitat zones [54, 55]. To explore survival, growth and fitness consequences from different migration pathways, simulated data was generated based on fitted telemetry-data and mark-recapture models, producing spatial–temporal trajectories of individual brown trout habitat use, for each population and life-stage. By selecting five different natal populations that drain into the same enclosed fjord system, the growth potential in the marine environment was effectively fixed or (close to) equal for all five populations. This enabled population level inferences of survival and fecundity resulting from alternate strategies along the migratory continuum of brown trout. To compare contemporary and historical forms of brown trout anadromy in Sognefjorden, we used growth data (from fish scales) collected for risk-assessment and stock value estimation purposes, prior to the initial hydropower installation in the region and compared this to growth of the present sampled brown trout. We also analysed historical mark-recapture data, collected from catches of in-river tagged fish from the now largely abandoned bag-net fisheries, to compare contemporary migration distance with that of veteran migrant brown trout prior to aquaculture development in Sognefjorden.

We hypothesised that the maximum seaward extent and duration of anadromy would vary among populations, and that environmental conditions (water discharge) in each river would affect the propensity and timing of migration. We anticipated that the risk of migration would be greatest for smolts, but that the degree of risk would vary spatially and temporally within the fjord, for both smolt and veteran migrant anadromous brown trout. We also postulated that alternate strategies in the maximum seaward migration distance would be observed in individual brown trout, and that the cost (estimated as mortality) of individual selection in this extent of anadromy would vary both among and within populations. However, we anticipated that mortality would be balanced against reproduction gains (growth and fecundity) in the selection of these strategies, at both the individual and population level. Ultimately, owing to recent anthropogenic-induced changes in the fjord-coast environment, we postulated that seaward migration distances have declined when compared to historical mark-recapture data of anadromous brown trout from the region.

## Materials and methods

### Study system

The study was conducted over four consecutive years (2012–2015), and in five adjacent rivers draining into four distinct fjord arms of Sognefjorden (Aurland, Lærdal, Årdal, Fortun, Mørkrid), located on the west coast of Norway (Fig. 1, Table 1). Sognefjorden is Norway’s longest and deepest fjord, connected to the open sea by a 209 km long strait, with a maximum depth of 1.3 km. Despite its length, Sognefjorden is relatively narrow (mean width ca. 4.5 km) and is semi-enclosed by an underwater sill at a depth of ca. 160 m which is located at the mouth of the fjord. It is the largest fjord system in the world that supports populations of anadromous brown trout. The area is characterised by steep mountainsides, great depths, and cold freshwater input from partially glaciated, high-altitude catchments. Three of the river stretches that support anadromous trout include a lake which must be traversed to reach the fjord (Aurland, Årdal, Fortun), and in these populations, brown trout is the dominant fish species. In the river Lærdal, the Atlantic salmon (*Salmo salar*) stock has historically been the largest in the region, but the river has also supported a significant brown trout population. This river was infected with the parasite *Gyrodactylus salaris* in 1996, and the following year was subjected to rotenone treatments, resulting in a (near) complete loss of juvenile salmonids as well as considerable numbers of overwintering veteran migrant brown trout. Mørkrid, in which Atlantic salmon is the dominant species, and the larger river Fortun drain into the same inner-most fjord arm of Sognefjorden, with these populations located furthest from the open coast (> 200 km) (Fig. 1, Table 1).

**Table 1:** The river length and lake area that support anadromous brown trout, as well as the catchment area of each study population, all of which drain into Sognefjorden, Norway. An overview of the *Salmo trutta* smolts and veteran migrants marked with acoustic tags, during the sampling years 2012 – 2015 is also stated

River	Aurland	Lærdal	Årdal	Fortun	Mørkrid
Catchment area (km^2^)	804	1184	981	508	288
Mean annual discharge (m^3^ s^−1^)	37.6	36.4	46.1	28.5	12
River length (km)	10	25	30	16	9.5
Lake area (km^2^)	1.9	0	7.5	0.62	0
Distance from river mouth to outermost receiver (km)	160	167	181	209	209
Number smolts detected (%)	69 (81)	12 (63)	42 (84)	40 (80)	12 (80)
Number migrant smolts (%)	37 (54)	11 (92)	22 (52)	23 (58)	7 (58)
Total length (cm) (mean, range)	20.1, 12 – 29	19.5, 12 – 27	15.6, 12 – 27	18.2, 11 – 26	19.6, 15 – 24
Fulton's K (mean ± SD (N))	0.80 ± 0.16 (34)	0.78 ± 0.07 (6)	0.79 ± 0.11 (10)	0.86 ± 0.08 (16)	NA (0)
N veteran migrant brown trout detected (%)	85 (96)	118 (98)	76 (99)	63 (91)	NA
N migrant veteran brown trout (%)	49 (58)	63 (54)	52 (68)	41 (65)	NA
N return to freshwater (%)	41 (84)	53 (84)	45 (87)	23 (56)	NA
Total length (cm) (mean, range)	39.9, 24 –76	44.3, 22 – 74	40.7, 25 – 69	48.9, 26 – 86	NA

Migrant individuals were detected within the fjord. The number of migrant brown trout returning to freshwater are presented for veteran migrant brown tout only, due to battery limitations (see Table S1 for tag specifications) the number of returning smolts are invalid. Migrant brown trout were categorised as returning if the final annual detection were recorded in zone F, where multiple years of detection data were generated (*N* = 144), only the first year of data is considered

### Fish sampling- contemporary samples

Immature *Salmo trutta* were sampled by electric fishing in the five river arms during April (2012 – 2015, Table 1), and characterised according to life-history based on external characters [56]. Only ‘smolt-like’ individuals (elongated in shape and in the process of becoming silver in colour) were processed, ‘resident’ phenotypes were returned directly and not handled further. Veteran migrant brown trout, i.e., fish that have previously undertaken at least one sea-sojourn, were captured in freshwater using rod and line during September – October (2012 – 2014). These larger fish were sampled from four of the river arms (not Mørkrid) and held at local hatcheries for 1 – 3 days pending tagging and sampling. Fish were kept in tanks (1000 – 2000 L) with sufficient water flow (30 – 40 L min^−1^) and transported by car (10 – 30 min, in 300 – 400 L tanks filled with river water) to the tagging sites. Smolts were caught 8 – 24 h prior to tagging and held in 40 L tanks with flowing water (5 – 7 L min^−1^). All brown trout were anaesthetised prior to surgery using tricaine methane sulphonate (MS-222, 60 mg L^−1^, ca. 4 min immersion in aqueous solution). After reaching full anaesthesia, fish were placed ventral side up into a V-shaped surgical tray with a continuous anaesthetic flow over the gills for the entire procedure (40 mg L^−1^ MS-222). Tags were surgically inserted into the body cavity through a small incision posterior to the pelvic girdle, which was closed with three interrupted double surgical knots using a non-absorbing 4/0 monofilament suture (www.resorba.com) and sealed with a tissue adhesive (monomeric n-butyl-2-cyanoacrylate, Histoacryl®). Scales were taken to estimate growth and a small section of the pelvic fin was taken for DNA analyses. Sampled fish were visually assessed prior to processing, and only healthy-looking individuals were selected for tagging. The total length (TL), and weight (smolts only) of the fish was recorded, and total handling time was around two minutes per fish. In Aurland, Lærdal, Årdal and Fortun fish were tagged close to the riverbank (10 – 40 m) and allowed to recover in aerated tanks, before being released to their river of origin after a period of 2 – 3 h for smolts, and 15 – 20 min for veteran migrants. Smolts from Mørkrid were transported between the tagging site and the release site by car (5 – 7 min) in recovery tanks (40 L, water flow of 5 – 7 L min^−1^). Fish were not handled directly post tagging. The surgical procedure was conducted according to [57, 58], approval was granted by the Norwegian Animal Research Authority (ID 4638).

Five different models of acoustic tags were deployed over the course of the study (LP-7.3: 7.3 × 1.8 mm, 1.8 g; LP-9: 9 × 24 mm, 4 g; MP-9-SHORT: 9 × 24.4 mm, 3.6 g; AST-9-LONG 9 × 29.4 mm, 5.2 g and ADT-13-STAT: 12.7 × 33.3 mm, 7.1 g; Thelma Biotel AS, Trondheim, Norway). Each model varied in dimension, mass, battery life, transmission rate and transmission power. All tag models transmitted coded 69 kHz signals with a unique ID code, larger tags transmitted depth and temperature information in addition (see Table S1 for an overview of tag specifications, dimensions and weight). Smolts were tagged with LP-7.3 tags (power output: 139 dB at 1 m depth), these tags transmitted an individual ID code, with transmission rate and battery expectancy ranging from 30 – 240 s and 6 – 11 months, respectively. The mean tag-weight burden for tagged smolts was 3.6% (range: 1.2 – 10.8%) of body weight. Veteran migrants were tagged with ID-only transmitters (LP-9, power output: 142 dB, MP-9-SHORT, power output: 146 dB), and depth and temperature data transmitters (AST-9-LONG, power output: 146 dB, ADT-13-STAT, power output: 153 dB). Transmission rate and battery expectancy of the ID-only tags ranged from 30 – 240 s and 6 – 20 months, and 90 – 360 s and 15 – 31 months for temperature / depth tags. Fish weight was not measured during veteran migrant sampling thus, tag burden cannot be calculated, however total fish length (TL) ranged from 24 – 69 cm (mean ± SD; 38.9 cm ± 10.9, *N* = 80) for fish tagged with ID-only tags, and 22 – 86 cm (43.9 cm ± 12.2, *N* = 275) for temperature/depth tags. After implantation, the functionality of the tags was tested by placing an acoustic receiver within the recovery tank. A total of 85, 19, 50, 50 and 15 smolts were tagged in Aurland, Lærdal, Årdal, Fortun and Mørkrid respectively, and a total of 89, 120, 77 and 69 veteran migrant brown trout were tagged in the rivers Aurland, Lærdal, Årdal and Fortun. No veteran migrants were tagged in the river Mørkrid (Table 1). Where body mass measurements were available (smolts only, *N* = 147, 67%), individual Fulton’s condition factor was calculated by applying the formula:$K=100 W {TL}^{-3}$, where W is mass (g) (TL cm), where K is an indicator of the individual’s energetic state [59]. A statistically significant correlation between TL and K was not observed (*R*^*2*^ = 0.23, *p* > 0.05), and therefore treated as independent variables (Table 1). For veteran migrant brown trout, size-specific estimates of fecundity (${Fec}_{TL}$) were generated according to ${Fec}_{TL}={e}^{-4.03+2.74* TL}$ (based on data in [60]).

Individual age, smolt length and growth rate was back calculated from scale annuli. By assuming proportional growth of scales and fish body length, the age specific length for each year at sea could be back calculated from ${TL}_{n}=\left(\frac{{S}_{n}}{S}\right)*TL$ where ${TL}_{n}$ and *TL* are length of fish at age *n* and at capture, respectively and *S*_*n*_ and *S* are scale radii at age *n* and total scale radii, respectively [61, 62] (Table 2). From annual ${TL}_{n}$ specific growth rates could be estimated as ${g}_{n}=\mathrm{ln}\left({TL}_{n+1}\right)-\mathrm{ln}({TL}_{n})$. For comparisons between time periods (historical / contemporary), marine migration distance (as fjord zone, see Fig. 1) and populations, ANOVAs were applied to explore these effects on sea-age specific growth rates (${g}_{SW}$).

**Table 2:** Overview of historical and contemporary Sognefjord veteran migrant brown trout scale samples, from which the 1^st^ / 2^nd^ sea age specific growth (${g}_{SW1}$ and ${g}_{SW2}$_,_ respectively) were estimated. The number of tagged fish, for which marine migration distance and behaviour data was collected (derived by acoustic telemetry) is also stated

River:	Aurland	Lærdal		Årdal	Fortun
Sampling period: (years)	Present 2012—2013	Past 1952—1970	Present 2009—2014	Present 2013	Present 2013
N sampled: (Mean TL, range)	24 (44.6, 32—72)	195 (55.5, 30—94)	140 (47.9, 22—92)	17 (41.0, 34—52)	15 (52.0, 29—81)
N fish tagged: (Mean TL, range)	24 (44.6, 32—72)	NA	84 (42.9, 22—74)	17 (41.0, 34—52)	15 (52.0, 29—81)
${g}_{SW1}$: Mean ± SD (N)	0.580 ± 0.146 (23)	0.675 ± 0.170 (193)	0.703 ± 0.172 (115)	0.702 ± 0.142 (17)	0.696 ± 0.134 (14)
${g}_{SW2}$: Mean ± SD (N)	0.408 ± 0.093 (12)	0.394 ± 0.110 (169)	0.380 ± 0.132 (63)	0.413 ± 0.169 (3)	0.442 ± 0.179 (8)

### Fish sampling- historical samples

**Fig. 2:**
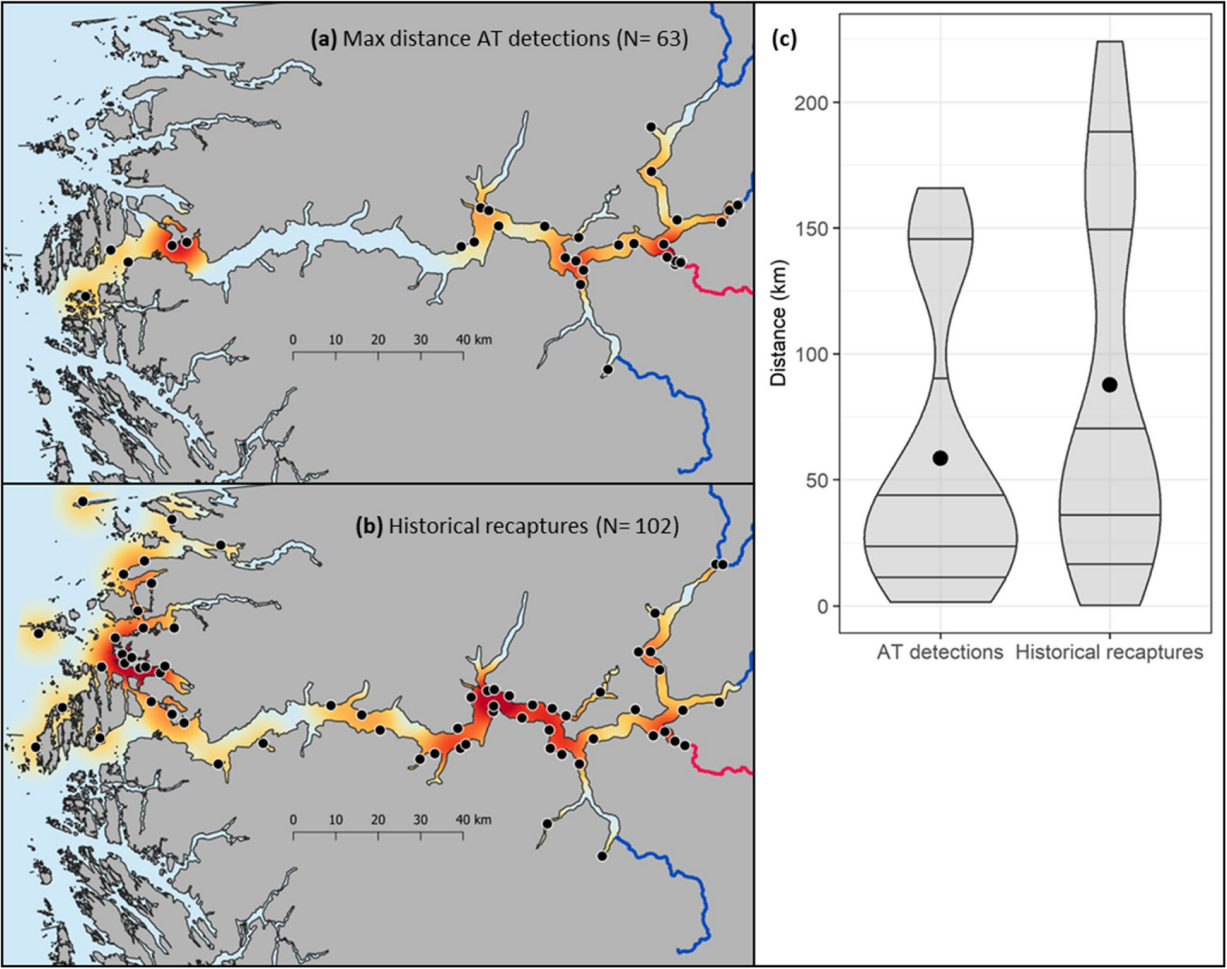
Heat-map presenting the location (black dots) frequency of (**a**) contemporary maximum marine migration distance of veteran migrant brown trout tagged with acoustic transmitters from the river Lærdal (coloured red) during the period 2012 – 2014 (*N* = 63, heat scale = 0 – 11) and (**b**) historical recapture locations (1950 – 1965, *N* = 102, heat scale = 0 – 4) of carlin-marked veteran migrant brown trout originating from the river Lærdal. **c** Violin plot showing the waterway distances of the acoustic telemetry derived maximum seaward migration extent and historical recapture positions (derived from provided location names) of Lærdal veteran migrant brown trout. The maps were produced by the authors

A total of 295 veteran migrant brown trout (mean, range TL: 64.4, 27 – 106 cm) were marked with carlin tags in the river Lærdal between 1950 – 1965 [51]. Of these 102 (TL: 64.4, 27 – 92 cm) individuals were recaptured by local fishermen throughout Sognefjorden, providing historical recapture locations (Fig. 2b). In addition, scales were sampled from 195 Lærdal veteran migrant brown trout (TL: 55.5, 30 – 94 cm) during the period 1956 – 1970, from which historical sea growth rates were back-calculated from the scale readings (Table 2).

### Acoustic receiver network

A network of 138 receivers (VR2W; InnovaSea Systems Inc. Boston, U.S.) were deployed throughout the study period (September 2012 – April 2016) (Fig. 1). The receiver network was designed to span the total migration habitat from in-river to open sea. Detection occurs when tagged fish swim within the transmission range of a receiver. The date, time and unique identity of the fish are then transmitted and stored onto the detecting receiver, as well as depth and temperature data if applicable. Receiver locations were selected to maximise detection probability of migrating tagged fish, with receivers set on adjacent sides of the fjord creating ‘gates’ or placed where the fjord is narrowest. Transmission range of the tags is dependent on environment and tag power, ranging from a few meters within rivers and up to one km in the fjord. The receiver network was downloaded approximately every three months to ensure continuous operation and prevent loss of data.

### Data analyses

#### Acoustic telemetry data

The telemetry data was filtered by inspection of individual fish tracks to identify erroneous or false detections (Figure S1, S2). Detections were deemed ‘false’ and removed if a) they were at an improbable position in time and space relative to preceding detections, or b) they occurred post expected battery expiration for a given tag. Watercourse distances were estimated from receiver positions and historical recapture positions (derived from provided location names) as end points and river outlet positions as start points using shortest-path analysis in QGIS. This was done by constructing a fjord midline network (i.e., a line vector shp-file) that covered all fjord branches from the five river mouths of interest to the extent of the recapture and receiver positions. To aid spatial analyses of the telemetry data, receivers were assigned to a habitat zone based on their geographical location within the study system: freshwater (F), inner-fjord (I), mid-fjord (M) and outer-fjord (O) (Fig. 1). To facilitate temporal analyses of the telemetry data, annual detections was assigned to a season accordingly: winter-late = week of year (WoY) 1 – 12, spring/summer = WoY 13 – 26, autumn = WoY 27 – 40, winter-early = WoY 41 – 52. Individuals were defined as migrants if they were detected within the fjord (I/M/O). Migration onset date was defined as the date and time of the first detection at a receiver in zone I. Annual residence duration (weeks) was calculated by determining the arrival and departure time within each habitat zone during each season. Where multiple visits occurred, values were summed to produce a total value of residence duration within each given habitat zone and season, for each year of detection data.

#### Modelling of brown trout habitat use and migratory behaviour

Maximum marine migration distance of each fish was determined from the most seaward habitat zone, derived as the maximum watercourse distance (km) from the mouth of origin river and modelled using linear models (LM) with river (origin of tagged fish), TL and K as predictors (Table S2). To prevent violations of homoscedasticity a log-transformation was applied to the response variable.

**Fig. 3:**
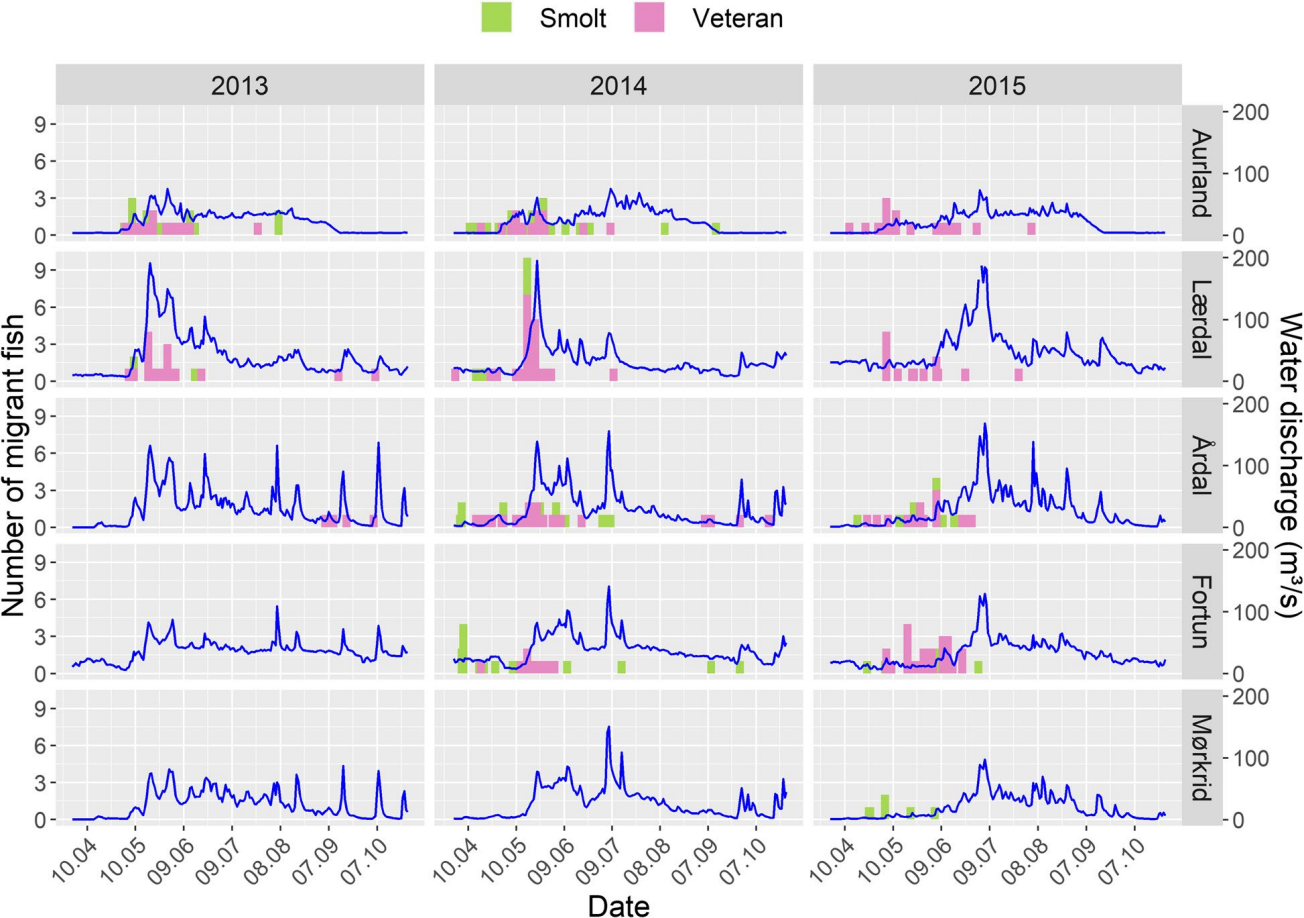
Water discharge (m^3^/s) of the five study rivers for the period 31/03 – 27/10 (DoY: 90 – 300) for the three years sampled (2013 – 2015). Count (left panel) and date of migration onset of tagged Sognefjord brown trout smolts (*N* = 92) and veteran migrants (*N* = 227) are presented, where missing count data is due to an absence of sampling in a given river and year

Generalised linear modelling (GLM) was used to model the migration probability across rivers given the river-specific discharge conditions on each day [63, 64]. The response was modelled as a binomial distribution defined by whether an individual migrated on a given day within a given river. The most complex candidate model included river, TL, daily mean standardised water discharge (stQ), and sequential change in daily mean standardised water discharge (ΔQ) as predictors (Table S2). Daily mean values of water discharge (m^3^s^−1^) were calculated for each river from data provided by the Norwegian Water Resources and Energy Directorate (NVE). Given the magnitude of variation in discharge among rivers (Fig. 3), these values were then scaled within all rivers to provide values of relative discharge (stQ: mean = 0, SD = 1). Day-to-day change in these values were then calculated for each river: $\Delta Q=({Q}_{t}- {Q}_{t-1})/{Q}_{t-1}$. The applied data was a subset, encompassing the period March 30 – July 13 (day of year (DoY) 90 – 195). For comparisons between migrant and resident immature fish, one-way ANOVAs were applied to explore if TL and K differed between these groups.

Individual residence duration was modelled using a set of linear mixed-effect models (LMM) with river, TL, season, habitat zone and max zone (the most seaward habitat zone reached during each period) as fixed effects, with fish ID included as a random intercept to account for repeated observations of individual fish [64] (Table S2). To prevent violations of homoscedasticity a log-transformation was applied to the response variable.

Water depth use by veteran migrants was modelled with a set of LMMs. The candidate predictors were river, TL, season and habitat zone as fixed effects and fish ID as a random intercept effect. Depth use greater than 50 m was excluded from the analysis (*N* = 2384, 5.26%) as it was impossible to differentiate real depth values with faulty depth codes at this depth.

For each response variable, models were run independently for smolt and veteran migrant brown trout. The relative model support in the data was assessed using Akaike information criterion (AIC)[63], adjusted for small sample size (AIC_c_) using the R-package AICcmodavg [65] (Table S2). Backwards selection was undertaken to remove non-significant interaction effects in selected models [64], to extend the principle of model parsimony. Data analysis and modelling was conducted using R software version 4.2.0 [66].

#### Mark-recapture modelling

To overcome problems associated with partial data analysis, derived from imperfect detection rate and the resulting unknown fate of non-recaptured (detected) individuals, a spatial, multi-state mark-recapture model structure (conditional Arnason-Schwarz (CAS)) [67, 68] was used to simultaneously estimate encounter-, survival- and movement probabilities of tagged fish. Four states (the habitat zones: F/I/M/O) were defined to represent transition/movement between fjord zones in an outward (F to O) and inward (O to F) direction. A temporal capture history was constructed for each smolt (*N* = 175), formed of 14 encounter occasions corresponding to bi-weekly intervals over a 6-month period (WoY: 13 – 40). For each veteran migrant (*N* = 250), a capture history comprising of 47 occasions (WoY: 39 – 12, 2012 – 2016), composed of bi-weekly encounter occasions during spring/summer (WoY: 13 – 26, 14 weeks) and autumn (WoY: 27 – 40, 14 weeks) and a single encounter occasion of 24 weeks representing winter (WoY: 41 – 12) was constructed. These two-week time periods proved to be a good compromise that both allowed for sufficient detection time for within-zone detections and also providing sufficient time resolution to capture temporal processes of among-zone transitions and survival, and, finally, satisfying computational constraints [69]. Initial attempts of weekly time resolution proved unsuccessful as few week-constrained estimates of the key parameters were possible to estimate. Where expected battery life of the tags expired prior to the last time occasion these capture histories were right censored (*N* = 89, veteran migrants only). Individuals were assigned to the maximum zone in which they were detected for a given occasion. Models were built independently for smolt and veteran migrant brown trout.

Using the programme MARK, version 6.2 [70] maximum likelihood parameter estimates of candidate CAS models were obtained for ${p}_{t}^{j}$ = the probability that an individual was detected in zone *j* during time *t* given that the individual was alive, ${S}_{t}^{j}$ = the probability that an individual survived in zone *j* between time *t* and *t* + 1 and did not permanently emigrate from the study area, and ${\psi }_{t}^{j-k}$ = the probability that an individual in zone *j* at time *t* transitions to zone *k* at time* t* + 1 given that the individual survived until *t* + 1. Thus, the most complex model included separate probability (*p*), survival (*S*), and transition (*ψ*) parameters for each zone, river, season, year, and sampling occasion (*t*). The effects of tag power on detection probability and fish length (standardised TL) on survival were also included as continuous covariates in the models, however problems with model convergence often precluded our ability to test interactions of these individual covariates. To extend the principle of model parsimony, candidate models were built using forward selection and the final models were selected using AICc [71] (Table S6, S8). Goodness-of-fit testing was conducted on a fully parametrized, simplified model (${p}_{t}^{Z}$, ${S}_{t}^{Z}$, ${\psi }_{t}^{Zj-Zk}$) using U-CARE version 3.3.0 [72].

#### Simulated trajectories of anadromy

By combining the chosen CAS models, migration onset model, residence duration model (veteran migrants), residence time distribution data per zone and river (smolts) and maximum migration distance models, simulations of individual fjord-use trajectories and fates were run using the 2013 – 2015 conditions (veteran migrants) as environmental frames. Separate simulations were run for smolts and veteran migrants. The simulations were run using a weekly time resolution following 1000 individuals per river, per year as initial populations. The smolt simulations were run for a 6-month period (WoY: 13 – 40) and the veteran simulations for the entire year. The initial 1000 individuals were assigned trait values for total length* (TL*_*i*_) and Fulton’s condition factor* (K*_*i*_) randomly drawn from river-specific trait summary statistics (i.e., ${TL}_{i}=rnorm({\mu }_{TL}^{R},{\sigma }_{TL}^{R}$), ${K}_{i}=rnorm({\mu }_{K}^{R},{\sigma }_{K}^{R}$)), where µ = mean and σ = SD), with simulations following each individual from week to week. Individual marine migration distance was derived from the corresponding smolt and veteran migrant models (Table S3), according to individually assigned trait values. Individual sea growth of veteran migrants during the simulated period was estimated from the back-calculated 2^nd^ sea age specific growth (${g}_{SW2}$) (Table 2), where discrete values of ${g}_{SW2}$ were assigned according to the maximum migration distance of the individual, as the most seaward fjord zone (maxZ) (${TL}_{rep}={TLe}^{{g}_{SW2(maxZ)}* 0.5}$). For veteran migrants, ${g}_{SW2}$ is the most relevant available measure of sea growth rate as it corresponds to the growth rate experienced during an anadromous individual’s second year (i.e., as a veteran) at sea. Weekly* (t*) survival probability was estimated using the survival estimates* (S*_*t*_) derived by the CAS models by drawing a fate of either survival or death using the rbinom function in R (*rbinom(S*_*t*_)). Given a survival draw, weekly between-zone transitions (${\psi }_{t}^{jk}$*, j* = exit zone, *k* = entrance zone) were estimated from the CAS model, except for freshwater-to-inner fjord transition (${\psi }^{FI}$) during the spring period (i.e., WoY 13 – 26) for veteran migrants, where the river discharge- and individual characteristic-driven migration onset model was used (Table S3). Technically, the weekly between-zone transitions (also including the probability to stay) were derived from a multinominal draw from the relevant river- and period-specific transition matrix (${\mathrm{M}}_{jk}$) generated from the CAS models using the rMultinom function in R (rMultinom(${\mathrm{M}}_{jkt}$)). For veteran migrants, estimates of residence duration within each zone were derived from a fitted residence duration linear model (Table S3). For smolts, we found no model for residence duration with predictive power higher than 10%. Therefore, residence duration values were calculated from the mean accumulated duration, dependent upon the maximum migration distance, for each river (Table S4). To prevent over-use of zone- and period-specific residence times, the time spent in each zone at each time step was chronicled, and from this the fraction of spent residence time per zone was calculated and used to modify the transition matrixes. We used two rules of operation for this modification; one ‘pushing’ procedure that weighted in favour of the upper off-diagonal of the ${\mathrm{M}}_{jk}$ matrix to push the individual towards the outer-fjord, and a ‘pulling’ procedure that weighted in favour of the lower off-diagonal of the ${\mathrm{M}}_{jk}$ matrix in order to pull the individual back towards freshwater. The first ‘pushing’ procedure was applied during the spring/summer period, and the latter during the autumn period. In total, the simulations resulted in 1000 individual zone-use trajectories per river, year and life-stage (smolts and veteran migrants) with each simulation iterated 100 times. All simulations were produced in R version 4.2.0 [66].

#### Simulated expression of anadromy, survival and relative contribution to fecundity

From each simulated iteration, the maximum distance (as fjord zone) selected per individual, river and year was extracted from the initial population of 1000 fish $({N}_{Start}$). The number of surviving individuals (${N}_{Surv}$) was then calculated from the number of survivors per population, per year at the end of each run. The maximum zone-specific survival rate (${S}_{maxZ}$) was estimated from the fraction surviving dependent upon selection of maximum marine migration distance, per year at the end of each run (${S}_{maxZ}= \frac{{N}_{Surv}}{{N}_{Start}}$). For the smolts, values of ${S}_{maxZ}$ were used as a proxy for fitness, but for veteran migrants realised individual fecundity was estimated from the product of mean expected individual fecundity ($\overline{{Fec }_{T{L}_{Rep}}}$) and ${S}_{maxZ}$, and realised population fecundity was estimated from the product of total mean expected individual fecundity ($\Sigma \overline{{Fec }_{T{L}_{Rep}}}$) and ${N}_{Surv}$. Where only individuals expected to contribute to the spawning population (${TL}_{Rep}$ > 35 cm, alive and retuned to freshwater during the period WoY 37 – 52) were included in the estimates of realised fecundity.

## Results

### Historical versus contemporary migration distance and growth

After filtering of the telemetry data, a total of 1,158,665 smolt and 7,613,819 veteran migrant detections remained. In total, 89.9% (175) of the tagged smolts were detected, of which 57.1% (100) were detected on fjord receivers (Table 1). The proportion of veteran migrants detected was 96.3% (342), of which 59.9% (205) were detected in the fjord. Most detections occurred in the inner-fjord, 97% of both smolts (*N* = 97) and veteran migrants (*N* = 195) were detected in this zone. In the mid-fjord 45% (*N* = 45) of smolts and 61% (*N* = 125) of veteran migrants were detected. This was reduced to just 7% (*N* = 7) and 19% (*N* = 38) of smolts and veteran migrants being detected in the outer-fjord, respectively.

A large variation in marine migration distance, both within and among populations was documented. The mean (± SD) maximum seaward extent of present-day migration was 41.8 ± 38.9 km (Aurland = 36.6, Lærdal = 52.0, Årdal = 36.2, Fortun = 45.5 and Mørkrid = 59.2) and 58.1 ± 50.8 km (Aurland = 70.7, Lærdal = 58.6, Årdal = 46.7 and Fortun = 56.3) for smolts and veteran migrants, respectively. Historical recapture locations of Lærdal brown trout (*N* = 102) were located at a greater distance (33.2%) from the river mouth (87.7 ± 70.3 km), when compared to the maximum migration distances of their present-day counterparts (58.6 ± 54.9 km) (Fig. 2). Both life-stages of brown trout were detected on the outer-most receivers with a maximum marine migration distance of up to 209 km (see Table 1), albeit by few individuals (2 smolts, 5 veteran migrants). A total of 17 individuals from the historical fjord recapture data were caught at a greater distance than the outer-most receiver (> 167 km, max distance 224 km).

No significant difference in the estimates of sea specific growth were observed between past and present Lærdal scale samples, but a significant difference in present-day 1^st^ year sea age specific growth (${g}_{SW1}$) was observed among populations (one-way ANOVA: F = 3.698, *p* = 0.0139) (Table 2, Figure S3). In veteran migrant brown trout for which both telemetry and growth data were collected (*N* = 117 and 47, ${g}_{SW1}$ and ${g}_{SW2}$_,_ respectively), greater sea specific growth was observed for individuals reaching the mid- and outer-fjord regions (${g}_{SW1}$ median ± SD: 0.65 ± 0.17, 0.66 ± 0.16, 0.72 ± 0.15 and ${g}_{SW2}$: 0.35 ± 0.16, 0.39 ± 0.12, 0.37 ± 0.14 of veteran migrant brown trout reaching the inner- mid and outer-zones respectively), although this trend was non-significant (Figure S3).

### Modelling of brown trout habitat use and migratory behaviour

**Fig. 4:**
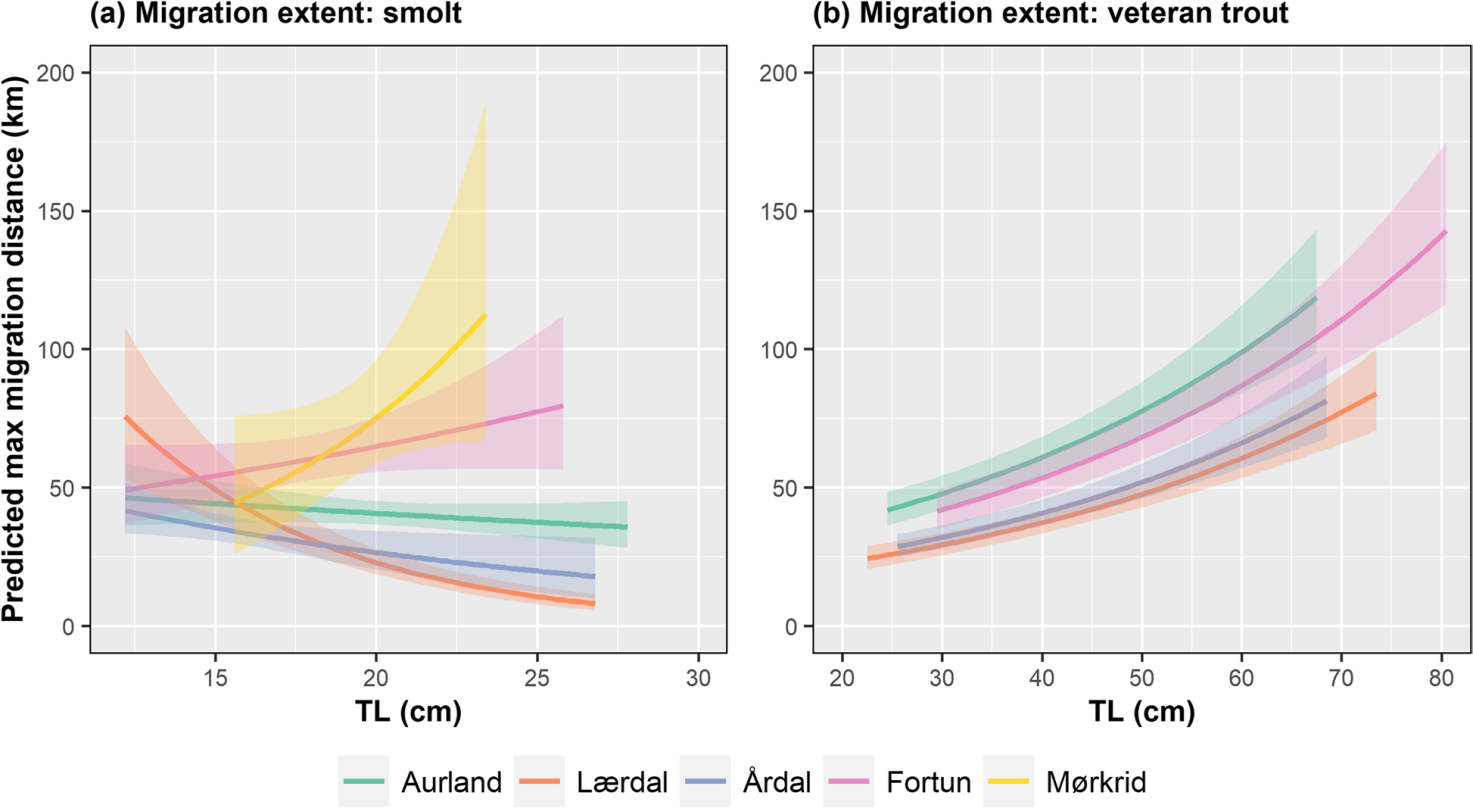
The effect of natal river and fish length (TL) on predicted maximum seaward migration distance (distance from river mouth) of Sognefjord brown trout smolts (*migration distance* ~ river*TL) (**a**) and veteran migrants (*migration distance* ~ *river* + *TL*) (**b**). Note: Models were selected according to AICc; candidate models are listed in Table S2. Model coefficients are stated in Table S3

Both the predictors TL and river were retained in the best performing models of marine migration distance for both smolts and veteran migrants (Table S2). For smolts an interaction term was included in the selected model with migration distance increasing with TL of smolts from Fortun and Mørkrid. Conversely migration distance decreased with increasing TL in smolts originating from Aurland, Lærdal and Årdal (Fig. 4a, Table S3). A simpler additive model was selected for veteran migrants (Fig. 4b, Table S3), scoring 1.44 AICc values lower than the second-ranked interaction model (river*TL) (Table S2). The predictor K was included in candidate LMs on a subset of the smolt data (*N* = 66), however limited support for these models was observed, therefore this approach was dropped in favour for the complete data set.

**Fig. 5:**
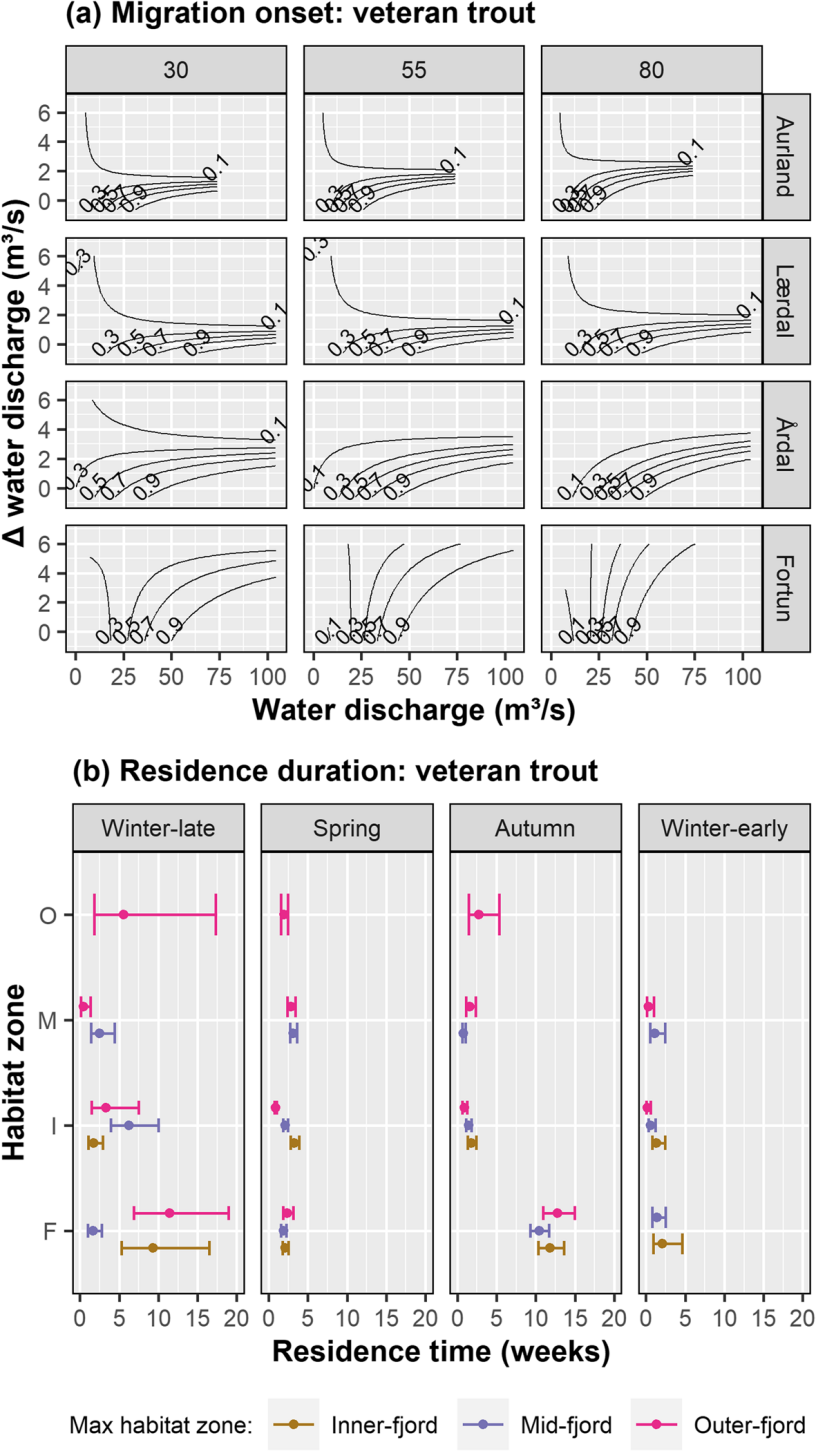
Contour plots show probability of veteran migrant brown trout migration onset (**a**) for a given TL (30, 55 and 80 cm, top panel) for each study river (right panel). Predictions show the effect of water discharge and daily change in water discharge (*probability of migration onset* ~ stQ*ΔQ*river*TL). Plot (**b**) depicts predicted annual residence duration of veteran migrant brown trout within a given habitat zone (F = freshwater, I = inner-fjord, M = mid-fjord, O = outer-fjord) of Sognefjorden (*residence duration* ~ season*maxZ*zone). Note: Models were selected according to AICc; candidate models are listed in Table S2. Model coefficients are stated in Table S3. Values of predicted residence times are shaded according to maximum migration distance as a given habitat zone (Inner-, Mid-, Outer-fjord). Annual data was assigned to a season accordingly: winter-late = WoY 1 – 12, spring = WoY 13 – 26, autumn = WoY 27 – 40, winter-early = WoY 41 – 52. Error bars depict 95% CI

Median fjord entry dates were DoY: 138 and 142 (May 19 and May 23) for smolts and veteran migrants respectively (Fig. 3). Limited support for the best performing GLM fitted to the smolt data was observed, thus this approach was rejected for these data. Model selection for the veteran migrant data yielded a complex GLM model structure, however the number of parameters was reduced, as a result of backwards-selection (N parameters = 21). The final model included the variables stQ, ΔQ, River, TL and their interactions, and scored 111 AICc values lower than the simpler (N parameters = 17) second-most supported model (Table S2). The model revealed an interaction effect on migration probability, with increasing water discharge increasing the likelihood of migration onset in all rivers, with migration onset most probable during periods of consistently high discharge (i.e., low values of ΔQ) (Fig. 5a). In the river Fortun however, migration probability was less affected by ΔQ, especially in larger fish. Predicted mean values of migration onset probability were 0.41, 0.50, 0.87 and 0.53 for the rivers Aurland, Lærdal, Årdal and Fortun respectively (Fig. 7). For immature brown trout, migrating individuals were significantly larger (TL: 1 df, F = 8.87, *p* = 0.004) and in poorer condition (K: 1 df, F = 5.50, *p* = 0.021) than the fish that remained in freshwater (Figure S4).

**Fig. 6:**
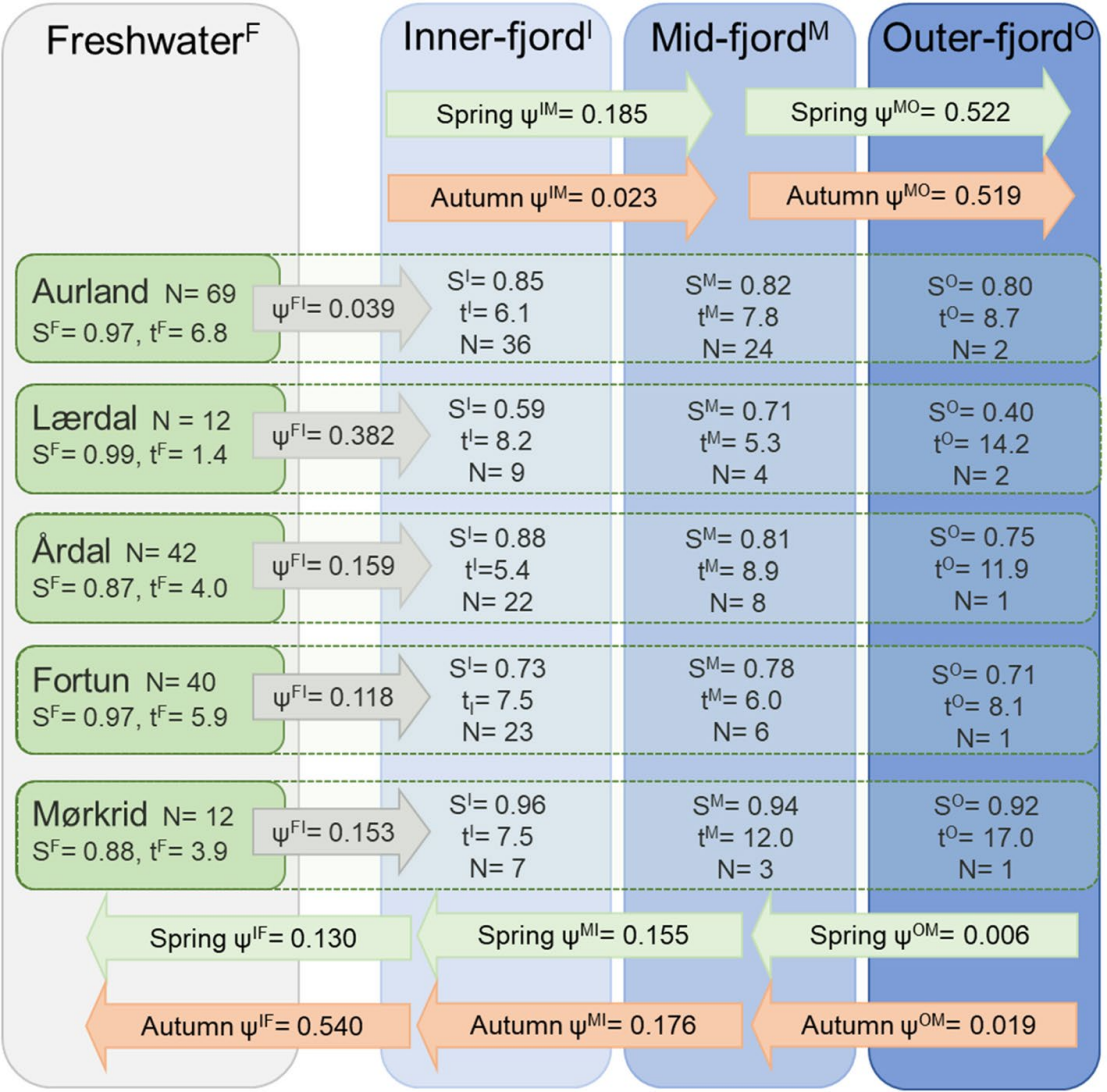
A conceptual overview of the migration dynamics of Sognefjord brown trout smolts (*N* = 175), derived from the conditional Arnason-Schwarz (CAS) model described in Table 3a. Shaded regions represent each habitat zone (F, I, M, O), dashed lines separate values for each natal river. Estimates of survival probability (S) are weighted as an exponent of observed residence duration (t, weeks) within each habitat zone. Arrows indicate the direction (inwards/outwards) and timing (spring/summer, autumn) of migration probability between zones (ψ). Note: Fjord residence is calculated as the mean accumulated duration of all migrant smolts (*N* = 100) dependent upon maximum migration distance (habitat zone), for each river. Freshwater residence is calculated as the mean accumulated duration of all migrant fish in freshwater, per river (Table S4). Transition probabilities (ψ) were estimated according to season and direction, except ψ^FI^ which is estimated according to natal river. Seasons were defined accordingly: spring/summer: WoY = 13 – 26, autumn: WoY = 27 – 40. Combined transition probabilities e.g., transition to the outer-fjord includes ψ.^XO^ where *X* includes F, I and M, to ease complexity in illustration. For specific transition estimates see Tables 3a and S7

**Fig. 7:**
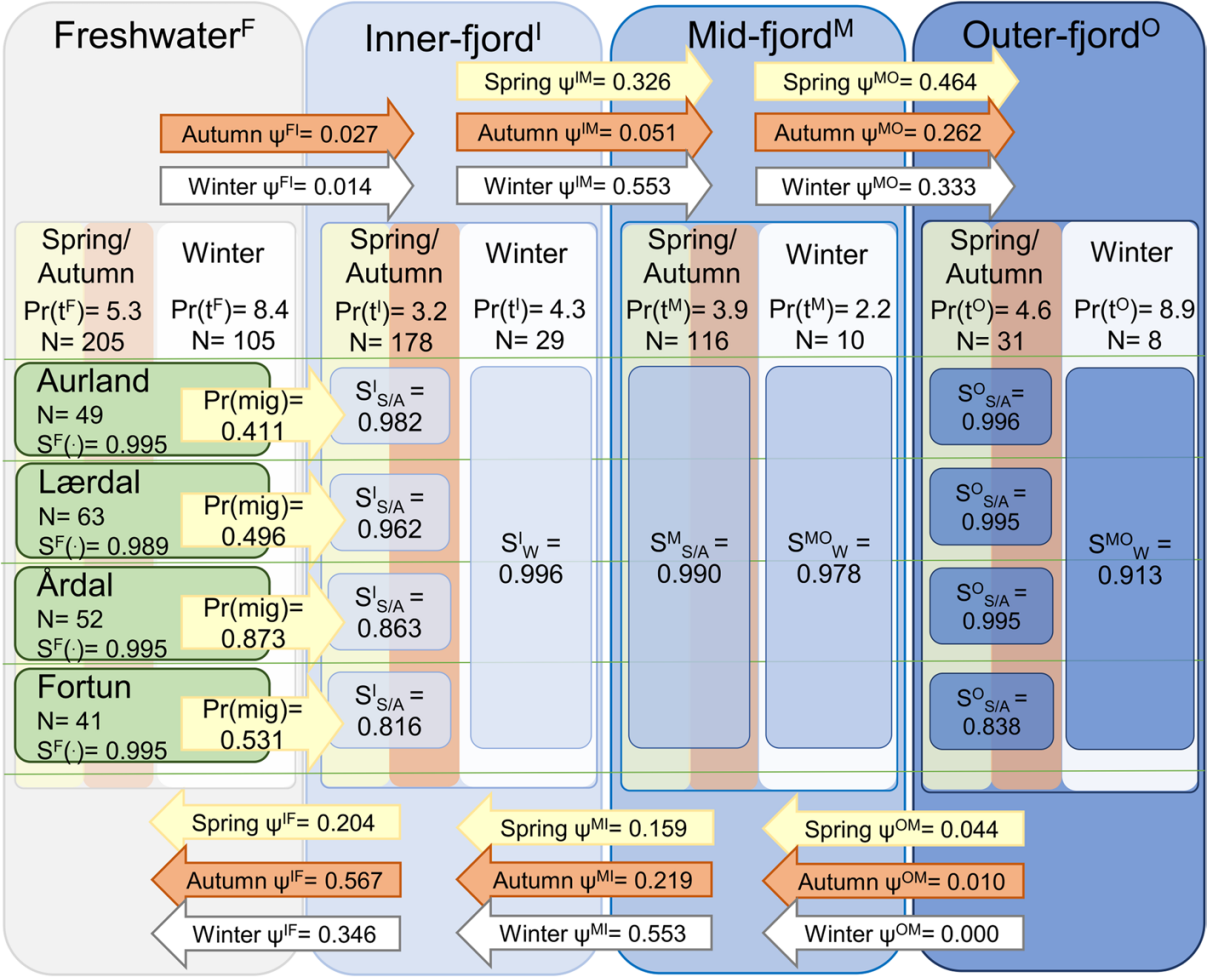
A conceptual overview of the migration dynamics of Sognefjord veteran migrant brown trout (*N* = 250), derived from the conditional Arnason-Schwarz (CAS) model described in Table 3b. Shaded regions represent each habitat zone (F, I, M, O), within which values are divided by season (spring–summer/autumn and winter, denoted by yellow/orange and grey shading) and river (green shading). Estimates of survival probability (S) are weighted as an exponent of predicted residence duration (Pr(t), weeks). Arrows indicate the direction (inwards/outwards) and timing (spring–summer/autumn, winter) of migration probability between zones (ψ). Note: Predicted values of t were extracted from the LM (Fig. 5b, Table S3) for each habitat zone and season with values for spring–summer/autumn and winter-early/winter-late summed, corresponding to the seasonal estimates of *S*. Spring migration onset probabilities (Pr(mig)) were extracted from the GLM (Fig. 5a, Table S3) for an individual brown trout of mean TL, at a mean value of stQ and ΔQ for each river. Seasons were defined accordingly: spring/summer: WoY = 13 – 26, autumn: WoY = 27 – 40, winter: WoY = 1 – 12 and 41 – 52. Combined transition probabilities e.g., transition to outer-fjord includes ψ^XO^ where *X* includes F, I and M, to ease complexity in illustration. For specific transition estimates see Tables 3b & S9. “(^.^)” indicates that predictors were held constant, i.e., no seasonal effect on S in freshwater

Fjord residence of smolts was calculated as the mean accumulated duration of all migrant smolts (*N* = 100), dependant on the maximum zone reached for each population. This alternate approach was adopted due to poor performance of the residence duration models fitted to the smolt data, with variation in residence duration data observed among individuals, populations, and habitat zones (Figure S5, Table S4). Lærdal smolts resided in freshwater the least (mean weeks ± SD: 1.62 ± 1.3, *N* = 8) and Fortun the most (7.22 ± 5.5, *N* = 23). Conversely, Lærdal smolts used the outer-fjord zone the most (6.25 ± 1.6, *N* = 2) and Fortun smolts the least (0.31 ± NA, *N* = 1). The mean residence duration of tagged migrant smolts was 5.09 (*N* = 96), 5.09 (*N* = 97), 4.23 (*N* = 45) and 2.40 (*N* = 7) weeks in the freshwater, inner-, mid- and outer-fjord zones respectively (Fig. 6, Table S4). For the veteran migrant brown trout, variation in the data was explained by the residual variance term alone (variance = 0.00 (Fish ID), 1.611 (residual)), thus a simplified LM approach was deemed appropriate. The selected model included the variables season, max zone, habitat zone and their interactions (N parameters = 35), despite limited data in the outer-fjord creating rank-deficiencies in the model (Fig. 5b, Table S3). No effect of fish length (TL) or river was observed, with a ΔAICc score for the best performing models including these parameters being 2.15 and 10.39 respectively (Table S2). The model predicted that veteran migrants remained in the fjord for longer periods if they utilised the outer-fjord zone, with annual fjord residence predicted as 20.7 ± 4.5, 17.9 ± 3.6, and 8.2 ± 0.8, weeks for fish that reached the outer-, mid- and inner-fjord respectively. Seasonal variation in residence duration was most prominent in freshwater with predicted mean occupancy being 11.7 ± 1.18 weeks in autumn and just 2.2 ± 0.25 in spring/summer. Predicted mean residence duration in each habitat zone was 6.1 ± 4.90, 2.0 ± 1.66, 1.6 ± 1.10 and 3.4 ± 1.88 weeks for freshwater, inner-, mid- and outer-fjord zones respectively (Figs. 5b and 7).

Due to limited data and a high level of individual variation, modelling the water depth of veteran migrants was inappropriate. Mean fish depth of 114 veteran migrants was 13.3 m ± 13.0, with fish residing at greater depth during winter (21.7 ± 11.6) than during spring/summer (9.4 ± 12.3) and autumn (8.1 ± 9.8). Årdal brown trout utilised greater depths (24.8 ± 12.7) than fish from the remaining rivers (Aurland = 5.1 ± 8.9, Lærdal = 9.3 ± 9.3 and Fortun = 3.4 ± 4.5) (Figure S6, Table S5).

### Mark-recapture modelling

Goodness-of-fit testing revealed the smolt model fitted the data (GOF test: χ^2^ = 94.59, *df* = 74, *p* = 0.054), but the veteran migrant model had variance inflation issues under the fitted model structure (χ^2^ = 278.65, *df* = 192, *p* < 0.01). We therefore applied an estimate of variance inflation to the models (smolt model: ĉ = 1.27, veteran model: ĉ = 1.45) to compensate for lack of fit during model selection. This resulted in a reversal in the ranking of the top two performing veteran models, however only 0.8 QAICc units separated the two, thus the original model was upheld (Table S8). No change in the order of the top three ranked smolt models was observed, with the second-ranked model attaining 7.2 QAICc units higher than the selected model (Table S6). We were able to constrain the models (N parameters = 73 and 61 for smolt and veteran models, respectively) to obtain parsimonious estimates of detection probability (*p*), survival (*S*), and transition (*ψ*) (Table 3, S7, S9). A conceptual aid to facilitate understanding of the model outcomes are presented for both smolts (Fig. 6) and veteran migrants (Fig. 7). The figures combine the estimates of *S* and *ψ* generated by the CAS models, as well as the predicted values of residence duration and migration onset for veteran migrants (Fig. 5), and the residence time distribution data per zone and river for smolts (Table S4).

**Table 3:** (a) and (b) Logit parameter estimates for the selected conditional Arnason-Schwarz (CAS) mark-recapture model to estimate rates of survival (S) and transition (migration) (ψ) of tagged brown trout smolts (a) and veteran migrants (b) within Sognefjorden

**(a)** ***Salmo trutta*** **smolts (N par = 73)**									
**Survival (S)**					**Transition (ψ)**				
**Zone**	**River**	**Season**	**Estimate**	**SE**	**Zone**	**River**	**Season**	**Estimate**	**SE**
F	Fortun	All (^.^)	1.571	0.297	FI	Fortun	All (^.^)	4.012	0.088
I + M + O	Fortun	All (^.^)	1.164	0.086	FI	Mørkrid	All (^.^)	-2.374	0.173
F	Mørkrid	All (^.^)	1.218	0.154	FI	Årdal	All (^.^)	-0.750	0.118
I + M + O	Mørkrid	All (^.^)	1.428	0.346	FI	Lærdal	All (^.^)	-2.903	0.251
F	Årdal	All (^.^)	1.194	0.095	FI	Aurland	All (^.^)	-1.171	0.057
I + M + O	Årdal	All (^.^)	1.261	0.082	FM	All (^.^)	S	-1.861	0.057
F	Lærdal	All (^.^)	7.854	0.311	FM	All (^.^)	A	-1.447	0.073
I + M + O	Lærdal	All (^.^)	1.065	0.120	FO + IO	All (^.^)	S	5.454	0.064
F	Aurland	All (^.^)	1.571	0.077	FO + IO	All (^.^)	A	-0.938	0.079
I + M + O	Aurland	All (^.^)	1.250	0.082	IF	All (^.^)	S	4.365	0.111
					IF	All (^.^)	A	-0.630	0.107
					IM	All (^.^)	S	-7.019	0.122
					IM	All (^.^)	A	-1.849	0.093
					MF	All (^.^)	S	-1.571	0.278
					MF	All (^.^)	A	-6.821	0.240
					MI	All (^.^)	S	4.236	0.187
					MI	All (^.^)	A	-2.165	0.234
					MO	All (^.^)	S	-0.503	0.243
					MO	All (^.^)	A	-9.067	0.242
					OF + OI	All (^.^)	S	4.070	0.065
					OF + OI	All (^.^)	A	5.324	0.082
					OM	All (^.^)	S	-1.420	0.084
					OM	All (^.^)	A	-1.292	0.090
**(b) Veteran migrant** ***Salmo trutta*** **(N par = 61)**									
**Survival (S)**					**Transition (ψ)**				
**Zone**	**River**	**Season**	**Estimate**	**SE**	**Zone**	**River**	**Season**	**Estimate**	**SE**
F	Aurland	All (^.^)	1.571	0.111	FI	All (^.^)	A	-1.243	0.032
F	Fortun	All (^.^)	1.571	0.120	FI	Aurland	S	-7.218	0.067
F	Lærdal	All (^.^)	1.662	0.024	FI	Fortun	S	-0.818	0.071
F	Årdal	All (^.^)	1.501	0.033	FI	Lærdal	S	-0.857	0.092
I	Aurland	S + A	1.722	0.165	FI	Årdal	S	-0.742	0.079
I	Fortun	S + A	1.072	0.071	FM	All (^.^)	A	-1.380	0.033
I	Lærdal	S + A	1.351	0.075	FM	All (^.^)	S	-1.052	0.038
I	Årdal	S + A	1.145	0.082	FO + OF	All (^.^)	A	4.168	0.032
M	All	S + A	1.511	0.179	FO + OF	All (^.^)	S	-0.864	0.042
O	Aurland	S + A	-4.712	0.244	IO + OI	All (^.^)	A	-14.371	0.041
O	Fortun	S + A	1.178	0.081	IO + OI	All (^.^)	S	-2.010	0.036
O	Lærdal	S + A	1.571	0.072	IF	All (^.^)	A	-8.909	0.096
O	Årdal	S + A	1.571	0.194	IF	All (^.^)	S	-2.023	0.074
I	All	W	1.571	0.081	IM	All (^.^)	A	-1.981	0.095
M + O	All	W	1.368	0.035	IM	All (^.^)	S	-0.500	0.071
					MF	All (^.^)	A	5.738	0.127
					MF	All (^.^)	S	4.341	0.072
					MI	All (^.^)	A	3.771	0.124
					MI	All (^.^)	S	-0.890	0.070
					MO	All (^.^)	A	18.144	0.131
					MO	All (^.^)	S	-6.701	0.075
					OM	All (^.^)	A	11.200	0.040
					OM	All (^.^)	S	-1.992	0.038
					FO + OF	All (^.^)	W	3.483	0.074
					FI + IF	All (^.^)	W	11.231	0.068
					FM + MF	All (^.^)	W	4.712	0.121
					IM + MI	All (^.^)	W	0.106	0.255
					IO + MO + OI + OM	All (^.^)	W	4.712	0.190

Estimates of detection probability (*p*) are not provided as they have been estimated under temporal variation (see Tables S7 & S9, for the complete list of model estimates), the complete number of model parameters in each model are stated above (N par). The models were selected according to AICc, candidate models are listed in Tables S6 & S8. SE denotes the standard error of the parameter estimates. Temporal effects were defined as: time, year and season (Tables S6 & S8). Seasons were defined accordingly, spring/summer (S) = WoY: 13 – 26, autumn (A) = WoY: 27 – 40 and winter (veteran migrants only) (W) WoY = 1 – 12 and 41 – 52. Four spatial states were defined as the habitat zones: *F* = freshwater, *I* = inner-fjord, *M* = mid-fjord, *O* = outer-fjord, and individuals were grouped by river of origin “ + ” indicates grouped as a single predictor and “(^.^)” indicates that predictors were held constant

The smolt data supported a time parameterisation of detection probability (*p*) for all habitat zones except the outer-fjord, thus 40 of the 73 model parameters were estimates of *p*. In the veteran migrant model, the number of detection probability parameters was halved (*N* = 20). The data supported a yearly (*N* = 4) estimate of *p* for each river in zone F, and a seasonal estimate in zone M. For both models estimates of *p* were largest in freshwater (*p*^*F*^ smolt = 0.852; veteran = 0.757) and smallest in the outer-fjord (*p*^*o*^: smolt = 0.021; veteran = 0.042).

The smolt model estimated a constant probability of fortnightly survival rate in the fjord for each river of origin (*S*^*IMO*^: Aurland = 0.975, Lærdal = 0.937, Årdal = 0.976, Fortun = 0.959, Mørkrid = 0.995), with a separate estimate generated for freshwater (*S*^*F*^: Aurland = 1.0, Lærdal = 1.0, Årdal = 0.965, Fortun = 1.0, Mørkrid = 0.969). For three of the rivers, estimates of *S*^*F*^ were greater than *S*^*IMO*^ (mean: *S*^*F*^ = 1.0, *S*^*IMO*^ = 0.957), however for Årdal and Mørkrid smolt populations survival in the fjord was estimated as 1.1 and 2.6% higher than in freshwater, respectively. In contrast to the smolt model, estimates of veteran migrant survival incorporated the temporal variable season in addition to spatial zone, with discrete estimates of *S* generated for spring/autumn (combined) and winter in the inner-, mid- and outer-fjord zones. Estimates of *S* within the fjord were greater during winter (*S*^*IMO*^ mean 0.995) than during spring/autumn (0.982). Additionally, discrete estimates were supported for each population during spring/autumn for the inner- and outer-fjord zones. Replicating the smolt model, estimates of *S*^*F*^ were also separated for each river, but no seasonal effect of survival was observed in this zone.

The models yielded estimates of *ψ* that varied by season and habitat zone. Thus, we were able to determine directional, seasonal probabilities of migration. In both models transition probabilities oscillated seasonally, with estimates of *ψ* in a seaward direction greater than *ψ* in an inward direction during spring/summer, with the pattern reversed during autumn. During winter (veteran migrants only) transition probabilities were equally high in both directions. Smolts originating from Lærdal were most likely to enter the fjord (${\psi }^{FI}$= 0.382 ± 0.122), ten times more likely than fish from Aurland (${\psi }^{FI}$= 0.039 ± 0.111), with no seasonal variation in ${\psi }^{FI}$ estimated for smolts. The data did not support a population effect in estimates for transition between fjord zones (i.e., *I*, *M*, *O*), in either model.

### Spatial- temporal simulations of migration distance, habitat zone use and survival

**Fig. 8:**
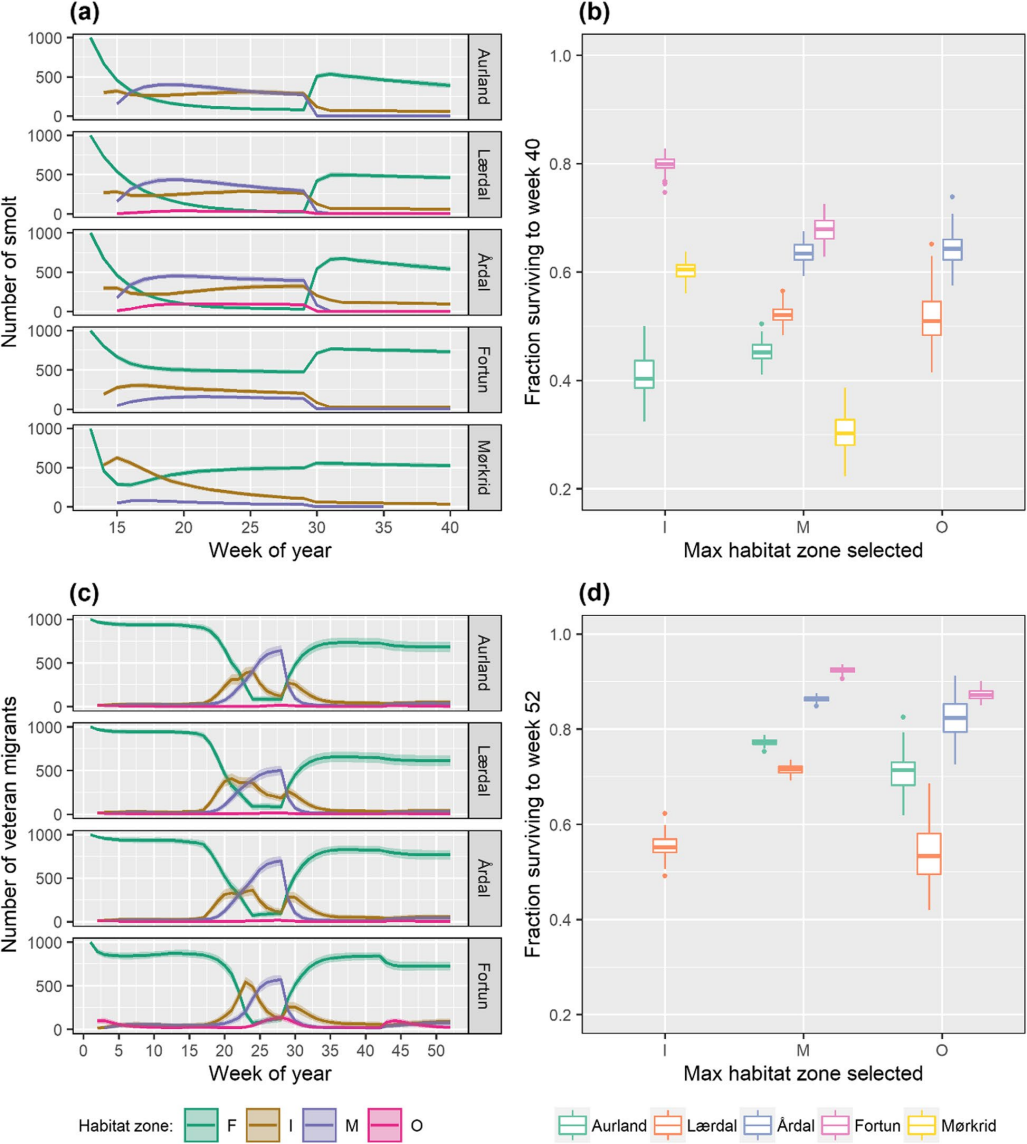
Simulated trajectories of habitat zone use (depicted by colour) for 1000 individual brown trout smolts (**a**) and veteran migrants (**c**) from each study river. Estimates of the maximum zone specific survival rate (${S}_{maxZ}$) of smolts (**b**) and veteran migrants (**d**) presented as the fraction surviving (${S}_{maxZ}= \frac{{N}_{Surv}}{{N}_{Start}}$), dependent upon selection of an individual’s maximum seaward migration distance (fjord habitat zone: inner-, mid- or outer-fjord) and their river of origin. Trajectories represent a six-month period for smolts (WoY 13 – 40), and an annual period for veteran migrants (WoY 1 – 52), with separate simulations run for 2013 – 2015 conditions where panels (**c**) & (**d**) present mean values across years (see Figure S8 for plots separated by year). Shaded regions on panels (**a**) & (**c**) represent 95% CI, resulting from 100 iterations. Boxplots (panels (**b**) & (**d**)) present simulated mean values and 95% CI resulting from 100 iterations. Table 4a and b state mean values of ${S}_{maxZ}$ for each population and life-stage of simulated brown trout

Spatial- temporal simulations of habitat zone use of 1000 individuals per river revealed a seasonal spring exodus of fish from freshwater and into the fjord (Fig. 8a, c). Trajectories show that smolts entered the fjord earlier than veteran migrants, with maximum migration onset occurring in WoY 14 for all smolt populations. During 2013 greatest migration onset occurred during WoY 23 for all populations of veteran migrants. During 2014 and 2015 fish from Årdal and Lærdal were first to migrate (2014: WoY 20, 2015: WoY 19), followed by brown trout from Fortun (2014: WoY 23, 2015: WoY 22) and Aurland (2014: WoY 24, 2015: WoY 21) (Figure S7). The simulations predicted that highest levels of smolt mortality occurred shortly after migration onset, with the greatest losses occurring in WoY 14 for smolts from Aurland (*N* = 35) and Årdal (*N* = 32), and during WoY 16 (*N* = 44), 19 (*N* = 14) and 21 (*N* = 31) for Mørkrid, Fortun and Lærdal smolts, respectively. Mortality of veteran migrants also corresponded with spring out-migration, albeit to a lesser degree, with greatest mortality occurring in WoY 25 (*N* = 19), 22 (*N* = 26), 23 (*N* = 5) and 24 (*N* = 4) for Aurland, Lærdal, Årdal and Fortun fish respectively (mean values across all years). At the end of each simulation, estimated total survival ($\Sigma {N}_{Surv}$) of veteran migrants was 22% greater (mean across populations ± SE: 80.5 ± 0.29%) than that of smolts (55.5 ± 0.23%), despite the different time frames of the simulations (veterans = annual survival, smolts = six months). Variation in survival estimates were also observed among rivers, with a 30% difference in simulated total survival ($\Sigma {N}_{Surv}$) among smolt populations, and a 23% difference among veteran migrant populations (Table 4).

**Table 4:** (a) and (b) Simulated estimates of mean survival and realised fecundity of 1000 individuals, dependent upon population and maximum seaward migration distance (fjord zone; inner-, mid- and outer-fjord) for smolt (a) and veteran migrant (b) brown trout of Sognefjorden

**(a) *Salmo trutta* smolts**							
**Population** (Pop. survival rate: $\Sigma {N}_{Surv}/N$)	**Max migration extent** (Fjord zone)		**N smolts** (${N}_{Start}$)		**N survive** (${N}_{Surv}$)		**Max zone specific survival rate** (${S}_{maxZ}= \frac{{N}_{Surv}}{{N}_{Start}}$)
**Aurland** (0.447)	I		151		62		0.412
	M		849		385		0.453
	O		0				
**Lærdal** (0.521)	I		0				
	M		894		466		0.522
	O		106		55		0.514
**Årdal** (0.638)	I		0				
	M		740		471		0.637
	O		260		167		0.641
**Fortun** (0.754)	I		625		500		0.799
	M		375		254		0.678
	O		0				
**Mørkrid** (0.560)	I		855		516		0.603
	M		145		44		0.303
	O		0				
**(b) Veteran migrant *Salmo trutta* (mean values across years)**							
**Population** (Pop. survival rate: $\Sigma {N}_{Surv}/N$ ± SD)	**Max mig. extent** (Fjord zone)	**N veteran migrants** (${N}_{Start}$)	**N survive** (${N}_{Surv}$)	**Est. fecundity ± SD** ($\overline{{Fec }_{TL}}$)	**Max zone specific survival** (${S}_{maxZ}= \frac{{N}_{Surv}}{{N}_{Start}}$)	**Realised individual fecundity** $(\overline{{Fec }_{TL}}$* ${S}_{maxZ}$)	**Realised population fecundity (%)** $(\Sigma \overline{{Fec }_{TL}}$ * ${N}_{Surv}$)
**Aurland** (0.770 ± 0.001)	I	0					
	M	958	740	2,785 ± 18	0.772	2,151	2.1*e*^6^ (86.0)
	O	42	30	11,298 ± 18	0.709	8,009	3.4*e*^5^ (14.0)
**Lærdal** (0.680 ± 0.004)	I	181	100	843 ± 6	0.554	467	8.5*e*^4^ (4.2)
	M	784	561	3,086 ± 22	0.716	2,208	1.7*e*^6^ (85.4)
	O	35	19	11,208 ± 52	0.537	6,019	2.1*e*^5^ (10.4)
**Årdal** (0.862 ± 0.001)	I	0					
	M	966	834	2,676 ± 13	0.863	2,311	2.2*e*^6^ (87.9)
	O	34	28	11,009 ± 34	0.821	9,039	3.1*e*^5^ (12.1)
**Fortun** (0.909 ± 0.002)	I	0					
	M	714	660	1,738 ± 4	0.924	1,606	1.1*e*^6^ (45.5)
	O	286	250	5,509 ± 30	0.873	4,808	1.4*e*^6^ (54.5)

**Fig. 9:**
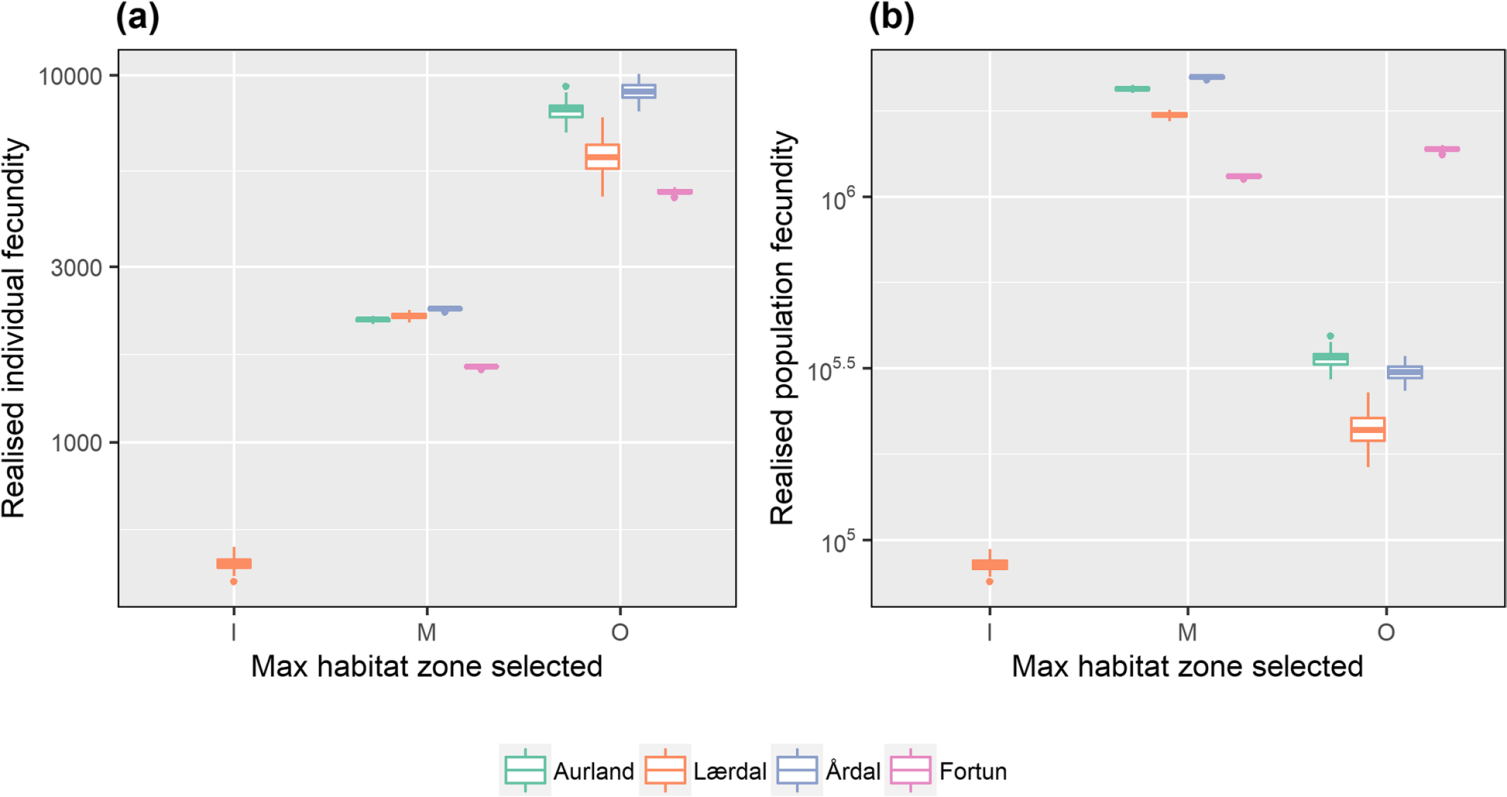
Simulated mean estimates of (**a**) individual and (**b**) population fecundity (N of eggs) of Sognefjord veteran migrant brown trout, dependent upon selection of an individual’s maximum seaward migration distance (fjord habitat zone: inner-, mid- or outer-fjord) and their river of origin. Realised individual fecundity is estimated from the product of average expected size-specific fecundity $(\overline{{Fec }_{TL}}$) and the maximum zone-specific survival rate ${S}_{maxZ}$ (${S}_{maxZ}= \frac{{N}_{Surv}}{{N}_{Start}}$). Realised population fecundity is estimated from the product of total ($\overline{{Fec }_{TL}}$) and mean survival (${N}_{Surv}$). Only individuals predicted to contribute to the spawning population are included in the realised estimates of egg numbers (from the initial population of 1000). Note: Boxplots present simulated mean values and 95% CI resulting from 100 iterations. Simulations were run on an annual basis for 2013 – 2015 conditions, and mean values across all years are presented (see Figure S8 for plots separated by year). Dependant on max zone reached, TL at end of growth season was estimated as${TL}_{rep}={TLe}^{{g}_{SW2(maxZ)}* 0.5}$, where sea growth was estimated from 2^nd^ sea age specific growth (${g}_{SW2}$). Size-specific estimates of fecundity ($\overline{{Fec }_{TL}}$) were generated according to${Fec}_{TL}={e}^{-4.03+2.74* {TL}_{Rep}}$. Only individuals contributing to the spawning population (${TL}_{Rep}$> 35 cm and retuned to freshwater during the period WoY 37 – 52), were included in the estimates of realised fecundity. Table 4b states mean values of realised individual and population fecundity for each study population

Estimates are derived from the simulated trajectories of migrant brown trout habitat use for 1000 individuals from each study population (Fig. 8a, c), and for veteran migrants during 2013 – 2015 conditions (Figure S7). Values presented are the means and standard deviations (SD) derived from 100 iterations of each simulation (see Figs. 8 and 9 for confidence intervals). Dependant on max zone reached, TL at end of growth season was estimated as ${TL}_{rep}={TLe}^{{g}_{SW2(maxZ)}* 0.5}$, where sea growth was estimated from 2^nd^ sea age specific growth (${g}_{SW2}$). Size-specific estimates of fecundity ($\overline{{Fec }_{TL}}$) were generated according to ${Fec}_{TL}={e}^{-4.03+2.74* {TL}_{Rep}}$. Only individuals contributing to the spawning population (${TL}_{Rep}$> 35 cm and retuned to freshwater during the period WoY 37 – 52), were included in the estimates of realised fecundity

The trajectories of anadromy predicted that only smolts originating from Lærdal (10.6%) and Årdal (26.0%) reached the outer-fjord zone, with the mid-fjord simulated as the maximum seaward migration distance for the remaining three populations (${N}_{Start}$). Veteran migrants from all populations reached the outer-fjord zone, with the mid-fjord zone simulated as the maximum migration distance for residual migrants, except for 18.1% of fish originating from Lærdal, which ceased their migration in the inner-fjord zone. For both smolts and veteran migrants estimated survival rate (${N}_{Surv}$) was lowest in the outer-fjord (mean ± SE: 0.11 ± 0.079, 0.08 ± 0.101, smolts and veteran migrants respectively), and for smolts highest in the inner-fjord zone (0.36 ± 0.257), with survival of smolts in the mid-fjord estimated as 32.4% (± 17.9%). Whereas estimated values of ${N}_{Surv}$ were highest in the mid-fjord zone for veteran migrants (0.70 ± 0.105), with mean survival of migrants to the inner-fjord zone estimated as just 10.0% (± 0.26%) (Table 4).

The trajectories revealed that return migration into freshwater was initiated in the autumn, with smolts returning earlier than veteran migrants. Of the initial population of 1000 individuals, a total of 53.6, 49.6, 67.5, 76.6 and 55.8% of smolts returned to freshwater from Aurland, Lærdal, Årdal, Fortun and Mørkrid populations, respectively, with maximum return of smolts occurring between WoY 30 – 32. A total of 76.8, 67.9, 86.1 and 90.4% of the initial population of veteran migrant brown trout returned to the rivers Aurland, Lærdal, Årdal and Fortun respectively (mean values across all years), with these maximum levels of returning migrants transpiring between WoY 36 (Lærdal) and 42 (Fortun). Almost all (99.7%, mean value across rivers and years) veteran migrants surviving the duration of the simulation returned to freshwater, where the majority (65.8%, mean across rivers and years) resided for the remainder of the simulated period. The proportion of autumn migrants returning to the fjord to overwinter (between WoY 37 – 52) was simulated as 0.30, 0.28, 0.31 and 0.47 for Aurland, Lærdal, Årdal and Fortun populations, respectively. Of the 0.3% of veteran migrant brown trout that survive the duration of the simulation but fail to return to freshwater 89. 5% of these fish were larger than 35 cm. These ‘skipped spawners’ accounted for 0.1% of the total surviving population of veteran migrants in all rivers, except for Fortun fish, where 0.6% of the surviving population failed to return to freshwater (Fig. 8c).

### Simulated expression of anadromy, survival and relative contribution to fecundity

Within populations, variation in the maximum zone-specific survival rates (${S}_{maxZ}$) of smolts was simulated. For smolts from Fortun and Mørkrid estimates of ${S}_{maxZ}$ were 12 and 30% higher for those smolts selecting the inner-fjord zone as their maximum extent of anadromy (Table 4a). This observation was reversed for Aurland smolts, with a greater proportion surviving when selecting the mid-fjord as their maximum habitat zone (4.1% difference). A limited difference in ${S}_{maxZ}$ between migrants to the mid- and outer-fjord zones for Lærdal and Årdal smolts was estimated (Fig. 8b). Disparity in ${S}_{maxZ}$ within populations of veteran migrants was also simulated, albeit to a lesser degree than for smolts. Greatest variation in ${S}_{maxZ}$ (25%) was estimated for Lærdal fish, the only population in which individuals (18.1% of the initial population) were simulated as ceasing their migration in the inner-fjord zone (Table 4b). In all populations ${S}_{maxZ}$ of veteran migrants was higher for mid-fjord than for outer-fjord zone migrants, with a difference of 6.3, 17.8, 4.2 and 5.1% for Aurland, Lærdal, Årdal and Fortun fish, respectively (mean values for all years), with ${S}_{maxZ}$ (± SD) estimated as 55.4% (± 4.1%), 81.9% (± 8.2%) and 73.5% (± 14.3%) for fish reaching the inner-, mid- and outer-fjord zones respectively (mean values for all populations and years) (Fig. 8d). Despite higher simulated rates of ${S}_{maxZ}$ for veteran migrants reaching the mid-fjord zone, estimated values of realised individual fecundity were 70% (mean across years and populations) greater for outer-fjord migrants from all populations, resulting from the higher fecundity (${Fec}_{TL}$) of the larger individuals which reach this fjord zone (Table 4b, Fig. 9a). Within populations, the greatest difference in realised individual fecundity was estimated for Lærdal brown trout (a difference of 5,552 eggs (92%) between inner- and outer-fjord migrants), reflecting the reduced fecundity of the smaller migrants which migrated no further than the inner-fjord (Table 4b). Conversely, migrants to the mid-fjord zone were the main contributors to realised population fecundity, with simulated mean values 83.7, 87.8 and 86.2% greater than those of outer-fjord migrants for Aurland, Lærdal and Årdal brown trout, respectively (Fig. 9b). Only in the river Fortun, was the greatest contribution to realised population fecundity estimates produced by outer-fjord zone migrants, reflecting the high number of surviving fish (*N* = 250) within this zone, when compared to the other study rivers (mean *N* = 26). Limited difference (0.10%) in estimated values of realised population fecundity was observed among years, with a difference of 9,242 eggs between the most successful (2015) and least successful (2014) years (Figure S8b). However, a difference among rivers was revealed with fish from Lærdal estimated to contribute the least (21.4%) and Årdal most (26.8%) to the total brown trout egg production of Sognefjorden (Table 4b).

## Discussion

Our findings reveal that individual traits of anadromous behaviour influence potential exposure to risk within the fjord, but that potential mortality costs are balanced against the gains of increased growth and in turn reproductive success. This compromise between survival and reproduction is considered paramount in life-history theory [73, 74], requiring the balance tipped in favour of survival and growth for the preservation of anadromy [1]. Our findings suggest that this trade-off likely underlies the migratory behaviour observed in Sognefjorden brown trout, with those individuals that migrate greater distances whilst foraging at sea being larger with greater fecundity than their smaller, less risk-taking counterparts.

### Modelling of brown trout habitat use and migratory behaviour

By selecting five different natal populations that drain into a single fjord, we were able to, as much as possible under natural conditions, fix the growth potential in the marine environment for brown trout individuals from all populations. Thereby allowing for assessment of population-level effects and the significance of the freshwater phase in the stimulation and nature of anadromous strategy. We postulated that the extent and duration of anadromy would vary among populations. We observed that both fish length and natal population were key in describing the maximum seaward extent of anadromy for both smolts and veteran migrants (Fig. 4), but that these factors contributed little to explaining the duration of fjord use. Instead, spatial and temporal factors influenced the duration of sea-sojourn, with long-distance migrants remaining in the fjord for longer than short-distance migrants. For veteran migrants, for which annual data was collected, duration of fjord-zone use was also influenced by season (Fig. 5b).

Few smolts underwent long-distance migrations, with the majority of first-time migrants not journeying further than the inner/mid fjord regions. Only Mørkrid fish were predicted to migrate to a distance greater than 100 km, with the expected maximum distance of the remaining populations extending to no more than a third of the total length of Sognefjorden. Similar behaviour was also observed in the veteran migrant brown trout. Although some individuals from all populations reached the outer-fjord zone, only fish from Aurland and Fortun were predicted to migrate to a distance greater than 100 km, limited to just half of the total length of Sognefjorden. Contemporary studies of anadromous brown trout often observe that studied fish remain in estuaries, as opposed to migrating to adjacent marine habitats, particularly within Norwegian fjord systems [24, 25, 35]. Presumably, this reflects the suitability of the near-adjacent habitats [75] and the resulting expenditure required to reach the open sea in these long, semi-enclosed fjord systems, with marine migration distance in Norwegian brown trout thought to reflect the size of the fjord they belong to [17]. Whereas anadromous brown trout from coastal Danish systems, have been shown to migrate greater distances, up to 580 km from their natal river, likely because they encounter a less suitable and more precarious environment once they leave freshwater, therefore inducing longer-distance migrations [26, 76]. Body size has been shown to play a central role in the migration performance of aquatic animals, as swimming ability and migration speed potential increases with body mass [77]. In anadromous brown trout, body length has previously been shown to have a positive effect, with larger fish migrating further [35, 38], or no effect on migration distance [22, 33, 36]. Body size has also been linked to the duration of fjord-use, with larger brown trout reported to remain within the fjord for longer than smaller individuals, in both smolt and veteran migrant brown trout [36]. Eldøy et al. [38], show that poor body condition has also been linked to a greater probability of individuals remaining at sea longer. However, in contrast to our findings, the authors find no correlation between migration distance and the duration of marine residence time. Instead, we demonstrate that migration distance is linked to duration of fjord-use, with those individuals undertaking longer migrations remaining in the fjord for greater periods, possibly a result of the geography of the Sognefjord system, typified by its extensive length and semi-enclosed nature. For veteran migrants, for which annual data was collected, residence duration in each of the spatial zones was also shaped by season, most visibly in the use of freshwater. Unsurprisingly, veteran brown trout utilised freshwater most in the autumn and winter, and least in the spring, where they predominantly resided within the fjord. However, no population-level effect was revealed in the model, with greater variation in behaviour observed among individuals than among populations (Fig. 5b and Figure S5). Our model approach describing the duration of fjord-use for smolts was poor, as reported by previous telemetry studies [33, 35]. We suggest that this may in part be due to the limited timeframe of the data, camouflaging any seasonal effect of zone use, with the batteries of most smolt tags expiring after six months and prior to the freshwater return of individuals with long fjord-use durations. Anadromous brown trout are considered opportunistic in their feeding behaviour, with seasonal variation in diet and habitat use observed whilst at sea [78, 79]. Thus, habitat use of brown trout in the fjord likely follows preferential prey over temporal and spatial scales, and likely accounts for much of the individual variation observed in duration of fjord use and fish depth (Figure S6). There is clearly a need to further investigate the origins of this individual variability in migratory behaviour, as there is much to be gained in our understanding of the spatial ecology of brown trout.

We also hypothesised that environmental conditions in each river will affect the propensity and timing of migration. Indeed, river of origin was key in explaining much of the variation in migration onset observed, with environmental drivers influencing the timing of migration onset in veteran migrants (Fig. 5a), but with limited effect for first-time migrants. The importance of environmental cues in the timing of migration onset of salmonids has been observed many times elsewhere, with downstream migration triggers commonly cited as water level, flow and temperature [9, 80–82]. For Sognefjorden veteran migrants, combined effects of water discharge and daily change in discharge were shown to be important in inducing migration onset, but to a varying degree among the four populations, potentially reflecting population level adaptations to ensure optimal timing and conditions for migrants at sea entry [3, 31, 83]. The additional effect of fish length within the migration onset model, suggests that optimal timing of migration also varies within populations. Therefore, despite conditions at fjord entry presumably equal, or close to, for all four populations, optimal timing of migration appears to result from a complex interaction of individual body length and environmental cues for each population. We found no relationship between water level or discharge and the timing of migration onset for smolts, with limited support for the models fitted to the smolt data observed. Instead, we observed large variation in fjord-entry dates both among and within populations, with the period of migration onset spanning several months. In Sognefjorden, migrant smolts were larger and had a lower condition factor than fish remaining in freshwater (Figure S4), but we were unable to link these factors to the timing of migration onset. However, the mark-recapture model revealed that the probability of transitioning into the marine environment was influenced by natal river (Fig. 6). Clear differences were observed among rivers, with smolts from Lærdal (*ψ*^*FtoI*^ = 0.382) almost ten times more likely to enter the fjord during a two-week period in spring than smolts from Aurland (*ψ*^*FtoI*^ = 0.039), those least likely to migrate. As our sampling was directed at migrant brown trout, we were unable to reliably determine the proportion of sympatric resident and migrant individuals within each natal population. Instead, we were able to infer the influence of growth potential among natal rivers and the consequences for migratory behaviour, with natal river key to explaining much of the variation observed. We acknowledge that ideally resident and anadromous brown trout life-histories should be considered simultaneously, and in this study, where we focused on sea migrating individuals, we could not compare the risks and rewards of the migration strategies observed with those of freshwater resident individuals. However, our findings suggest that fjord use reflects the behavioural plasticity/flexibility of brown trout, where for instance day-to-day variation in environmental conditions, threats and opportunities (e.g., water currents, predator presence, food availability etc.) can impact daily choice of migration path, creating numerous migratory pathways among individuals, even among those that have the same start and end points of migration.

### Spatial- temporal simulations of survival

Despite an influx of recent research, many questions remain regarding the survival and habitat use of brown trout at sea. The application of mark-recapture modelling allowed us to evaluate seasonal and spatial variation in survival for each population and brown trout life-stage. This is considerably more informative than estimating survival on the proportion of individuals that return to freshwater, which is largely common practice in salmonid telemetry studies (but see e.g., [30, 55, 83]). We hypothesised that mortality risk would vary spatially and temporally within the fjord. However, contrary to expectations, the smolt mark-recapture model estimated survival probabilities that were constant throughout the different fjord zones, and instead discrete population-level survival probabilities were realised (Table 3a, Fig. 6). Thus, river specific conditions such as temperature, prey availability, predation risk and water discharge levels experienced prior to smolt migration, likely contribute to differential survival among populations within the fjord. We do however acknowledge that limited numbers of smolts were detected in the outer-fjord, potentially limiting the estimates of the mark-recapture model for this zone. The resulting simulations which combined discrete population-level survival probabilities and river-specific estimates of migration propensity, distance and fjord duration, yielded an unequal cost of anadromy among the five smolt populations. This was ultimately estimated as a 30-percentage points difference in simulated total survival (${\Sigma N}_{Surv}$) between the populations with lowest and highest survival (range 45 – 75%) (Table 4a). These estimates are generally higher than prior reported return rates, although these are also extremely variable among study systems. For example, mean survival of smolts from the River Imsa (Norway) between the years 1976 to 2005 was estimated as just 15% [84], whereas a return rate of 65% into the River Søa (Norway) was considered an underestimated value in a telemetry study [35], with the latter value more comparable to the simulated survival estimates of smolts in Sognefjorden. Reported marine survival rates for veteran migrant brown trout are higher, but also variable. For example, sea survival of kelts was reported as between 30 – 60% in Storelva, southern Norway [30], while Bordeleau et al. [41] reported an 86% return to freshwater of tagged veteran migrant brown trout from Åbjøra and Urvold, mid Norway. Considerable inter-population variation in the simulated estimates of $\Sigma {N}_{Surv}$ of veteran migrants was also produced within Sognefjorden, albeit to a lesser degree than for smolts (range: 68 – 91%) (Table 4b), with the highest total survival estimates greater than previously reported values. Contrary to the smolt model, the veteran migrant mark-recapture model produced estimates of survival that varied both spatially and seasonally within the fjord, although a population effect also contributed (Table 3b, Fig. 7). We emphasise however, that the limited timeframe of the smolt mark-recapture model, due to the restricted battery capacity of the smaller fish tags, may have masked seasonal or temporal effects on smolt survival estimates. Nonetheless, the simulations did reveal that greatest mortality loss occurred shortly after migration onset in the spring and was particularly evident in smolts, with a weekly loss of up to 4.4% (Lærdal smolts) simulated. The downstream migration is regarded as a critical transition phase between freshwater and the marine environment in salmonids [22, 31, 57], with mortality expected to be highest during the first 14 days after sea entrance [85]. This was reflected in the mark-recapture models, with survival estimates generally higher in freshwater than within the fjord (Table 3). We also anticipated that the risk of anadromy would be greater for smolt than for veteran migrant brown trout. Indeed, total simulated summer survival of smolts was estimated as 56% and total annual survival of veteran migrants estimated as 81% (mean values across populations). Sea survival of first-time migrants is considered a bottleneck in the anadromous salmonid lifecycle [31, 84], as mortality is often acknowledged to be size dependent. Unfortunately, we were unable to evaluate the effect of body length or condition on survival, due to limitations in our data, though we emphasise future studies should aim to do so given the importance of fish metabolism and physiology in the processes determining the propensity, duration and extent of anadromy in brown trout [86, 87].

The simulations also revealed that values of ${N}_{Surv}$ differed according to the maximum migration distance of Sognefjorden brown trout. Maximum ${N}_{Surv}$ was simulated for veteran migrants reaching the mid-fjord zone (70%), with this reduced to just 8% for fish in the outer-fjord zone (Table 4b). Although constant survival probabilities were estimated across fjord zones in the smolt mark-recapture model, when these estimates were weighted as an exponent of observed residence duration within each of the habitat zones, this also resulted in discrete values of ${N}_{Surv}$ being simulated, conditional on migration distance (Fig. 6). For smolts, minimum ${N}_{Surv}$ was also simulated for outer-fjord migrants (11%), but maximum ${N}_{Surv}$ was instead simulated for smolts remaining in the inner-fjord (36%) (Table 4a). Consequently, these simulations indicate that a distinct mortality risk occurs among the different spatial zones of Sognefjorden. The inner-fjord region is less risky, especially for smolts, whereas the culmination of factors encountered along the extensive migration route, resulted in a substantially increased risk of mortality in the outer-fjord region. The combination of increased energetic expenditure, fishing pressure, predation and salmon lice exposure likely contributes to the high mortality rates for migrants to this zone. The application of simulated data in this context enables us to draw conclusions that explicitly link survival with migration duration and distance. This is difficult to achieve whilst relying solely on telemetry data, as behavioural observations are inherently derived from the returning/surviving individuals, thus the full duration and extent of marine use for lost individuals remains unknown. We therefore reason that, providing sufficient empirical data allows robust models to be built, the integration of telemetry data and mark-recapture modelling provides a more comprehensive approach to the understanding of anadromy. By deriving simulated trajectories from these models, a more complete picture is generated, which includes not only those individuals that return to freshwater (or remain in the fjord) but also those that die whilst at sea. To our knowledge this data is unique and provides a relevant and significant insight into the survival and behaviour of anadromous brown trout.

In addition to spatial estimates of mortality risk, simulated migration trajectories allowed us to evaluate survival on a temporal scale, and for the veteran migrant brown trout simulations were generated for 2013 – 2015 conditions. However, due to limited annual data being included as model predictors, little variation among years was observed (Figure S7). We surmise that the inclusion of more environmental data, particularly within the fjord (e.g., water temperature, salinity, currents), would generate greater temporal variation in habitat use and consequently survival among years, with inter-annual variation in brown trout sea survival observed in previous telemetry studies [80, 84, 88].

### Simulated expression of anadromy and relative contribution to fecundity

The expression of anadromy is believed to be driven by concession between mortality risk and growth potential in different habitats, with the most beneficial strategy differing among individuals and populations [21, 22]. We therefore anticipated that alternate strategies in the maximum seaward migration distance would be observed in individual brown trout, and that the cost of individual selection in the extent of anadromy would vary both among and within populations. Indeed, simulated trajectories revealed that the proportion of migrants reaching the inner-, mid- and outer-fjord regions varied substantially among and within populations as well as life-stage, with intra-population variation in expression of anadromy greatest for smolts (Table 4, Fig. 8). In smolts, modelled transition estimates between fjord zones revealed that the probability of migrating further than the inner-fjord was low (19%), but that if the mid-fjord was reached, the probability of migrating further and reaching the outer-fjord was almost three-fold (52%). Similar behaviour has been described previously in brown trout smolts, where within the continuum of migration a decision point occurs, at which brown trout will assess the capacity of an extended migration versus an alternative of remining in the near-fjord [22, 80]. Whereas transition estimates for veteran migrants showed that these fish are more mobile between fjord zones, presumably due to their larger body size, thus benefiting from improved swimming ability and limited predation [89]. We defined the cost of selection for a given migration strategy by calculating the maximum zone-specific survival rate (${S}_{maxZ}$), the fraction surviving dependent upon expression of simulated migration distance. Within populations, variation in ${S}_{maxZ}$ of smolts was simulated, albeit with contrasting results (Table 4a, Fig. 8b). For the populations located inner-most within Sognefjorden (Fortun and Mørkrid) estimates of ${S}_{maxZ}$ were considerably higher for smolts remaining in the inner-most fjord arm. Whereas Aurland mid-fjord migrant smolts benefited from slightly higher rates of ${S}_{maxZ}$ than individuals remaining in the inner fjord. Whilst limited differences were estimated for Lærdal and Årdal smolts, the two populations that had individuals reaching the outer fjord. This therefore implies that the cost of anadromy reflects an inequality in the energy expenditure required to reach the different fjord zones, dependent upon the location of natal river within Sognefjorden. The combination of reduced swimming performance due to the smaller body length of these fish and the extensive distances between fjord zones, likely exaggerates this observation for smolts in the Sognefjord system. Discrepancy in ${S}_{maxZ}$ within populations of veteran migrants also resulted from the simulations, albeit to a lesser degree than for smolts. Considerable variation in ${S}_{maxZ}$ was estimated for Lærdal fish, the only population for which some individuals were simulated as ceasing their migration in the inner-fjord zone. Notably, ${S}_{maxZ}$ estimates were higher for mid-fjord than for outer-fjord zone migrants for all populations of veteran brown trout. These findings reiterate that migration to the outer-fjord region of Sognefjorden is associated with an increased mortality risk, irrespective of river of origin.

The ability to survive, reproduce and contribute genetically to the next generation determines individual fitness [90]. We therefore anticipated that an anadromy-associated increase in mortality risk would be balanced against fitness gains, in the form of improved growth and fecundity, at both the individual and population level [4]. We observed a trend for superior individual growth rate in brown trout reaching the mid- and outer fjord regions, which in turn boosted fecundity estimates in these fish. In salmonids, growth is an important contributor to reproductive success, particularly in females, with the number of eggs increasing with body size [60]. In an iteroparous species like the brown trout, repeated sea sojourn events (e.g., [91]) will likely provide additional fitness gains beyond just increased numbers of offspring, via maternal effects related to higher quality of offspring and the provisioning of better spawning habitats for their offspring [18, 20, 92]. Simulated values of realised individual fecundity were on average 70% greater for outer-fjord migrants, a pattern observed in all populations. However, due to a poor survival rate in this zone, in combination with a depleted number of individuals reaching the outer-fjord, migrants to the mid-fjord zone overwhelmingly constituted the main contributors to realised population fecundity, with total egg contribution 86% higher for mid-fjord migrants than those of outer-fjord migrants (Table 4b and Fig. 9). However, this pattern was reversed for fish from Fortun, albeit with limited difference in egg numbers between the two groups. The inner-most location of this river within Sognefjorden means that the distances journeyed by these fish to reach the mid- and outer-fjord regions are the most extensive of all populations studied. Interestingly, the simulated trajectories also revealed that the proportion of autumn migrants, i.e., return to the fjord to overwinter post-spawning, was highest for Fortun fish (47%), as was the proportion of skipped spawners. Both these strategies are suggestive of a form of energy conservation [15, 30], a strategy that may have been selectively favoured for these individuals that would face the highest migration costs if adopting a long-distance migration strategy. It may be that Fortun brown trout have developed this seemingly successful strategy of migratory behaviour in more recent times, potentially resulting from anthropogenic-induced environmental alterations within the fjord. Or, that this has always been present as an energy-saving strategy to overcome the physiological costs of undertaking the extensive journey required for this population to reach the open coast and improved feeding opportunities in this fjord system.

Anadromy in brown trout has been described as a continuum from freshwater residency to complete anadromy [15] and del Villar-Guerra et al. [22], suggest that within this continuum a decision point exists, where individuals will assess the cost of continuing towards open sea versus the benefit of remaining in near shore and estuarine waters. It has also been suggested that brown trout are able to adapt their migratory behaviour in response to increased mortality risk. e.g. high salmon lice concentrations, but with a cost of reduced growth opportunity [46]. Thus, expression of anadromy in brown trout is clearly adaptable, dependent upon perceived stressors and risks. This is particularly relevant given increased anthropogenic induced stressors in coastal regions, which may alter or be in the process of altering the interaction of factors driving facultative migrations. In Sognefjorden brown trout anadromy is flexible, with the cost of extended migration balanced against the reward of improved growth and fecundity. However, this balance appears to be precarious, with the overwhelming contribution to population fecundity being derived from middle-distance migrants, and not from larger, long-distant migrants. Although speculative, this may result in negative demographic consequences for the total population, given that offspring growth and viability is improved with larger and older parents [19, 20].

### Historical versus contemporary seaward extent of anadromy

The ultimate aim of this study was to assess if the degree of anadromy had diminished when compared to 60-year-old mark recapture data of anadromous brown trout from the region. Historical recapture locations of Lærdal brown trout were located at a greater distance (33%) from the river mouth (87.7 ± 70.3 km), when compared to the maximum migration distances of their present-day counterparts (58.6 ± 54.9 km) (Fig. 2). However, no difference in the estimates of sea specific growth were observed between past and present Lærdal scale samples, indicating that the growth potential within the fjord has not altered significantly within this timeframe. The historical recapture data demonstrates that prior to the development of hydropower and salmon aquaculture, Lærdal brown trout journeyed the full extent of Sognefjorden and beyond, along the open coastline (Fig. 2b). We emphasize that these distances are not directly comparable to the values of maximum marine migration distance generated by acoustic telemetry, instead these capture locations represent fish presence, with the maximum seaward extent of migration unknown for these individuals (i.e., individuals caught in inner and mid-fjord locations may have been on their way to outer-fjord areas). Unfortunately, this allows for limited scope of quantitative assessment, with methodological bias in recapture data problematic, fish will only be recaptured where fishing is taking place [27]. However, we also recognize that the data is unique in the evidence it provides, with comparatively few of the anadromous brown trout tagged during this study reaching the outer-most receivers at their maximum seaward extent. Anadromous brown trout are clearly capable of migrating the entire length of Sognefjorden, during both contemporary and historical times, but unfortunately evidence points to a diminished extent of migration in this unique system. It has been suggested that the processes of natural selection may be lagging with respect to the rapid alterations occurring in the relatively recent Anthropocene. Thus brown trout remain anadromous despite evidence of greater costs whilst undertaking feeding migrations at sea [6]. We reason that the expression of contemporary anadromy within Sognefjorden lends support to this notion, and that reduced marine growth and increased mortality will ultimately reduce the benefit of extended marine migrations, with long distance sea-sojourn vulnerable to being potentially lost or partly lost from the brown trout populations of Sognefjorden.

## Conclusions

Sognefjorden, the longest fjord system that supports populations of anadromous brown trout in the world, provides a unique opportunity in which to study the expression of anadromy, where the energetic expenditure required to migrate the 200 km to reach the open coast is presumably considerable. In modern times, anthropogenic alterations in the river-to-sea environment create additional stressors during this extensive sea-sojourn which may ultimately tip the cost–benefit balance of anadromy in Sognefjorden toward alternate life-history strategies. We conclude that present-day anadromy in Sognefjorden is precarious, but potential risk varies considerably between life-stages and populations, even within a single fjord system. Within populations, long-distance migrants are rewarded with greater fecundity, however the main contribution to population fecundity is derived from middle-distance migrants, due to higher rates of survival and limited numbers of long-distant migrants. Our findings suggest that selection for extended feeding migrations is potentially under pressure, with the degree of contemporary anadromy seemingly diminished when compared to data collected prior to aquaculture and hydropower development, which are now ubiquitous in the region. We therefore stress the importance of monitoring and decisive management actions to secure genetic variation pertinent to preserve the fitness gains of anadromy.

### Supplementary Information

**Additional file 1: Table S1. **Overview and technical specifications of the acoustic tags deployed in smolt (a) and veteran migrant (b) brown trout from Sognefjorden. **Figure S1.** Detection data of tagged Sognefjord *Salmo trutta* smolts. **Figure S2.** Detection data of Sognefjord *Salmo trutta* veteran migrants. **Figure S3.** Boxplots showing the back calculated estimates of first- and second- year sea specific growth rate. **Table S2.** AIC scores of the models generated to describe the migration and habitat use of tagged *Salmo trutta* smolts and veteran migrants in Sognefjorden. **Table S3.** Summary statistics from the models used to describe the migration and habitat use of tagged brown trout smolts and veteran migrants in Sognefjorden. **Figure S4.** Box plots showing median *TL* and *K* of migrant and resident immature Sognefjord brown trout. **Table S4.** Summary statistics of veteran migrant brown trout residence duration within each given habitat zone of Sognefjorden. **Figure S5.** Total residence duration of tagged brown trout smolt migrants within each given habitat zone of Sognefjorden.**Figure S6.** Mean annual depth use of individual veteran migrant brown trout. **Table S5.** Summarised seasonal depth use of veteran migrant brown trout within each given habitat zone of Sognefjorden. **Table S6.** AIC scores of candidate conditional Arnason-Schwarz (CAS) mark-recapture models to estimate rates of survival (S), recapture (detection) (p) and transition (migration) (Psi) of tagged Sognefjord brown trout smolts. **Table S7.** Logit parameter estimates for the selected conditional Arnason-Schwarz (CAS) mark-recapture model of tagged Sognefjord brown trout smolts. **Table S8.** AIC scores of candidate conditional Arnason-Schwarz (CAS) mark-recapture models to estimate rates of survival (S), recapture (detection) (p) and transition (migration) (Psi) of tagged Sognefjord veteran migrant brown trout. **Table S9.** Logit parameter estimates for the selected conditional Arnason-Schwarz (CAS) mark-recapture model of tagged Sognefjord veteran migrant brown trout**. Figure S7.** (a) Simulated trajectories of habitat use for 1000 individual veteran migrant brown trout, from each study river and for 2013 – 2015 conditions. (b) Estimates of the maximum zone specific survival rate, for each study river and for 2013 - 2015 conditions.** Figure S8.** Simulated estimates of (a) individual and (b) population fecundity of Sognefjorden veteran migrant brown trout, dependent upon maximum migration extent and river of origin, presented for 2013 – 2015 conditions.

## Data Availability

The data sets supporting the conclusions of this study are deposited in the NMBU Open Research Data database https://dataverse.no/dataset.xhtml?persistentId=doi:10.18710/LIYHRV.
