# Supplementary material for: Balancing risks and rewards of alternate strategies in the seaward extent, duration and timing of fjord use in contemporary anadromy of brown trout (Salmo trutta)
Source: BMC Ecol Evol. 2024 Feb 29;24:27. doi: 10.1186/s12862-023-02179-x (PMC10903050; doi:10.1186/s12862-023-02179-x)
Supplement: Supplementary file 1 — Additional file 1: Table S1. Overview and technical specifications of the acoustic tags deployed in smolt (a) and veteran migrant (b) brown trout from Sognefjorden. Figure S1. Detection data of tagged Sognefjord Salmo trutta smolts. Figure S2. Detection data of Sognefjord Salmo trutta veteran migrants. Figure S3. Boxplots showing the back calculated estimates of first- and second- year sea specific growth rate. Table S2. AIC scores of the models generated to describe the migration and habitat use of tagged Salmo trutta smolts and veteran migrants in Sognefjorden. Table S3. Summary statistics from the models used to describe the migration and habitat use of tagged brown trout smolts and veteran migrants in Sognefjorden. Figure S4. Box plots showing median TL and K of migrant and resident immature Sognefjord brown trout. Table S4. Summary statistics of veteran migrant brown trout residence duration within each given habitat zone of Sognefjorden. Figure S5. Total residence duration of tagged brown trout smolt migrants within each given habitat zone of Sognefjorden.Figure S6. Mean annual depth use of individual veteran migrant brown trout. Table S5. Summarised seasonal depth use of veteran migrant brown trout within each given habitat zone of Sognefjorden. Table S6. AIC scores of candidate conditional Arnason-Schwarz (CAS) mark-recapture models to estimate rates of survival (S), recapture (detection) (p) and transition (migration) (Psi) of tagged Sognefjord brown trout smolts. Table S7. Logit parameter estimates for the selected conditional Arnason-Schwarz (CAS) mark-recapture model of tagged Sognefjord brown trout smolts. Table S8. AIC scores of candidate conditional Arnason-Schwarz (CAS) mark-recapture models to estimate rates of survival (S), recapture (detection) (p) and transition (migration) (Psi) of tagged Sognefjord veteran migrant brown trout. Table S9. Logit parameter estimates for the selected conditional Arnason-Schwarz (CAS) mark-recapture model of tagged S [file 12862_2023_2179_MOESM1_ESM.pdf]

BMC Ecology and Evolution

Appendix S1: Supplementary Tables and Figures

Balancing risks and rewards of alternate strategies in the seaward extent, duration and timing of fjord use in contemporary anadromy of brown trout (*Salmo trutta*)

K. L. Hawley, H. Urke, T. Kristensen and T. O. Haugen

Table S1: Overview and technical specifications of the acoustic tags deployed in smolt **(a)** and veteran migrant **(b)** brown trout from Sognefjorden. All tags were produced by Thelma Biotel AS, Trondheim, Norway.

(a) Smolt tags

**Tag spec.**	**LP-7.3**	**LP-7.3**	**LP-7.3**
Tag type	ID	ID	ID
Tag diameter (mm)	7.3	7.3	7.3
Tag length (mm)	17	17	17
Weight in air (g)	1.8	1.8	1.8
Transmit interval (sec)	30/90	60/120	180/240
Power output (dB)	139	139	139
Battery life (months)	6	7	11
Number deployed: 2012	0	0	0
Number deployed: 2013	0	0	39
Number deployed: 2014	20	107	0
Number deployed: 2015	0	53	0
**Total deployed:**	**20**	**160**	**39**

(b)Veteran migrant tags

**Tag spec.**	**LP-7.3**	**LP-7.3**	**LP-7.3**	**LP-9**	**MP-9-SHORT**	**ADT-9-LONG**	**ADT-13-STAT**
Tag type	ID	ID	ID	ID	ID	ID/ Depth/ Temp	ID/ Depth/ Temp
Tag diameter (mm)	7.3	7.3	7.3	9	9	9	12.7
Tag length (mm)	17	17	17	24	24.4	29.4	33.3
Weight in air (g)	1.8	1.8	1.8	4	3.6	5.2	7.1
Transmit interval (sec)	30/90	60/120	180/240	30/90	30/90	90/270	120/360
Power output (dB)	139	139	139	142	146	146	153
Battery life (months)	6	7	11	20	9.5	15	31
N deployed: 2012	0	0	0	0	0	50	30
N deployed: 2013	0	0	5	0	39	61	61
N deployed: 2014	3	3	0	0	0	73	0
N deployed: 2015	0	10	0	20	0	0	0
**Total deployed:**	**3**	**13**	**5**	**20**	**39**	**184**	**91**

Figure S1: Detection data of tagged Sognefjord *Salmo trutta* smolts (N = 175), sorted by day of year (DoY) and habitat zone (F = freshwater, I= inner-fjord, M = mid-fjord, O = outer-fjord). Figures are coloured according to natal river; data is presented for all sampling years (2012 – 2014).

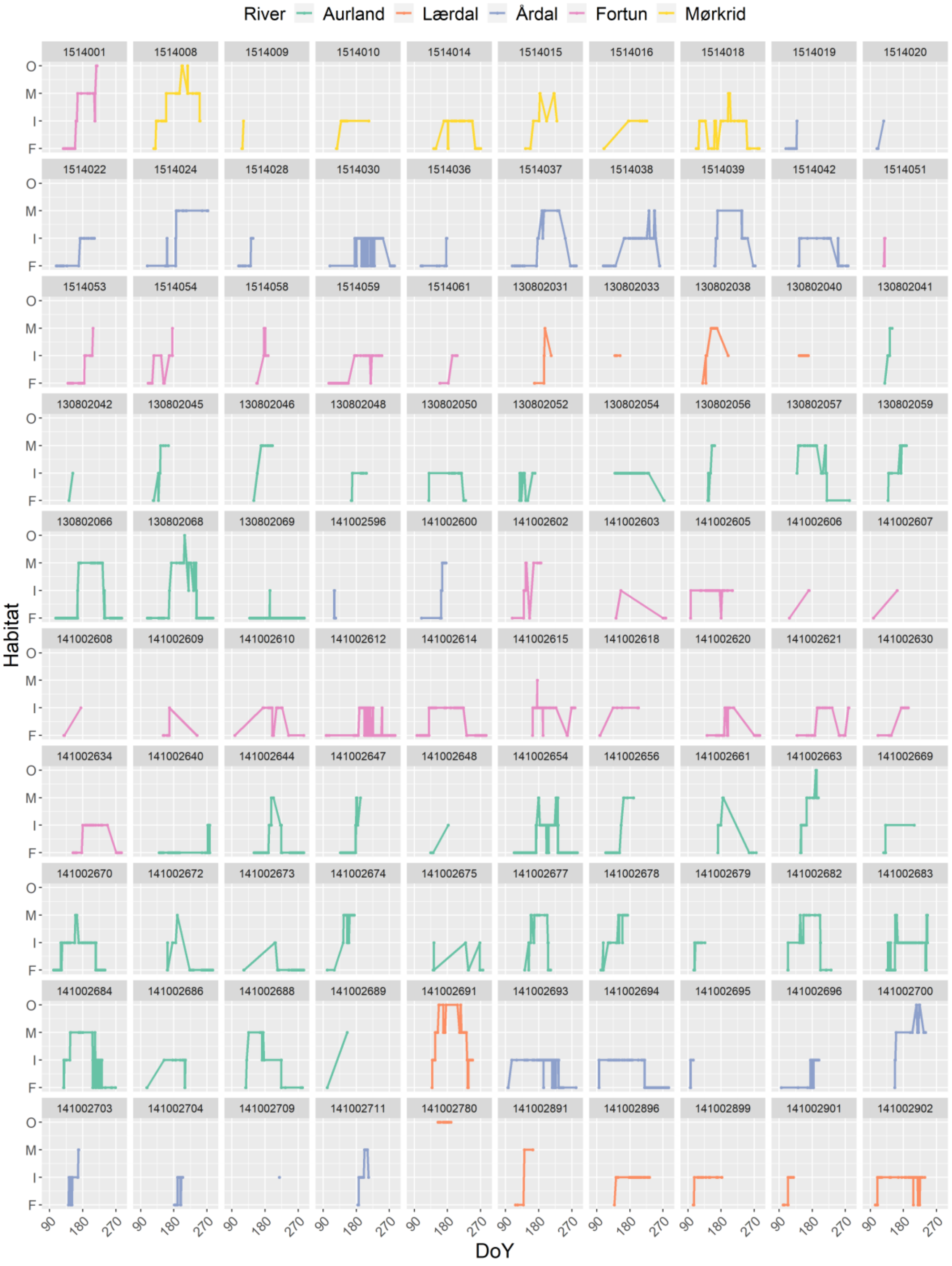

Figure S2: Detection data of Sognefjord *Salmo trutta* veteran migrants (N = 250), sorted by date and habitat zone (F = freshwater, I = inner-fjord, M = mid-fjord, O = outer-fjord). Figures are presented and coloured according to tagging river, data is presented for all sampling years (2012 – 2015).

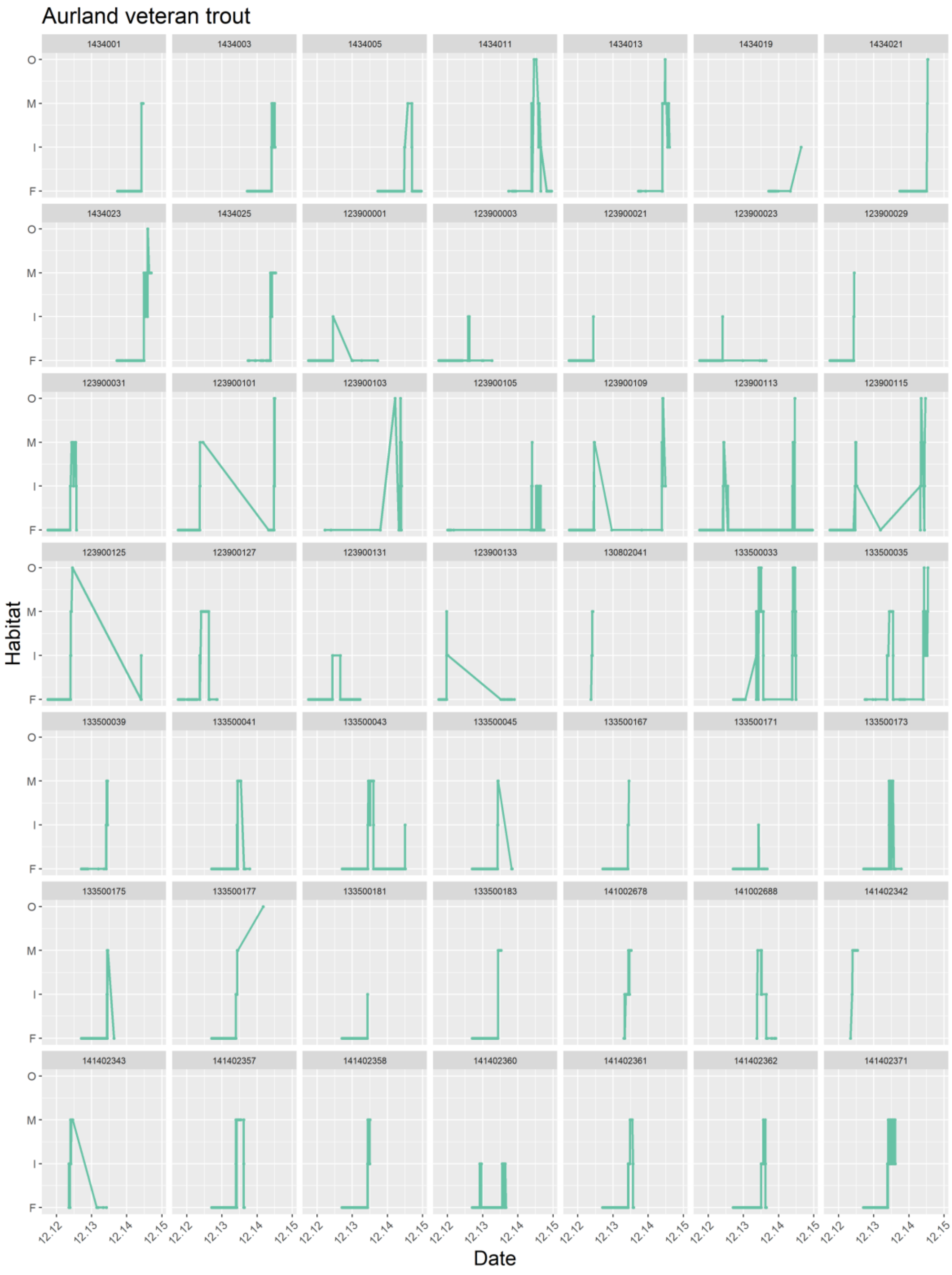

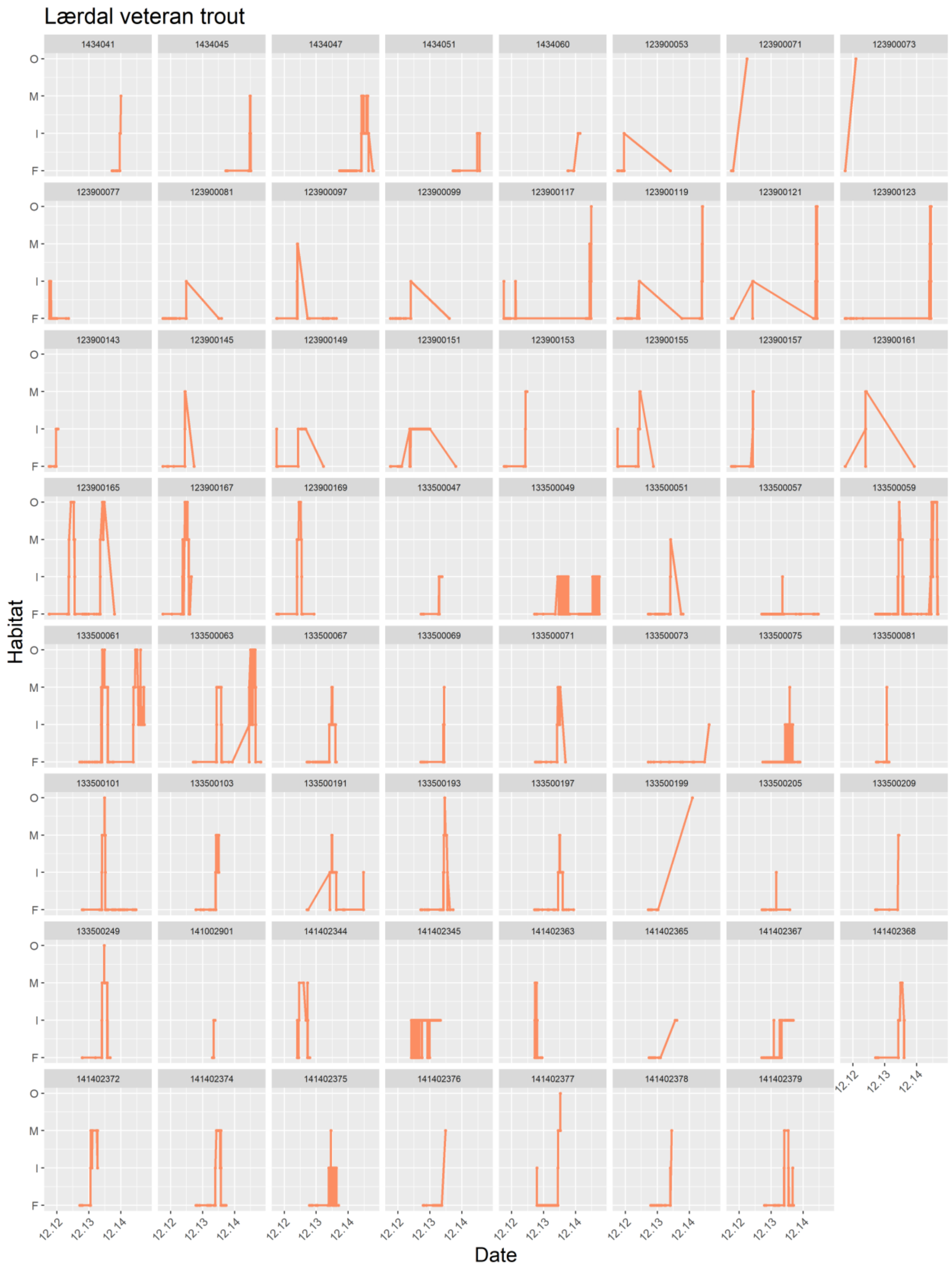
Lærdal veteran trout
1434041
1434045
1434047
1434051
1434060
123900053
123900071
123900073
123900077
123900081
123900097
123900099
123900117
123900119
123900121
123900123
123900143
123900145
123900149
123900151
123900153
123900155
123900157
123900161
123900165
123900167
123900169
133500047
133500049
133500051
133500057
133500059
133500061
133500063
133500067
133500069
133500071
133500073
133500075
133500081
133500101
133500103
133500191
133500193
133500197
133500199
133500205
133500209
133500249
141002901
141402344
141402345
141402363
141402365
141402367
141402368
141402372
141402374
141402375
141402376
141402377
141402378
141402379
O
M
I
F
Habitat
12.12
12.13
12.14
Date

Årdal veteran trout

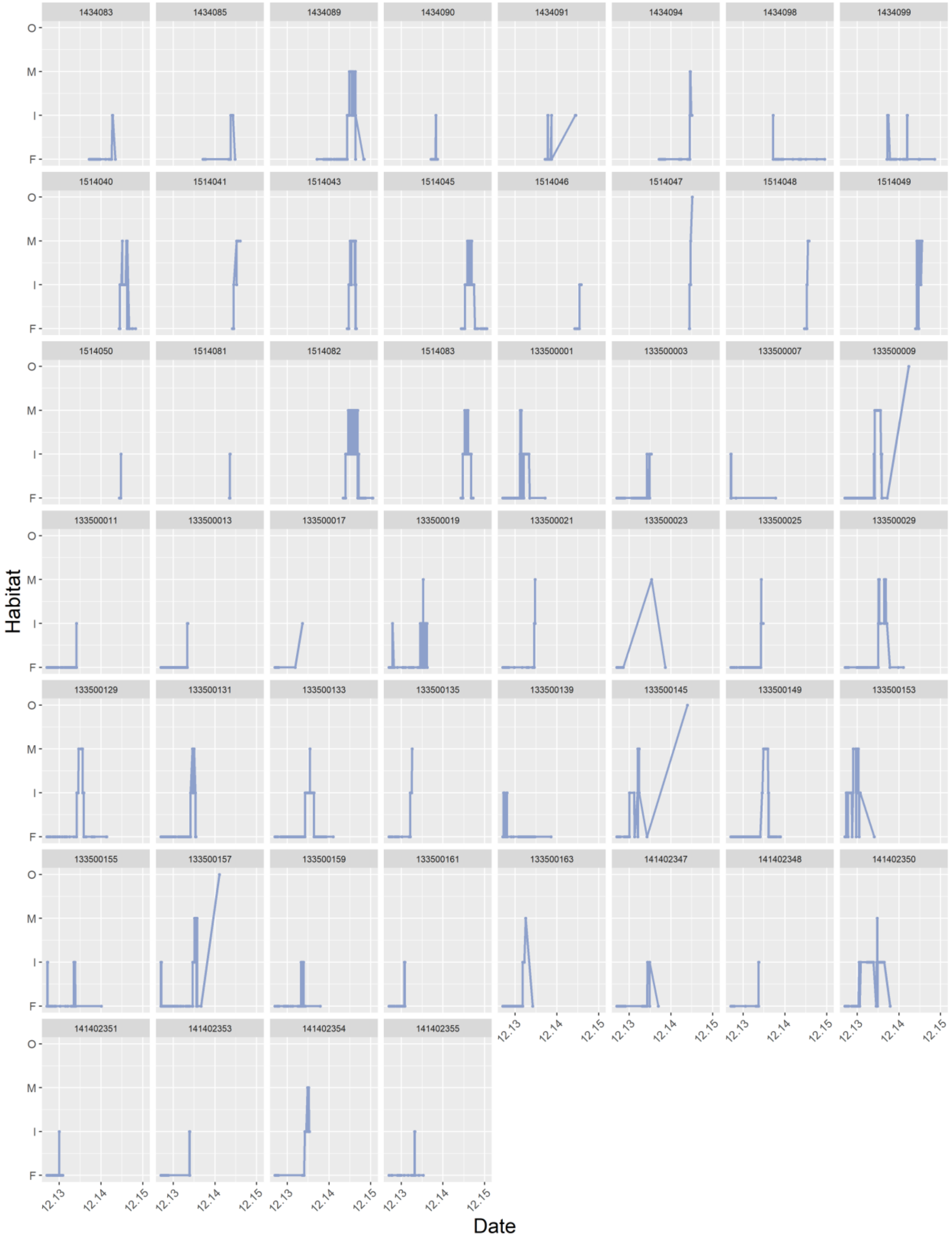

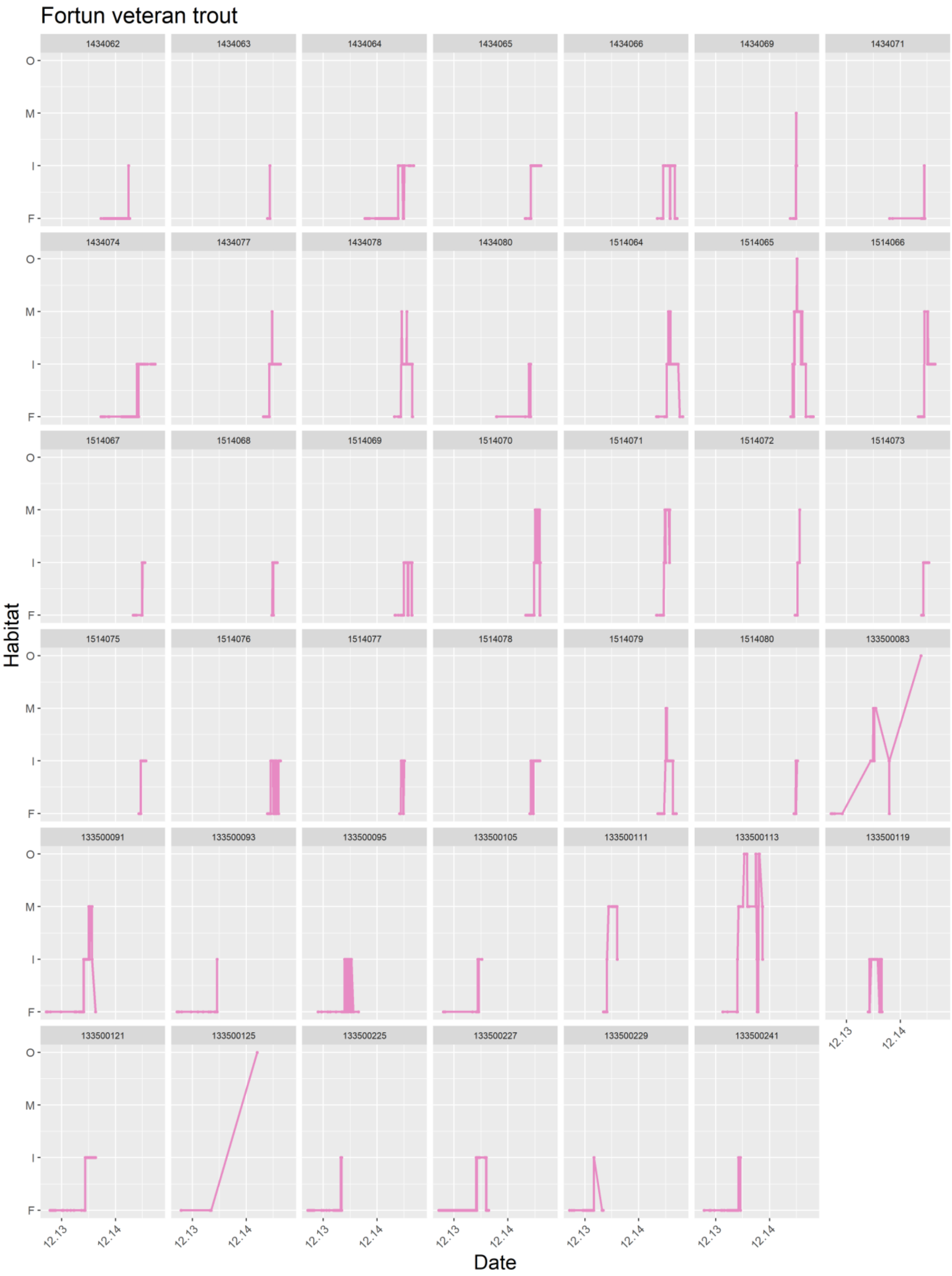
Fortun veteran trout
1434062
1434063
1434064
1434065
1434066
1434069
1434071
1434074
1434077
1434078
1434080
1514064
1514065
1514066
1514067
1514068
1514069
1514070
1514071
1514072
1514073
1514075
1514076
1514077
1514078
1514079
1514080
133500083
133500091
133500093
133500095
133500105
133500111
133500113
133500119
133500121
133500125
133500225
133500227
133500229
133500241
O
M
I
F
Habitat
12.13
12.14
Date

Figure S3: Boxplots showing the mean and inter-quartile range of back calculated estimates of first- and second- year sea specific growth rate. **(a)** Growth rate of Lærdal brown trout sampled during the 'past' (1956 – 1970) and 'present' (2009 – 2014) time periods. **(b)** Contemporary (2012 – 2014) growth rate of acoustically tagged Sognefjord veteran migrant brown trout, grouped according to maximum habitat zone reached. **(c)** Contemporary (2012 – 2014) growth rate of acoustically tagged Sognefjord veteran migrant brown trout, dependent on river of origin. No significant difference in growth rate was observed between time periods **(a)**, nor maximum habitat zone use (one-way ANOVA: $p > 0.05$) **(b)**. A significant difference was observed between rivers for the first-year growth rate at sea (one-way ANOVA: $F = 3.698$, $p = 0.0139$) **(c)**, but no significant difference in second-year growth rate was revealed (one-way ANOVA: $p > 0.05$). derived

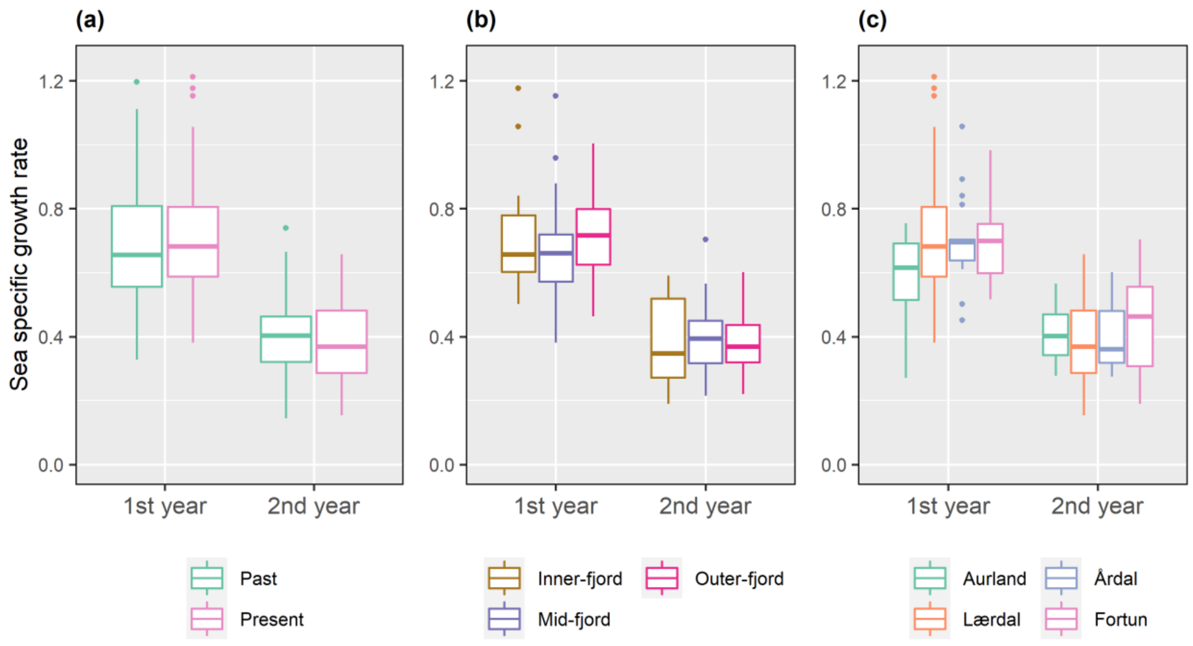

Note: Refer to Table 2 for an overview of the historical and contemporary scale samples from which the 1st / 2nd year specific growth rate at sea were estimated and the number of fish which were implanted with an acoustic tag is also stated.

Table S2: AIC scores of the models generated to describe the migration and habitat use of tagged *Salmo trutta* smolts and veteran migrants in Sognefjorden.

**Model type**	**Response variable**	**Model**	**N par.**	**AIC**	**ΔAICc**
LM	log (Migration extent: **smolts**)	~ River * TL	11	210.80	0.00
		~ River + TL	7	215.16	4.36
		~ TL	3	227.12	16.32
LM	log (Migration extent: **veteran migrants**)	~ River + TL	6	505.97	0.00
		~ River * TL	9	507.41	1.44
		~ TL	3	511.95	5.98
		~ River	5	523.87	17.90
GLM	Probability of migration onset: **veteran migrants**	~ ΔQ * stQ * River * TL	32	18456.31	0.00
		~ ΔQ + stQ * River * TL	17	18567.78	111.47
		~ ΔQ * stQ * River + TL	17	18645.45	189.14
		~ ΔQ * stQ * River	16	18650.68	194.37
		~ ΔQ * stQ + River	7	18763.49	307.17
		~ ΔQ * stQ	4	18777.60	321.29
		~ ΔQ + stQ + River + TL	7	18849.35	393.04
		~ ΔQ + stQ	3	18866.98	410.67
		~ ΔQ * River	8	26881.71	8425.40
		~ ΔQ + River	5	26907.56	8451.25
LM	log (Residence duration: **veteran migrants**)	~ Season * Max zone * Habitat zone	35	2316.46	0.00
		~ Season * Max zone * Habitat zone + TL	36	2318.61	2.15
		~ Season * Max zone *Habitat zone + TL*River	42	2326.86	10.39
		~ Season * Max zone * Habitat zone * TL	65	2330.67	14.20
		~ Season * Habitat zone	16	2332.70	16.24
		~ Season * Habitat zone * TL	30	2334.16	17.69
		~ Season * Habitat zone * River	50	2349.30	32.84
		~ Season * Max zone * Habitat zone * River	105	2350.47	34.01
		~ Season * Habitat zone * River * TL	88	2353.88	37.42
		~ Season * Max zone * River *TL*Habitat zone	179	2423.21	106.74

Note: Where more than ten models were built, only the top ten performing models are given. In all instances the principle of parsimony was adhered to and the model with the lowest AIC score was selected. ΔAICc denotes the difference between a candidate model's AICc value compared to the one with the lowest AICc. Total fish length is indicated by TL; daily mean standardised water discharge, stQ and sequential change in daily mean water discharge, ΔQ.

Table S3: Summary statistics from the models used to describe the migration and habitat use of tagged brown trout smolts and veteran migrants in Sognefjorden.

Response variable	Explanatory variable	Estimate	SE	CI: 2.5%	CI: 97.5%
Migration extent (**smolts**) $R^2 = 0.24$	Intercept	4.436	0.791	2.865	6.007
	Aurland	-0.398	0.968	-2.320	1.524
	Fortun	-0.971	1.105	-3.166	1.224
	Lærdal	1.762	1.135	-0.492	4.017
	Mørkrid	-2.510	2.498	-7.472	2.451
	TL	-0.058	0.050	-0.157	0.041
	Aurland*TL	0.041	0.057	-0.072	0.154
	Fortun*TL	0.093	0.065	-0.036	0.222
	Lærdal*TL	-0.095	0.064	-0.223	0.032
	Mørkrid*TL	0.177	0.130	-0.081	0.436
Migration extent (**veteran migrants**) $R^2 = 0.13$	Intercept	2.739	0.253	2.241	3.238
	Aurland	0.402	0.163	0.080	0.723
	Fortun	0.272	0.176	-0.076	0.620
	Lærdal	-0.088	0.155	-0.394	0.218
	TL	0.024	0.005	0.014	0.035
Probability of migration onset (**veteran migrants**) $R^2 = 0.47$	Intercept	-0.473	0.141	-0.750	-0.196
	QΔ	-1.345	0.153	-1.649	-1.049
	stQ	0.927	0.133	0.667	1.188
	Fortun	0.773	0.258	0.268	1.280
	Lærdal	0.075	0.197	-0.312	0.462
	Årdal	2.650	0.247	2.169	3.137
	TL	0.025	0.003	0.018	0.031
	QΔ* stQ	-1.162	0.226	-1.597	-0.709
	QΔ*Fortun	1.128	0.348	0.484	1.829
	QΔ*Lærdal	-0.397	0.227	-0.842	0.049
	QΔ*Årdal	0.481	0.198	0.097	0.873
	QΔ*Fortun	0.334	0.107	0.127	0.546
	stQ *Lærdal	-0.021	0.092	-0.199	0.160
	stQ *Årdal	0.528	0.105	0.324	0.736
	stQ *TL	0.026	0.003	0.020	0.032
	Fortun*TL	-0.013	0.005	-0.023	-0.003
	Lærdal*TL	-0.001	0.004	-0.009	0.008
	Årdal*TL	-0.055	0.006	-0.066	-0.044
	QΔ*stQ*Fortun	0.829	0.487	-0.049	1.814
	QΔ*stQ*Lærdal	-0.433	0.336	-1.090	0.228
	QΔ*stQ*Årdal	0.386	0.276	-0.153	0.936
Residence duration (**veteran migrants**) $R^2 = 0.37$	Intercept	4.182	0.569	3.066	5.298
	Spring/summer	-1.498	0.596	-2.669	-0.328
	Autumn	0.237	0.585	-0.912	1.386
	Winter-early	-1.511	0.985	-3.444	0.422
	Max zone: Mid-fjord	-1.722	0.763	-3.219	-0.224
	Max zone: Outer-fjord	0.198	0.763	-1.299	1.696

Inner-fjord	-1.677	0.763	-3.175	-0.180
Mid-fjord	-3.302	1.246	-5.747	-0.856
Outer-fjord	-0.721	1.246	-3.167	1.724
Spring*Max zone: Mid-fjord	1.618	0.805	0.038	3.199
Autumn*Max zone: Mid-fjord	1.595	0.783	0.057	3.133
Winter-early*Max zone: Mid-fjord	1.334	1.246	-1.111	3.780
Spring*Max zone: Outer-fjord	-0.065	0.831	-1.696	1.566
Autumn*Max zone: Outer-fjord	-0.122	0.791	-1.675	1.430
Winter-early*Max zone: Outer-fjord	1.908	2.260	-2.528	6.345
Spring*Inner-fjord	2.126	0.801	0.554	3.699
Autumn*Inner-fjord	-0.216	0.826	-1.837	1.405
Winter-early*Inner-fjord	1.263	1.246	-1.183	3.708
Spring*Mid-fjord	3.471	1.288	0.942	6.000
Autumn*Mid-fjord	1.230	1.311	-1.344	3.805
Winter-early*Mid-fjord	-0.643	1.246	-3.089	1.802
Spring*Outer-fjord	0.521	1.296	-2.023	3.065
Autumn*Outer-fjord	-0.813	1.417	-3.594	1.968
Max zone: Mid-fjord*Inner-fjord	3.001	1.028	0.983	5.018
Max zone: Outer-fjord*Inner-fjord	0.450	1.219	-1.944	2.844
Max zone: Mid-fjord*Mid-fjord	3.702	1.461	0.835	6.569
Spring*Max zone: Mid-fjord*Inner-fjord	-3.326	1.081	-5.448	-1.205
Autumn*Max zone: Mid-fjord*Inner-fjord	-3.098	1.105	-5.267	-0.929
Winter-early*Max zone: Mid-fjord*Inner-fjord	-3.411	1.667	-6.684	-0.138
Spring*Max zone: Outer-fjord*Inner-fjord	-1.935	1.286	-4.460	0.590
Autumn*Max zone: Outer-fjord*Inner-fjord	-1.242	1.311	-3.816	1.332
Winter-early*Max zone: Outer-fjord*Inner-fjord	-4.575	2.762	-9.998	0.847
Spring*Max zone: Mid-fjord*Mid-fjord	-3.363	1.515	-6.337	-0.389
Autumn*Max zone: Mid-fjord*Mid-fjord	-4.212	1.547	-7.250	-1.174

Note: Models were selected according to AIC (Table S2). SE denotes the standard error for the estimates, and CI the confidence interval. R^2 gives the adjusted r-squared value of the model. Total fish length is denoted by TL; daily mean standardised water discharge, stQ and sequential change in daily mean water discharge, ΔQ. The seasonal periods were defined accordingly: winter-late = week of year (WoY) 1 – 12, spring/summer = WoY 13 – 26, autumn = WoY 27 – 40 and winter-early = WoY 41 – 52.

Figure S4: Box plots showing median, interquartile range and 5th/95th percentiles of migrant (leave fresh water) and resident (remain in freshwater) immature Sognefjord brown trout. Migratory individuals were significantly larger (one-way ANOVA: F= 8.87, p= 0.004) and in poorer condition (one-way ANOVA: F= 5.50, p= 0.021), than fish that remained.

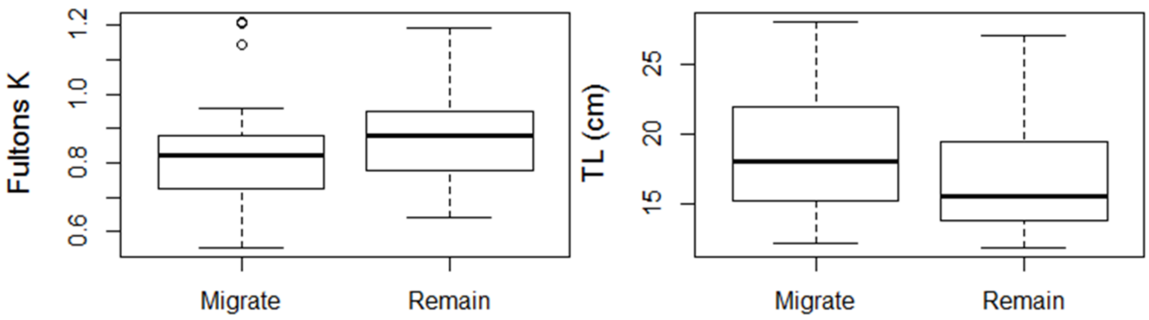

Table S4: Summary statistics of residence duration (in weeks) within each given habitat zone of tagged Sognefjord brown trout smolt migrants over a six-month period.

River	Mean residence duration (weeks) ± SD (N)			
	**Freshwater**	**Inner-fjord**	**Mid-fjord**	**Outer-fjord**
Aurland	6.36 ± 6.3 (37)	4.44 ± 4.8 (36)	4.34 ± 3.2 (24)	0.87 ± 0.8 (2)
Lærdal	1.62 ± 1.3 (8)	6.05 ± 6.1 (9)	4.32 ± 1.9 (4)	6.25 ± 1.6 (2)
Årdal	5.73 ± 4.5 (21)	4.74 ± 6.2 (22)	5.44 ± 4.9 (8)	2.31 ± NA (1)
Fortun	7.22 ± 5.5 (23)	6.63 ± 5.8 (23)	2.00 ± 3.0 (6)	0.31 ± NA (1)
Mørkrid	4.53 ± 3.9 (7)	7.66 ± 4.9 (7)	5.05 ± 5.1 (3)	2.25 ± NA (1)
**Total mean ± SD (N)**	**5.09 ± 4.3 (96)**	**5.09 ± 5.6 (97)**	**4.23 ± 3.6 (45)**	**2.40 ± 1.2 (7)**

Note: N denotes number, SD standard deviation. The values were derived from acoustic telemetry data, conducted during a six-month period over three years. (WoY: 13 – 40, 2013 – 2015).

Figure S5: Total residence duration (in weeks) of individual tagged brown trout smolt migrants (N = 100) during a six-month period (WoY: 13 – 40, 2013 – 2015) for each given habitat zone of Sognefjorden (I = inner-fjord, M = mid-fjord, O = outer-fjord) coloured according to sampled natal river (F = freshwater).

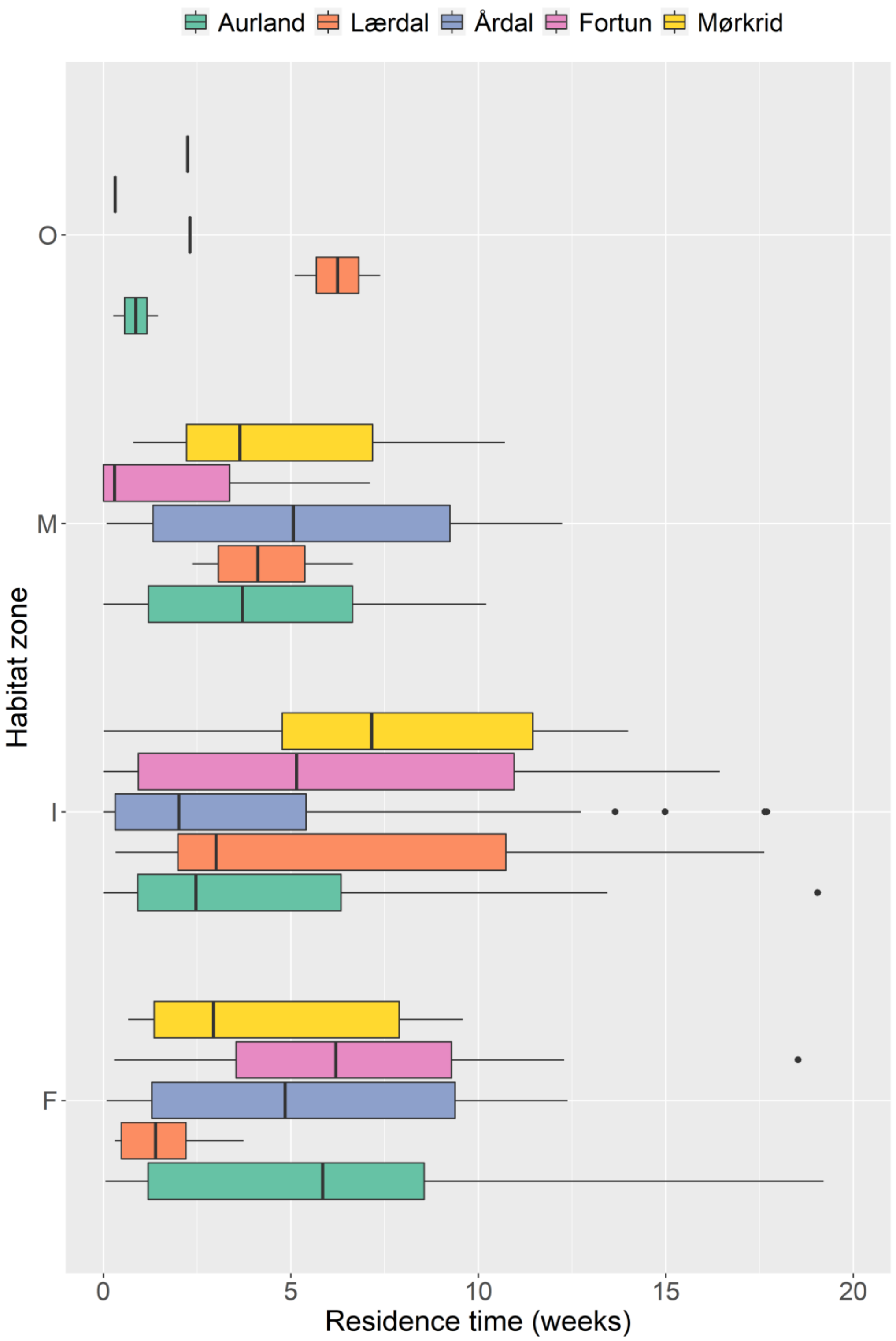

Table S5: Summarised seasonal depth use of veteran migrant brown trout within each given habitat zone of Sognefjorden.

Mean depth ± SD (N)				
**Season**	**River**	**Inner-fjord**	**Mid-fjord**	**Outer-fjord**
Spring/summer	Aurland	-2.94 ± 7.1 (25)	-7.21 ± 11.2 (23)	-2.25 ± 4.7 (9)
	Lærdal	-5.56 ± 7.4 (33)	-5.15 ± 8.4 (27)	-3.58 ± 3.7 (12)
	Årdal	-21.43 ± 14.1 (18)	-5.53 ± 8.5 (10)	
	Fortun	-2.57 ± 2.3 (15)	-11.94 ± 14.1 (6)	-32.00 ± NA (1)
**Season mean ± SD (N)**		**-8.12 ± 7.7 (91)**	**-7.45 ± 10.8 (66)**	**-12.61 ± 4.2 (22)**
Autumn	Aurland	-5.21 ± 2.2 (6)	-4.82 ± 1.6 (5)	
	Lærdal	-7.93 ± 9.0 (18)	-12.34 ± 10.2 (7)	-26.33 ± 6.0 (3)
	Årdal	-11.86 ± 14.3 (13)	-26.59 ± 11.6 (5)	
	Fortun	-4.44 ± 5.3 (8)	-11.36 ± 10.9 (3)	-32.00 ± 0.0 (1)
**Season mean ± SD (N)**		**-7.36 ± 7.7 (45)**	**-13.77 ± 8.6 (20)**	**-29.16 ± 3.0 (4)**
Winter	Aurland	-8.19 ± 10.8 (1)	-6.00 ± NA (1)	-6.75 ± 9.2 (1)
	Lærdal	-13.47 ± 9.3 (7)	-0.25 ± NA (1)	
	Årdal	-31.80 ± 2.5 (8)	-25.19 ± 8.9 (2)	-12.00 ± NA (1)
	Fortun	-32.00 ± 0.0 (1)	-32.00 ± 0.0 (1)	-18.98 ± 25.8 (2)
**Season mean ± SD (N)**		**-21.36 ± 5.6 (17)**	**-15.86 ± 4.4 (5)**	**-12.57 ± 17.5 (5)**

Note: N denotes number, SD standard deviation. The seasonal periods were defined accordingly: spring/summer = WoY 13 – 26, autumn = WoY 27 – 40, winter = WoY 1 – 12 and 41 – 52. Depth data was retrieved from individuals tagged with pressure sensors tags (ADT-9-LONG/ADT-13-STAT, n = 114), all depths greater than 50 m were excluded from analysis.

Figure S6: Mean annual depth use of individual veteran migrant brown trout. Depth data was retrieved from individuals tagged with pressure sensors (ADT-9-LONG/ADT-13-STAT, n = 114), all depths greater than 50 m were excluded from analysis. Values are grouped according to season (spring/summer = WoY 13 – 26, autumn = WoY 27 – 40, winter = WoY 1 – 12 and 41 – 52), separated by given habitat zonation of Sognefjorden and coloured according to sampling river.

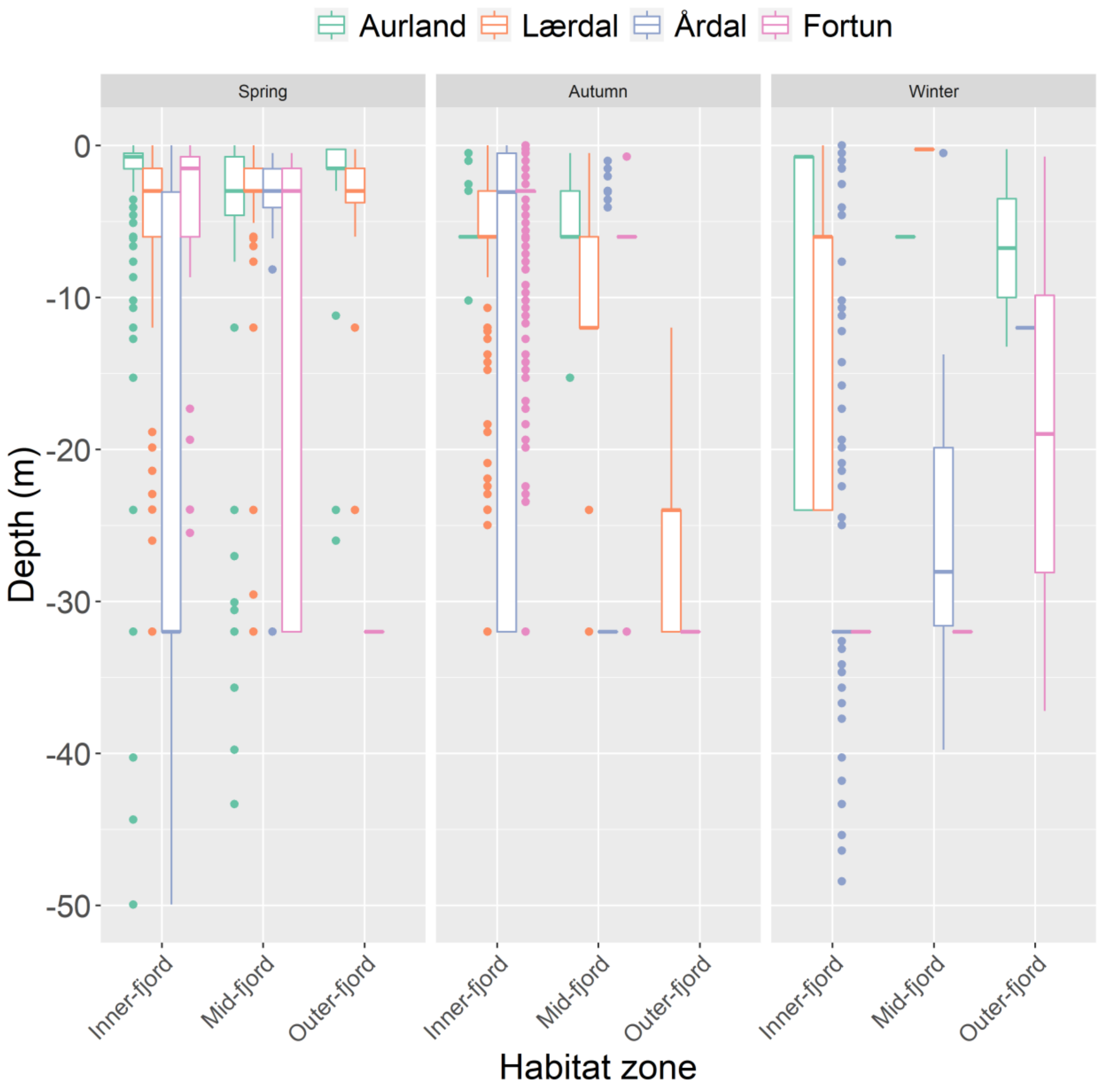

Table S6: AIC scores of candidate conditional Arnason-Schwarz (CAS) mark-recapture models to estimate rates of survival (**S**), recapture (detection) (**p**) and transition (migration) (**Psi**) of tagged Sognefjord brown trout smolts.

Model N.	Model	QAICc	Δ QAICc	Model Likelihood	N. Par.
1	{(**S*Zone/Rvr:** F/I+M+O*Rvr)<br>(**p*Time/Zone:** F/I/M*t, O(·))<br>(**Psi*Zone/Rvr/Ssn:** FI*Rvr,<br>FM/IF/IM/MF/MI/MO/OM/FO+IO/OF+OI*Ssn)}	2661.1	0.0	1.00	73
2	{(**S*Zone/Rvr:** F/I+M+O*Rvr)<br>(**p*Time/Zone:** F/I/M*t, O(·))<br>(**Psi*Zone/Rvr/Ssn:** FI*Rvr,<br>FM/IF/IM/MF/MI/MO/OM/FO+IO/OF+OI*Ssn<br>(Spr = WoY: 13 – 24, Aut = WoY: 25 – 40))}	2668.3	7.2	0.03	73
3	{(**S*Zone/Rvr:** F/I+M+O*Rvr)<br>(**p*Time/Zone:** F/I/M*t, O*Ssn)<br>(**Psi*Zone/Rvr/Ssn:** FI*Rvr,<br>FM/IF/IM/MF/MI/MO/OM/FO+IO/OF+OI*Ssn)}	2670.6	9.5	0.01	74
4	{(**S*Zone/Rvr:** F/I+M+O*Rvr)<br>(**p*Time/Zone:** F/I/M*t, O(·))<br>(**Psi*Zone/Rvr/Ssn:** FI*Rvr, MO*Rvr*Ssn,<br>FM/IF/IM/MF/MI/MO/OM/FO+IO/OF+OI*Ssn)}	2671.3	10.2	0.01	81
5	{(**S*Zone/Rvr:** F/I+M+O*Rvr)<br>(**p*Time/Zone:** F/I/M*t, O(·))<br>(**Psi*Zone/Rvr:** FI*Rvr,<br>FM/IF/IM/MF/MI/MO/OM/FO+IO/OF+OI(·)}	2679.8	18.7	0.00	64
6	{(**S*Zone/Rvr:** F/I+M+O*Rvr)<br>(**p*Time/Zone:** F/I/M*t, O(·))<br>(**Psi*Zone/Rvr/Ssn:** FI*Rvr,<br>FM/IF/IM/MF/MI/FO+IO+MO/OF+OI+OM*Ssn)}	2688.2	27.1	0.00	71
7	{(**S*Zone/Rvr:** F/I+M+O*Rvr)<br>(**p*Zone/Ssn:** F/I/M/O*Ssn)<br>(**Psi*Zone/Rvr:** FI*Rvr,<br>FM/IF/IM/MF/MI/MO/OM/FO+IO/OF+OI(·)}	2740.7	79.6	0.00	41
8	{(**S*Zone/Rvr:** F/I+M+O*Rvr)<br>(**p*Time/Zone:** F/I/M/O*t)<br>(**Psi*Zone/Rvr/Ssn:** FI*Rvr,<br>FM/IF/IM/MF/MI/MO/OM/FO+IO/OF+OI(·)}	2752.0	90.9	0.00	76
9	{(**S*Zone/Rvr:** F/I+M+O*Rvr)<br>(**p*Time/Zone:** F/I/M*t, O(·))<br>(**Psi*Zone/Rvr/Ssn:** FI*Rvr,<br>FM/IF/IM/MF/MI/MO/OM*Ssn, FO+IO/OF+OI(·))}	2778.1	117.0	0.00	71

10	{(**S*Zone/Rvr:** F/I+M+O*Rvr)<br>(**p*Time/Zone/Ssn:** F/I/M*t, O*Ssn)<br>(**Psi*Zone/Rvr/Ssn:** FI*Rvr, FM/IF/IM/MF/MI/MO/OM*Ssn, FO+IO/OF+OI(·))}	2778.8	117.7	0.00	74
11	{(**S*Zone/Rvr:** F/I+M+O*Rvr)<br>(**p*Zone:** F/I/M/O(·))<br>(**Psi*Zone/Rvr:** FI*Rvr, FM/IF/IM/MF/MI/MO/OM/FO+IO/OF+OI(·)}	2820.4	159.3	0	28
12	{(**S*Zone/Rvr:** F/I+M+O*Rvr (Aurland/Årdal/Mørkrid), Rvr (Fortun/Lærdal (·))<br>(**p*Zone:** F/I/M/O(·))<br>(**Psi*Zone/Rvr:** FI*Rvr, FM/IF/IM/MF/MI/MO/OM/FO+IO/OF+OI(·)}	2823.7	162.6	0	26
13	{(**S*Rvr:** Rvr(.))<br>(**p*Zone:** F/I/M/O(·))<br>(**Psi*Zone/Rvr:** FI*Rvr, FM/IF/IM/MF/MI/MO/OM/FO+IO/OF+OI(·)}	2826.8	165.6	0	23
14	{(**S*Yr*Zone:** F/I/M/O*Yr)<br>(**p*Zone:** F/I/M/O(·))<br>(**Psi*Zone/Rvr:** FI*Rvr, FM/IF/IM/MF/MI/MO/OM/FO+IO/OF+OI(·)}	2842.0	180.9	0	28
15	{(**S*Yr*Zone:** F/I/M/O*Yr)<br>(**p*Zone:** F/I/M/O(·))<br>(**Psi*Zone/Rvr:** FI*Rvr, IF*Rvr, FM/IM/MF/MI/MO/OM/FO+IO/OF+OI(·)}	2843.2	182.0	0	32

Note: Only model structures of the top performing 15 models are presented.

QAICc = -2log likelihood / $\hat{c}$ + 2*Npar* + 2*Npar* (*Npar* + 1) / (*Ness* – *Npar* – 1)

Where: $\hat{c}$ = 1.27, *Npar* = number of model parameters, *Ness* = the effective sample size.

ΔQAICc is the difference between a candidate model's QAICc compared to the one with the lowest QAICc.

Temporal model predictors were defined as: time (t), year (Yr) and season (SSn). Seasons were defined accordingly, spring/summer (Spr) = WoY: 13 – 26 and autumn (Aut) = WoY: 27 – 40, except for model number 2 (Spr = WoY: 13 – 24, Aut = WoY: 25 – 40). Four spatial states were defined as the habitat zones: F = freshwater, I= inner-fjord, M = mid-fjord, O = outer-fjord, and individuals were grouped by natal river (Rvr).

"/" indicates predictor separation, "+" indicates grouped as a single predictor, "*" indicates interactive effect of predictor and "(·)" indicates that predictors were held constant.

Table S7: Logit parameter estimates for the selected conditional Arnason-Schwarz (CAS) mark-recapture model (See Table S6), to estimate rates of survival (**S**), recapture (detection) (**p**) and transition (migration) (**Psi**) of tagged Sognefjord brown trout smolts.

Par. N	Par.	River	Zone / State	Occasion (t) / Ssn	Estimate	SE	LCI	UCI
1	S	Fortun	F	All (·)	1.571	0.297	0.989	2.153
2	S	Fortun	I+M+O	All (·)	1.164	0.086	0.995	1.332
3	S	Mørkrid	F	All (·)	1.218	0.154	0.916	1.520
4	S	Mørkrid	I+M+O	All (·)	1.428	0.346	0.749	2.106
5	S	Årdal	F	All (·)	1.194	0.095	1.007	1.382
6	S	Årdal	I+M+O	All (·)	1.261	0.082	1.101	1.421
7	S	Lærdal	F	All (·)	7.854	0.311	7.245	8.463
8	S	Lærdal	I+M+O	All (·)	1.065	0.120	0.830	1.300
9	S	Aurland	F	All (·)	1.571	0.077	1.421	1.721
10	S	Aurland	I+M+O	All (·)	1.250	0.082	1.090	1.410
11	p	All (·)	F	1-2	-0.468	0.116	-0.695	-0.240
12	p	All (·)	F	2-3	-0.088	0.118	-0.318	0.143
13	p	All (·)	F	3-4	0.537	0.173	0.198	0.877
14	p	All (·)	F	4-5	-5.549	0.186	-5.913	-5.185
15	p	All (·)	F	5-6	-4.839	0.495	-5.810	-3.869
16	p	All (·)	F	6-7	-4.712	0.384	-5.466	-3.959
17	p	All (·)	F	7-8	-5.003	0.293	-5.577	-4.429
18	p	All (·)	F	8-9	-5.106	0.266	-5.628	-4.585
19	p	All (·)	F	9-10	1.832	0.241	1.358	2.305
20	p	All (·)	F	10-11	-4.084	0.218	-4.512	-3.657
21	p	All (·)	F	11-12	-5.232	0.244	-5.711	-4.753
22	p	All (·)	F	12-13	-4.739	3.539	-11.674	2.197
23	p	All (·)	F	13-14	-4.712	0.395	-5.487	-3.938
24	p	All (·)	I	1-2	-2.793	0.452	-3.679	-1.907
25	p	All (·)	I	2-3	2.689	0.392	1.921	3.456
26	p	All (·)	I	3-4	-4.712	0.460	-5.614	-3.810
27	p	All (·)	I	4-5	-5.329	0.338	-5.992	-4.665
28	p	All (·)	I	5-6	2.545	0.288	1.980	3.110
29	p	All (·)	I	6-7	-4.187	0.282	-4.739	-3.635
30	p	All (·)	I	7-8	-5.310	0.231	-5.763	-4.857
31	p	All (·)	I	8-9	1.571	0.344	0.896	2.246
32	p	All (·)	I	9-10	-4.712	0.768	-6.218	-3.206
33	p	All (·)	I	10-11	-3.827	0.481	-4.770	-2.885
34	p	All (·)	I	11-12	3.309	0.348	2.626	3.992
35	p	All (·)	I	12-13	-13.649	0.281	-14.199	-13.099
36	p	All (·)	I	13-14	4.000	0.297	3.418	4.581
37	p	All (·)	M	1-2	-7.854	1.098	-10.007	-5.701
38	p	All (·)	M	2-3	-1.571	0.767	-3.075	-0.067
39	p	All (·)	M	3-4	-3.193	0.638	-4.444	-1.941
40	p	All (·)	M	4-5	0.835	0.406	0.040	1.631

41	p	All (·)	M	5-6	1.571	0.449	0.690	2.452
42	p	All (·)	M	6-7	0.751	0.310	0.143	1.360
43	p	All (·)	M	7-8	1.796	1.639	-1.418	5.009
44	p	All (·)	M	8-9	1.571	1.344	-1.064	4.206
45	p	All (·)	M	9-10	-4.712	1.885	-8.408	-1.017
46	p	All (·)	M	10-11	7.854	1.145	5.609	10.099
47	p	All (·)	M	11-12	-11.209	7.971	-26.831	4.414
48	p	All (·)	M	12-13	-9.786	1.953	-13.614	-5.959
49	p	All (·)	M	13-14	-12.016	2.441	-16.800	-7.233
50	p	All (·)	O	All (·)	-1.859	0.050	-1.958	-1.760
51	Psi	Fortun	F to I	All (·)	4.012	0.088	3.840	4.184
52	Psi	Mørkrid	F to I	All (·)	-2.374	0.173	-2.714	-2.035
53	Psi	Årdal	F to I	All (·)	-0.750	0.118	-0.982	-0.519
54	Psi	Lærdal	F to I	All (·)	-2.903	0.251	-3.395	-2.411
55	Psi	Aurland	F to I	All (·)	-1.171	0.057	-1.282	-1.059
56	Psi	All (·)	F to M	Spring/summer	-1.861	0.057	-1.973	-1.748
57	Psi	All (·)	F to M	Autumn	-1.447	0.073	-1.589	-1.304
58	Psi	All (·)	F to O+I to O	Spring/summer	5.454	0.064	5.328	5.580
59	Psi	All (·)	F to O+I to O	Autumn	-0.938	0.079	-1.093	-0.782
60	Psi	All (·)	I to F	Spring/summer	4.365	0.111	4.148	4.582
61	Psi	All (·)	I to F	Autumn	-0.630	0.107	-0.841	-0.420
62	Psi	All (·)	I to M	Spring/summer	-7.019	0.122	-7.258	-6.780
63	Psi	All (·)	I to M	Autumn	-1.849	0.093	-2.032	-1.666
64	Psi	All (·)	M to F	Spring/summer	-1.571	0.278	-2.116	-1.026
65	Psi	All (·)	M to F	Autumn	-6.821	0.240	-7.291	-6.351
66	Psi	All (·)	M to I	Spring/summer	4.236	0.187	3.870	4.602
67	Psi	All (·)	M to I	Autumn	-2.165	0.234	-2.624	-1.705
68	Psi	All (·)	M to O	Spring/summer	-0.503	0.243	-0.979	-0.026
69	Psi	All (·)	M to O	Autumn	-9.067	0.242	-9.541	-8.593
70	Psi	All (·)	O to F+O to I	Spring/summer	4.070	0.065	3.942	4.197
71	Psi	All (·)	O to F+O to I	Autumn	5.324	0.082	5.164	5.484
72	Psi	All (·)	O to M	Spring/summer	-1.420	0.084	-1.585	-1.255
73	Psi	All (·)	O to M	Autumn	-1.292	0.090	-1.468	-1.116

Note: SE denotes the standard error for the parameter estimates. UCI/LCI denotes the upper and lower 95% confidence intervals for each parameter estimate.

Temporal model effects were defined as: time (t) and season (SSn). Seasons were defined accordingly, spring/summer (Spr) = WoY: 13 – 26 and autumn (Aut) = WoY: 27 – 40. Four spatial states were defined as the habitat zones: F = freshwater, I= inner-fjord, M = mid-fjord, O = outer-fjord, and individuals were grouped by natal river (Rvr).

"/" indicates predictor separation, "+" indicates grouped as a single predictor, "*" indicates interactive effect of predictor and "(·)" indicates that predictors were held constant.

Table S8: AIC scores of candidate conditional Arnason-Schwarz (CAS) mark-recapture models to estimate rates of survival (**S**), recapture (detection) (**p**) and transition (migration) (**Psi**) of tagged Sognefjord veteran migrant brown trout.

Model N.	Model	QAICc	ΔQAICc	Model Likelihood	N. Par.
1	(**S*Zone/Rvr/Ssn:** F/I*Rvr, I/M+O*Win)	5431.88	0.00	1.0	58
2	(**S*Zone/Rvr/Ssn:** F/I/O*Rvr, I/M+O*Win)	5432.74	0.86	0.7	61
3	(**S*Zone/Rvr/Ssn:** F*Rvr, I/M+O*Win)	5433.48	1.60	0.5	55
4	(**S*Zone/Rvr/Ssn:** F/I/O*Rvr, I+F/M+O*Win)	5434.97	3.09	0.2	61
5	(**S*Zone/Rvr/Ssn:** F*Rvr, I*Aut, I/M+O*Win)	5435.00	3.12	0.2	56
6	(**S*Zone/Rvr/Ssn:** F*Rvr, F+I/M+O*Win)	5435.42	3.54	0.2	55
7	(**S*Zone/Rvr/Ssn:** F*Rvr, I+M+O*Win)	5435.85	3.97	0.1	54
8	(**S*Zone/Rvr/Ssn:** F*Rvr, I/M+O*Aut, I/M+O*Win)	5437.11	5.23	0.1	57
9	(**S*Zone/Rvr/Ssn:** F/I/M*Rvr, I/M+O*Win)	5437.30	5.42	0.1	61
10	(**S*Zone/Rvr/Ssn:** F/I/M/O*Rvr, I/M+O*Win)	5438.59	6.71	0.0	64
11	(**S*Zone/Rvr/Ssn:** F*Rvr, F/I+M+O*Win)	5439.16	7.28	0.0	55
12	(**S*Zone/Rvr/Ssn:** F/I/M/O*Rvr, F+I/M+O*Win)	5440.82	8.94	0.0	64
13	(**S*Zone/Rvr/Ssn:** F*Rvr, I/M/O*Aut, I/M/O*Win)	5441.27	9.38	0.0	59
14	(**S*Zone/Rvr/Ssn:** F*Rvr, F+I+M+O*Win)	5441.72	9.84	0.0	54
15	(**S*Zone/Rvr/Ssn:** F*Rvr, F+M+O/I*Win)	5442.86	10.98	0.0	55

Note: Only model structures of the top performing 15 models are presented. The accompanying (p) and (Psi) model structures were: (**p*Zone/Rvr/Ssn/Yr:** F*Rr*Yr, M*Win) (**Psi*Zone/Rvr/Ssn:** FI*Rvr*Spr, FM/IF/IM/MF/MI/MO/OM/FO+OF/IO+OI*Spr, FI/FM/IF/IM/MF/MI/MO/OM/FO+OF/IO+OI*Aut, FO+OF/FI+IF/FM+MF/IM+MI/IO+OI+MO+OM*Win)

QAICc = -2log likelihood / $\hat{c}$ + 2*Npar* + 2*Npar* (*Npar* + 1) / (*Ness* – *Npar* – 1)

Where: $\hat{c}$ = 1.45, *Npar* = number of model parameters, *Ness* = the effective sample size.

ΔQAICc is the difference between a candidate model's QAICc compared to the one with the lowest QAICc

Temporal model effects were defined as: year (Yr) and season (SSn). Seasons were defined accordingly: spring/summer (Spr) = WoY: 13 – 26, autumn (Aut) = WoY: 27 – 40 and winter (Win) WoY = 1 – 12 and 41 – 52. Four spatial states were defined as the habitat zones: F = freshwater, I= inner-fjord, M = mid-fjord, O = outer-fjord, and individuals were grouped by river of orgin (Rvr).

"/" indicates predictor separation, "+" indicates grouped as a single predictor, "*" indicates interactive effect of predictor and "(·)" indicates that predictors were held constant.

Table S9: Logit parameter estimates for the selected conditional Arnason-Schwarz (CAS) mark-recapture model (See Table S8), to estimate rates of survival (**S**), recapture (detection) (**p**) and transition (migration) (**Psi**) of tagged Sognefjord veteran migrant brown trout.

Par. N	Par.	River	Zone / State	Occasion / Season	Year	Est.	SE	LCI	UCI
1	S	Aurland	F	All (·)	All (·)	1.571	0.111	1.353	1.788
2	S	Fortun	F	All (·)	All (·)	1.571	0.120	1.335	1.806
3	S	Lærdal	F	All (·)	All (·)	1.662	0.024	1.614	1.709
4	S	Årdal	F	All (·)	All (·)	1.501	0.033	1.436	1.566
5	S	Aurland	I	Spr + Aut	All (·)	1.722	0.165	1.399	2.045
6	S	Fortun	I	Spr + Aut	All (·)	1.072	0.071	0.933	1.210
7	S	Lærdal	I	Spr + Aut	All (·)	1.351	0.075	1.204	1.499
8	S	Årdal	I	Spr + Aut	All (·)	1.145	0.082	0.984	1.306
9	S	All (·)	M	Spr + Aut	All (·)	1.511	0.179	1.161	1.862
13	S	Aurland	O	Spr + Aut	All (·)	-4.712	0.244	-5.191	-4.234
10	S	Fortun	O	Spr + Aut	All (·)	1.178	0.081	1.019	1.337
11	S	Lærdal	O	Spr + Aut	All (·)	1.571	0.072	1.430	1.712
12	S	Årdal	O	Spr + Aut	All (·)	1.571	0.194	1.190	1.952
14	S	All (·)	I	Winter	All (·)	1.571	0.081	1.412	1.730
15	S	All (·)	M + O	Winter	All (·)	1.368	0.035	1.299	1.437
16	p	Aurland	F	All (·)	2012	1.571	0.255	1.072	2.070
17	p	Aurland	F	All (·)	2013	1.916	0.122	1.676	2.155
18	p	Aurland	F	All (·)	2014	0.774	0.103	0.571	0.976
19	p	Aurland	F	All (·)	2015	-5.132	0.172	-5.468	-4.796
20	p	Fortun	F	All (·)	2012	-26.28	3177.66	-6254.51	6201.94
21	p	Fortun	F	All (·)	2013	0.361	0.118	0.130	0.592
22	p	Fortun	F	All (·)	2014	2.232	0.120	1.998	2.466
23	p	Fortun	F	All (·)	2015	0.931	0.149	0.638	1.223
24	p	Lærdal	F	All (·)	2012	-3.660	0.173	-4.000	-3.320
25	p	Lærdal	F	All (·)	2013	0.582	0.088	0.410	0.754
26	p	Lærdal	F	All (·)	2014	0.613	0.105	0.407	0.819
27	p	Lærdal	F	All (·)	2015	2.365	0.508	1.368	3.361
28	p	Årdal	F	All (·)	2012	-26.05	3150.37	-6200.79	6148.68
29	p	Årdal	F	All (·)	2013	-5.119	0.111	-5.337	-4.901
30	p	Årdal	F	All (·)	2014	0.884	0.144	0.602	1.167
31	p	Årdal	F	All (·)	2015	2.705	0.177	2.358	3.053
32	p	All (·)	I	All (·)	All (·)	1.185	0.082	1.025	1.345
33	p	All (·)	M	Spr + Aut	All (·)	1.868	0.147	1.581	2.156
34	p	All (·)	M	Winter	All (·)	23.562	0.406	22.766	24.358
35	p	All (·)	O	All (·)	All (·)	-1.156	0.025	-1.205	-1.106
36	Psi	All (·)	F to I	Autumn	All (·)	-1.243	0.032	-1.306	-1.179
37	Psi	Aurland	F to I	Spring	All (·)	-7.218	0.067	-7.349	-7.088
38	Psi	Fortun	F to I	Spring	All (·)	-0.818	0.071	-0.958	-0.679
39	Psi	Lærdal	F to I	Spring	All (·)	-0.857	0.092	-1.037	-0.677

40	Psi	Årdal	F to I	Spring	All (·)	-0.742	0.079	-0.897	-0.587
41	Psi	All (·)	F to M	Autumn	All (·)	-1.380	0.033	-1.444	-1.315
42	Psi	All (·)	F to M	Spring	All (·)	-1.052	0.038	-1.126	-0.978
43	Psi	All (·)	F to O + O to F	Autumn	All (·)	4.168	0.032	4.104	4.231
44	Psi	All (·)	F to O + O to F	Spring	All (·)	-0.864	0.042	-0.947	-0.781
45	Psi	All (·)	I to O + O to I	Autumn	All (·)	-14.371	0.041	-14.451	-14.290
46	Psi	All (·)	I to O + O to I	Spring	All (·)	-2.010	0.036	-2.081	-1.940
47	Psi	All (·)	I to F	Autumn	All (·)	-8.909	0.096	-9.097	-8.721
48	Psi	All (·)	I to F	Spring	All (·)	-2.023	0.074	-2.168	-1.878
49	Psi	All (·)	I to M	Autumn	All (·)	-1.981	0.095	-2.168	-1.794
50	Psi	All (·)	I to M	Spring	All (·)	-0.500	0.071	-0.639	-0.360
51	Psi	All (·)	M to F	Autumn	All (·)	5.738	0.127	5.489	5.987
52	Psi	All (·)	M to F	Spring	All (·)	4.341	0.072	4.199	4.482
53	Psi	All (·)	M to I	Autumn	All (·)	3.771	0.124	3.528	4.014
54	Psi	All (·)	M to I	Spring	All (·)	-0.890	0.070	-1.027	-0.754
55	Psi	All (·)	M to O	Autumn	All (·)	18.144	0.131	17.887	18.401
56	Psi	All (·)	M to O	Spring	All (·)	-6.701	0.075	-6.848	-6.554
57	Psi	All (·)	O to M	Autumn	All (·)	11.200	0.040	11.122	11.278
58	Psi	All (·)	O to M	Spring	All (·)	-1.992	0.038	-2.066	-1.918
59	Psi	All (·)	F to O + O to F	Winter	All (·)	3.483	0.074	3.338	3.627
60	Psi	All (·)	F to I + I to F	Winter	All (·)	11.231	0.068	11.098	11.363
61	Psi	All (·)	F to M + M to F	Winter	All (·)	4.712	0.121	4.475	4.950
62	Psi	All (·)	I to M + M to I	Winter	All (·)	0.106	0.255	-0.393	0.605
63	Psi	All (·)	I to O + M to O + O to I + O to M	Winter	All (·)	4.712	0.190	4.340	5.085

Note: SE denotes the standard error for the parameter estimates. UCI/LCI denotes the upper and lower 95% confidence intervals for each parameter estimate.

Temporal model effects were defined as: time (t), year (Yr) and season (SSn). Seasons were defined accordingly, spring/summer (Spr) = WoY: 13 – 26 and autumn (Aut) = WoY: 27 – 40, except for model number 2 (Spr = WoY: 13 – 24, Aut = WoY: 25 – 40). Four spatial states were defined as the habitat zones: F = freshwater, I= inner-fjord, M = mid-fjord, O = outer-fjord, and individuals were grouped by river (Rvr).

“/” indicates predictor separation, “+” indicates grouped as a single predictor, “*” indicates interactive effect of predictor and “(·)” indicates that predictors were held constant.

Figure S7: (a) Simulated trajectories of habitat use for 1000 individual veteran migrant brown trout, from each study river and for 2013 – 2015 conditions. Values are coloured according to habitat zone (F = freshwater, I = inner-fjord, M = mid-fjord, O = outer-fjord), shaded regions represent 95 % CI resulting from 100 iterations. **(b)** Estimates of the maximum zone-specific survival rate (S_{maxZ}) presented as the fraction surviving ($S_{maxZ} = \frac{N_{Surv}}{N_{Start}}$), dependent upon selection of an individual's maximum migration extent (fjord habitat zone: inner-, mid- or outer-fjord) and their river of origin.

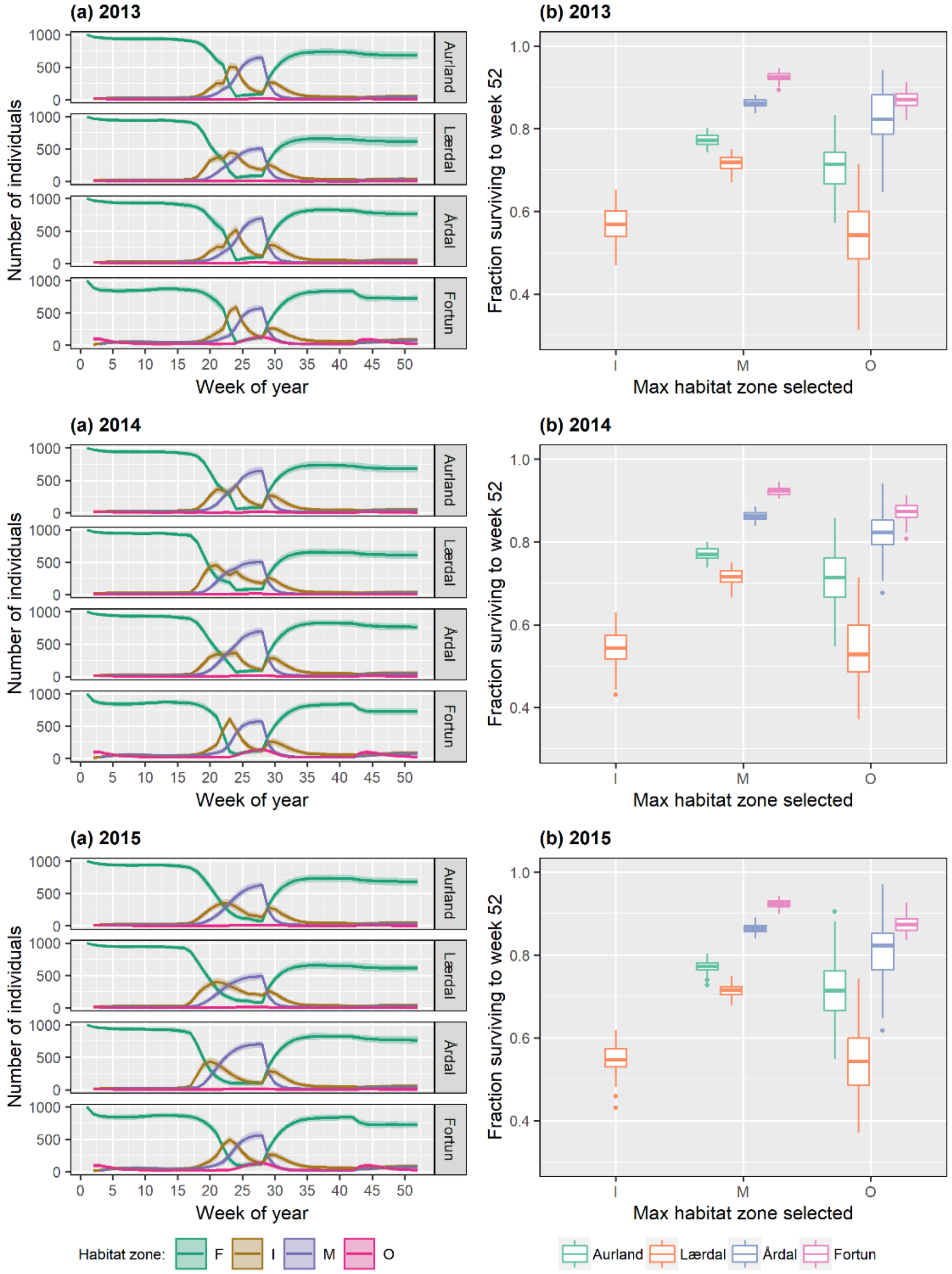

Figure S8: Simulated estimates of **(a)** individual and **(b)** population fecundity (N of eggs) of Sognefjorden veteran migrant brown trout, dependent upon selection of an individual's maximum migration extent (fjord habitat zone: inner-, mid- or outer-fjord) and their river of origin, presented for 2013 – 2015 conditions. Realised individual fecundity is estimated from the product of average expected size-specific fecundity ($\overline{Fec_{TL}}$) and the maximum zone-specific survival rate S_{maxZ}. Realised population fecundity is estimated from the product of total ($\overline{Fec_{TL}}$) and mean survival (N_{Surv}). Only individuals predicted to contribute to the spawning population are included in the realised estimates of egg numbers (from the initial population of 1000).

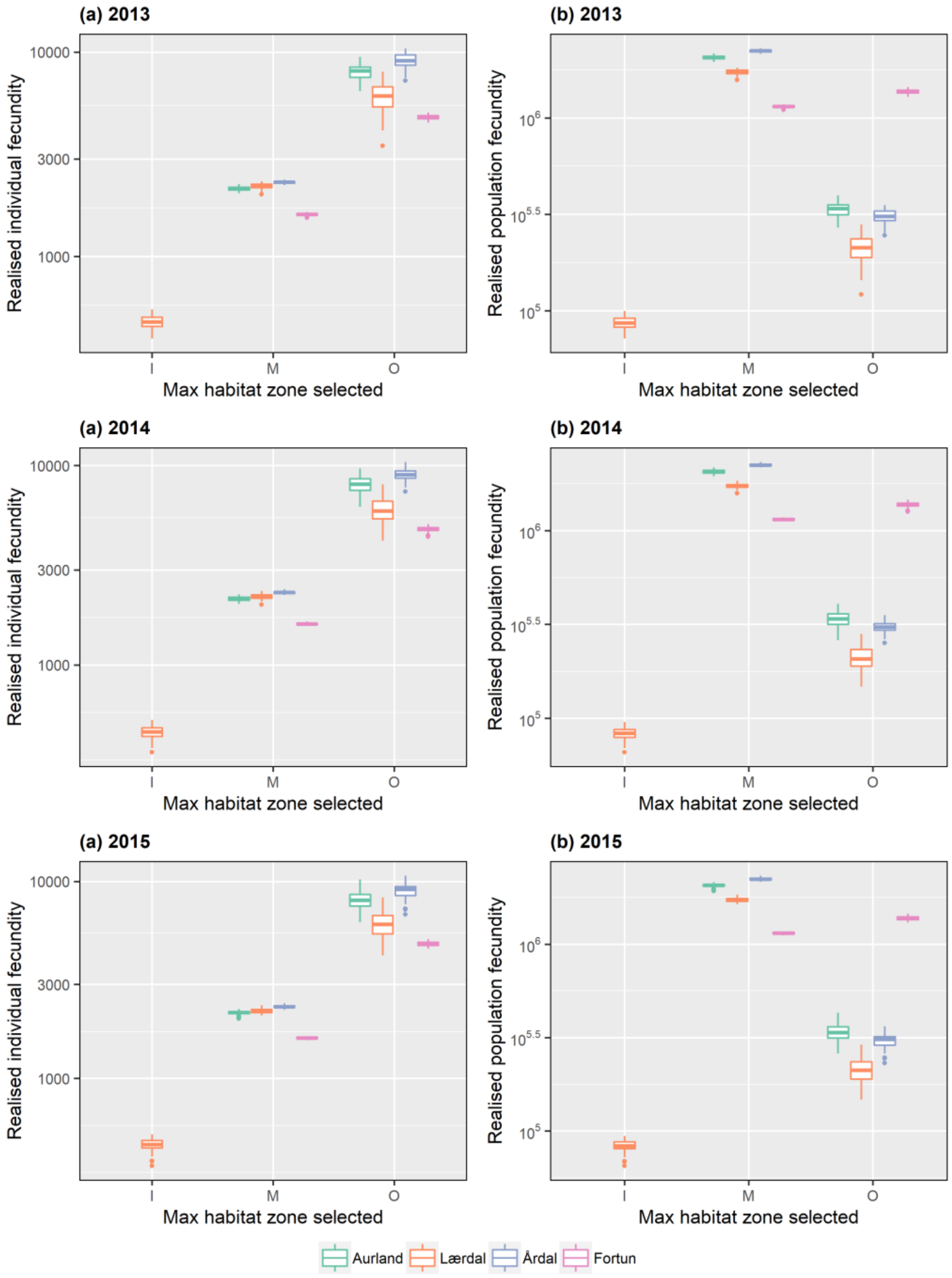

Note: Dependant on max zone reached, TL at end of growth season was estimated as $TL_{Rep} = TLe^{g_{maxZ} * \, 0.5}$, where sea growth was estimated from 2nd sea age specific growth (g_{SW2}). Size-specific estimates of fecundity ($\overline{Fec_{TL}}$) were generated according to $Fec_{TL} = e^{-4.03 + 2.74 * \, TL_{Rep}}$. Only individuals contributing to the spawning population (TL_{Rep}> 35 cm and retuned to freshwater during the period WoY 37 – 52), were included in the estimates of realised fecundity. Table 4(b) states mean values of realised individual and population fecundity for each study population.
